# Naproxen Based 1,3,4-Oxadiazole Derivatives as EGFR Inhibitors: Design, Synthesis, Anticancer, and Computational Studies

**DOI:** 10.3390/ph14090870

**Published:** 2021-08-28

**Authors:** Mohammad Mahboob Alam, Syed Nazreen, Abdulraheem S. A. Almalki, Ahmed A. Elhenawy, Nawaf I. Alsenani, Serag Eldin I. Elbehairi, Azizah M. Malebari, Mohammad Y. Alfaifi, Meshari A. Alsharif, Sulaiman Y. M. Alfaifi

**Affiliations:** 1 Department of Chemistry, Faculty of Science, Albaha University, Albaha 65731, Saudi Arabia; ahmed.elhenawy@azhar.edu.eg (A.A.E.); N.alsenani@bu.edu.sa (N.I.A.); 2 Department of Chemistry, Faculty of Science, Taif University, Taif 21974, Saudi Arabia; almalki.a@tu.edu.sa; 3 Chemistry Department, Faculty of Science, Al-Azhar University, Nasr City 11884, Egypt; 4 Department of Biology, Faculty of Science, King Khalid University, Abha 9004, Saudi Arabia; serag@kku.edu.sa (S.E.I.E.); Alfaifi@kku.edu.sa (M.Y.A.); 5 Cell Culture Laboratory, Egyptian Organization for Biological Products and Vaccines, VACSERA Holding Company, Giza 22311, Egypt; 6 Department of Pharmaceutical Chemistry, Faculty of Pharmacy, King Abdulaziz University, Jeddah 21589, Saudi Arabia; amelibary@kau.edu.sa; 7 Chemistry Department, Faculty of Applied Sciences, Umm Al-Qura University, Makkah 21421, Saudi Arabia; Maasharif@uqu.edu.sa; 8 Chemistry Department, Faculty of Science, King Abdulaziz University, Jeddah 21589, Saudi Arabia; Salfaifi@kau.edu.sa

**Keywords:** naproxen, 1,3,4-oxadiazole, cytotoxicity, EGFR, computational study

## Abstract

A library of novel naproxen based 1,3,4-oxadiazole derivatives (**8**–**16** and **19**–**26**) has been synthesized and screened for cytotoxicity as EGFR inhibitors. Among the synthesized hybrids, compound2-(4-((5-((*S*)-1-(2-methoxynaphthalen-6-yl)ethyl)-1,3,4-oxadiazol-2-ylthio)methyl)-1*H*-1,2,3-triazol-1-yl)phenol(**15**) was the most potent compound against MCF-7 and HepG2cancer cells with IC_50_ of 2.13 and 1.63 µg/mL, respectively, and was equipotent to doxorubicin (IC_50_ 1.62 µg/mL) towards HepG2. Furthermore, compound **15** inhibited EGFR kinase with IC_50_ 0.41 μM compared to standard drug Erlotinib (IC_50_ 0.30 μM). The active compound induces a high percentage of necrosis towards MCF-7, HePG2 and HCT 116 cells. The docking studies, DFT and MEP also supported the biological data. These results demonstrated that these synthesized naproxen hybrids have EGFR inhibition effects and can be used as leads for cancer therapy.

## 1. Introduction

Cancer is a devastating disease characterized by uncontrolled cell division and proliferation [1]. It is a major health burden globally and ranked second after cardiovascular diseases [2]. Although the development of anticancer drugs has advanced in recent years, due to their side effects such as drug resistance and non-differentiation between cancerous and normal cells, there is still an urgent need to discover more effective anticancer drugs which can only target cancer cells with reduced side effects [3,4].

**Figure 1:**
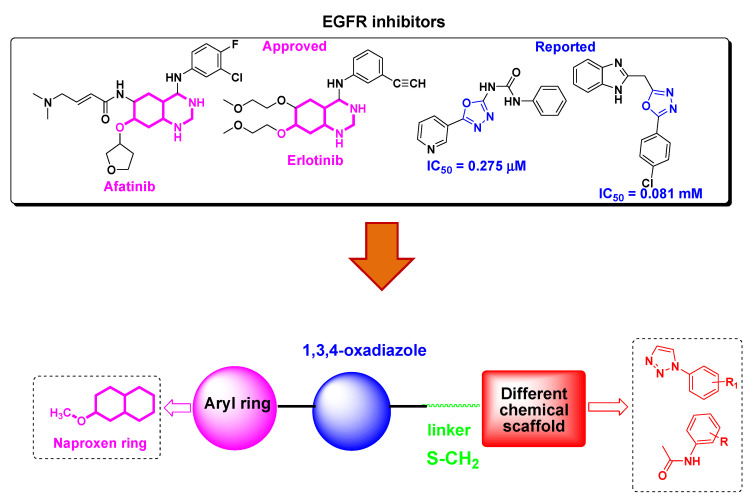
Structures of EGFR inhibitors and design of naproxen-1,3,4-oxadiazole derivatives.

Epidermal Growth Factor Receptor (EGFR) is an attractive target in cancer therapy due to its involvement in cell proliferation and differentiation [5,6]. A number of EGFR inhibitors such as Lapatinib, Gefitinib, Afatinib and Erlotinib have been developed for use in cancer therapy [7] (Figure 1) as EGFR is overexpressed or constitutively active due to mutataions in various cancers. EGFR inhibitors are small molecule tyrosine kinase inhibitors which block receptor signalling by interring with ATP binding to the receptor or monoclonal antibodies which bind to the extracellular region of the receptor, thereby inhibiting its dimerisation and autophosphorylation [8]. However, due to drug resistance and toxicity related to currently available EGFR inhibitors [9] there is an urgent need to develop new EGFR inhibitors which can overcome these side effects.

Naproxen is a well knownnon-steroidal antiinflammatory drug (NSAIDs) that acts as a COX inhibitor [10]. In addition to theiranti-inflammatory activity, naproxen derivatives have been explored for anticancer activity [11,12,13,14,15]. For example, Naproxen-based urea and propanamide derivatives have shown promising inhibition in colon cancer cells [16], protection in bladder cancer proliferation [17] and inhibitors of VEGFR-2 and histone deacetylase enzymes [18,19]. Heterocycles form the basic skeleton for a number of molecules of biological interests [20]. 1,3,4-oxadiazole is an important scaffold as it exhibits remarkable pharmacological activities such as anticancer, antiinflammatory, antimicrobial, antiviral, etc [21,22,23,24]. Furthermore, this moiety shows an antiproliferative effect through EGFR kinase inhibition (Figure 1) [25,26,27,28].

In continuation of our previously reported work to find new leads with potential anticancer activity [29,30,31], we thought to explore naproxen incorporated 1,3,4-oxadiazole derivatives for anticancer potential. To the best of our knowledge, the anticancer effect of naproxen derivatives as EGFR has not been reported yet. Therefore, we report the synthesis of naproxen based 1,3,4-oxadiazole hybrids, their cytotoxicity, in vitro EGFR inhibition and computational studies.

## 2. Materials and Methods

### 2.1. Chemistry

#### 2.1.1. General

Naproxen was purchased from Sigma Aldrich (St. Louis, MO, USA). ^1^H NMR and ^13^C NMR was carried out on a Bruker spectrometer at 850 MHz in DMSO or CDCl_3_ solvents. IR was performedon a Thermo Scientific iS 50 using the ATR technique. The melting point was recorded on a Stuart SMP40 machine. The molecular weights of the synthesized compounds were measured on a mass spectrometer (LCQ Fleet-LCF10605) and elemental analyses were performed on a LEECO Elementar Analyzer.

#### 2.1.2. Synthesis of Final Compounds (**8**–**16**)

**Scheme 1:**
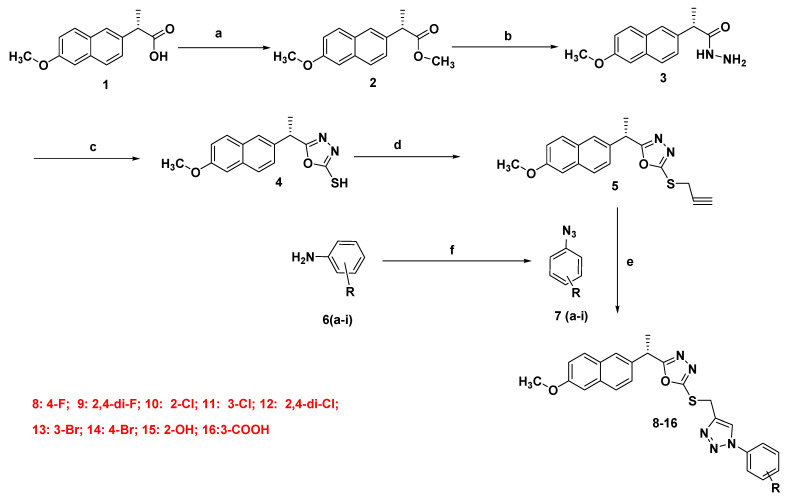
Synthetic route of target compounds **8**–**16**. Reagent and conditions: (**a**) MeOH, H_2_SO_4_, 60–70 °C, 10 h; (**b**) NH_2_·NH_2_·H_2_O, EtOH, 80–90 °C, 8 h; (**c**) i- KOH, EtOH, CS_2_, stir, 24 h, ii- 90–100 °C, 10h; (**d**) K_2_CO_3_, acetone, propargyl bromide, 50–60 °C, 4 h; (**e**) H_2_O-^t^BuOH (1:1), CuSO_4_·5H_2_O, Na ascorbate, r.t., 4–12 h; (**f**) NaNO_2_, HCl, NaN_3_, stir, 0–(−5) °C.

The target compounds (**8**–**16**) were prepared according to our previous work [31] with fewmodifications. The key intermediate **5** (0.001 mole) was charged into a round bottom flask (100 mL) followed by the addition of ter.butanol:water (2:1, 50 mL). The reaction mixture was stirred at 40–50 °C and then cooled to room temperature. Copper sulphate pentahydrate and sodium ascorbate (0.001 mole each) wereadded to the reaction mixture followed by the addition of different aromatic azides (0.0012 mole), and stirring was continued for 4–12 h. To the reaction mixture, water (50 mL) was added and products were isolated by extracting twice with dichloromethane (DCM, 50 mL), and the DCM layer was washed with water and dried over anhydrous sodium sulphate. The MDC layer was concentrated and recrystallized using MDC and cyclohexane (Scheme 1).

#### 2.1.3. Synthesis of Final Compounds Bearing Acetamide (**19**–**26**)

**Scheme 2:**
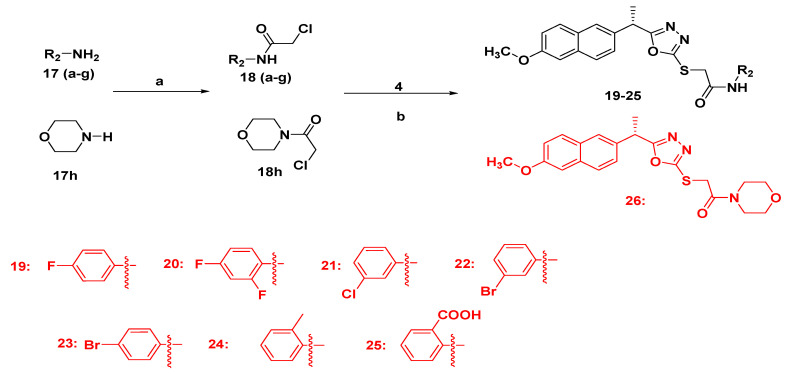
Synthetic route of target compounds **19**–**26**. Reagents and conditions: (**a**) ClCH_2_COOCH_2_CH_3_, TEA, DCM, 0–5 °C, 10 h; (**b**) acetone, K_2_CO_3_, 50–60 °C, 10–12 h.

To a clean round bottom flask (100 mL), intermediate **4** (0.001 mole) was added followed by addition of 100 mL dry acetone and anhydrous potassium carbonate (0.0012 mole). The reaction mixture was stirred for 1 h at 50–60 °C then different chloro acetamides (0.0011 mole) were added, andstirring continued untilcompletion of the reaction. After completion of the reaction, the reaction mixture was filtered and the filtrate was concentrated and poured into water (50 mL). The compounds were isolated with ethylacetate (50 mL × 2). The combined organic layer was concentrated and finally recrystallized with petroleum ether and ethyl acetate (Scheme 2). 

#### 2.1.4. Analytical Data

The analytical data including physical appearance, % yield and melting point of all final compounds **8**–**16** and **19**–**26** are present in Supplementary Material S1. The spectra (^1^H NMR, ^13^C NMR and mass) are also enclosed.

### 2.2. Biological Activities

#### 2.2.1. Antiproliferative Activity 

The cytotoxicity of the new isolated compounds was evaluated against MCF-7 (breast cells), HepG2 (Hepatocellular) and HCT-116 (colorectal cells) human cancer cells using sulphorhodamine B assay (SRB). This assay was performed as previously published [32].

#### 2.2.2. Apoptosis Using Acridine Orange/Ethidium Bromide Staining

AO and EtBr are DNA binding dyes. They have been used for detection of the morphological features of apoptotic and necrotic cells. The stainings were performed according the protocol as previously published [33].

#### 2.2.3. EGFR Kinase Activity 

In vitroEGFR inhibitory activity of most active compounds was evaluated by Enzyme Linked immunoabsorbent assay (ELISA) using a EGFR Erb B1 kinase kit (Sigma) with Erlotinib as a positive control according to the reported method [34]. Briefly, solutions of kinase/peptide and ATP were prepared prior to use. The solution on the plate were carefully mixed and incubated for 1 h at 25 °C. Then, 5 mL of this prepared solution was added into 96 well plates, incubated for 1 h and determined by an ELISA Reader (Perkin Elmer, Waltham, MA, USA). Each experiment was repeated three times and IC_50_ represented as mean ± S.E, was calculated using Graph Pad Prism 5. 

#### 2.2.4. Statistical Analysis

Data were presented as mean standard deviation unless otherwise indicated. Significance of the statistical analysis was acceptable to a level of *p* < 0.05. 

### 2.3. Computational Details and Molecular Docking

The Jaguar package used for DFT calculations also applied for ligand preparation [35]. The geometrical optimizations and chemical reactivity were estimated using B3LYP/MIDIX level using 6-311++G** basis set. The optimized geometry structures were implemented in the Maestro package, followed by minimization using an OPLS force field. TM protein was obtained in 3D crystal structure (PDB ID: 1M17), then minimized using an OPLS force field. The binding site was investigated by theheCASTp package. Glide software was utilized for the generation of the Receptor grid and Docking simulation process, and the remaining procedure was conducted as reported in previous research [31].

## 3. Results and Discussion

### 3.1. Chemistry

The synthetic path of naproxen hybrids **8**–**16** and **19**–**26** are presented in Scheme 1 and Scheme 2. The key intermediates **4** and **5** were prepared according to the reported methods with little modification [30]. Firstly, Naproxen **1** was converted into methyl ester **2** by reacting with methanol in presence of concentrated sulphuric acid as catalyst. The methyl ester **2** was refluxed with hydrazine monohydrate in methanol to yield hydrazide **3**, which was further reacted with carbon disulphide in presence of potassium hydroxide in absolute ethanol at room temperature overnight and then refluxed for 12 h to obtainthe key intermediate **4**. This key intermediate **4** was reacted with propargyl bromide in presence of potassium carbonate using dry acetone as solvent to obtainother key intermediate **5**. The target compounds **8**–**16** were synthesized via Click dipolar cycloaddition approach from **5** using different aromatic azides in presence of tert BuOH:H_2_O (1:1) as a solvent. Other target compounds **19**–**26** were obtained by S-alkylation of key intermediate **4** with different chloroacetamide using acetone and potassium carbonate. The structures of all final hybrids were confirmed by spectroscopic techniques and elemental analysis. The structure of final compounds **8**–**16** displayed characteristic peaks for 1,2,3 triazolyl protons in the range δ 8.10–8.46 ppm in ^1^H NMR and 1,3,4-oxadiazole ring in the range δ 159.46–165.89 ppm in ^13^C NMR supporting their formation. In compounds **19**–**26**, the absence of triazolyl proton which was present in **8**–**16** in ^1^H NMR and appearance of a new singlet for NH in the range δ 8.58–9.47 ppm in ^1^H NMR and downshielded C=O peaks in the range δ 171.04–176.59 ppm in ^13^C NMR confirmed the presence of NH-C=O group in hybrids **19**–**26.** The linker S-CH_2_ between heterocycles appeared in range δ 3.94–4.64 ppm as a singlet in ^1^H NMR and δ 25.84–37.69 ppm in ^13^C NMR spectra. The electron ionization mass spectra were in agreement with the structures of all final compounds.

### 3.2. In Silico ADME/Pharmacokinetics Studies

The pharmacological and pharmacokinetic properties of a compound are important parameters in drug development and discovery as theyreduce the time and cost in the development of a drug [23]. The molecule must satisfy Lipinski and Veber’s rules to be developed as an effective orally available drug. Any violation of Lipinski and Veber’s rules might lead to problems with bioavailability, permeability, solubility, etc [36]. The final compounds have been subjected toin silicoADME studies to predict their physicochemical and pharmacokinetics parameters. 

The in silico pharmacokinetics data (Table 1) showed that all the target compounds except **9**, **12**, **13** and **14** indicated easy transportation as they displayed a molecular weight below 500. These compounds depicted percentage absorption in the range of 60.19–73.62%, suggesting good absorption by the human body. The physicochemical parameters such as hydrogen bond acceptor (less than 5)/donor (less than 10) required for a drug development were found to be in the range of 5–8 and 0–2, respectively, for the final hybrids. Permeability of a drug is another important parameter which decides the fate of a drug and should be less than 5. The final compounds exhibited good permeability as indicated by logP values in the range of 2.92–4.67. However, the standard drug, doxorubicin, indicated three violations of the Lipinski rules as it exhibited MW of 543.52, nROTB = 5, HBA/HBD-12/6, TPSA = 206.07, ilogP = 2.58, low GI absorption and % abs of 37.90. These results revealed that the compounds **8**–**16** and **19**–**26** possess desired pharmacokinetics properties for the development of a lead molecule.

### 3.3. Biological Activities

#### 3.3.1. Antiproliferative Activity

**Figure 2:**
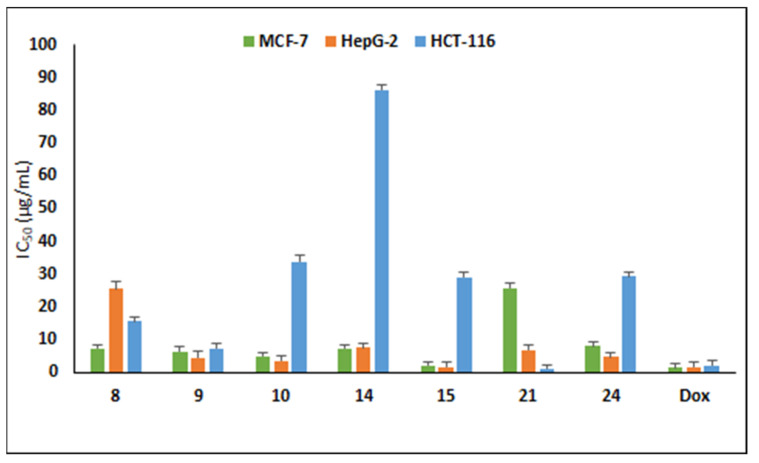
Cytotoxicity of active compounds on MCF-7, HepG2 and HCT-116 cancer cells. Data represent three independent experiments in mean ± SD. Dox: doxorubicin (positive control).

The antiproliferative activity of the target compounds was assessed on three cancer lines, MCF-7, HepG2 and HCT-116 cells using sulforhodamine assay [32]. The cytotoxic results displayed that the compounds against tested tumor cell lines showed variable cytotoxic activity (Table 2). Compound **15** bearing hydroxyl group at the *ortho* position of the triazolyl ring was the most potent compound against MCF-7 and HepG2cancer cells with IC_50_ of 2.13 and 1.63 µg/mL, respectively, and was equipotent to doxorubicin (IC_50_ 1.62 µg/mL) towards HepG2 (Figure 2). The halogen-substituted 1,2,3-triazolyl analogues displayed good cytotoxicity with IC_50_ in the range 5.05–15.84 µg/mL against MCF-7 cells and 3.53–25.49 µg/mL towards HepG2, whereas only compound **9** was active against HCT-116 with IC_50_ 7.46 µg/mL.

Naproxen-bearing oxadiazole and acetamide moieties were less effective in exerting the anticancer effect on the tested cell lines. Compound **21** possessing 3-Cl exhibited prominent antiproliferative activity with two-fold (IC_50_ 1.24 µg/mL) cytotoxicity to doxorubicin (IC_50_ 2.11 µg/mL) on colorectal HCT-116 cells, whereas compound **24** was active against MCF-7 (IC_50_ 8.15 µg/mL) and HepG2 (IC_50_ 4.88 µg/mL). Other compounds displayed moderate activity with IC_50_ less than 100 µg/mL and two compounds (**19** and **22**) were inactive towards the tested cancer cells with IC_50_ above 100 µg/mL. From these data, it can be noted that these synthesized naproxen analogues have the capability to kill cancerous cells.

#### 3.3.2. In Vitro EGFR Activity

EGFR overexpression is reported to play a significant role in the uncontrolled proliferation of different cancers.Therefore inhibition of EGFR has been a captivating target for cancer therapy. Compounds with promising cytotoxicity against the tested cell line were selected for EGFR kinase activity and werecompared with reference drug, erlotinib. From the results EGFR inhibitory activity (Table 3), all hybrids displayed remarkable inhibitory effects on EGFR with IC_50_ ranging between 0.41–7.31 μM. 1,2,3-triazole incorporated naproxen hybrids were more active in EGFR inhibition than acetamide containing naproxen hybrids. Two compounds, **15** and **10**, were the most potent compounds, with IC_50_ 0.41 μM and 0.67 μM, respectively, in inhibiting EGFR kinase compared to standard drug Erlotinib (IC_50_ 0.30 μM). Naproxen hybrids-bearing acetamide groups, **21** and **24**, revealed good inhibition with IC_50_ of 0.82 μM and 1.08 μM, respectively. It was noted that position of substituent on the phenyl ring linked to 1,2,3-triazole played significant role in EGFR inhibition. Compound **15** (2-OH) and **10** (2-Cl) bearing substituents at *ortho* position exhibited significant EGFR inhibition than *meta* substituted hybrids (**16** and **13**) and the activity was further diminished by *para* substituted hybrids (**8** and **14**). This trend in activity may be attributed to the close binding of *ortho* substituted group with the receptor. It was also observed that disubstituted hybrids (**9**, IC_50_ 0.71 μM) were more potent in EGFR inhibition than monosubstituted (**8**, IC_50_ 7.31 μM). Replacement of bromo group (**13**) with COOH group (**16**) enhanced the EGFR activity. These results demonstrated that these hybrids have EGFR inhibition effects and well correlates with the in vitro antiproliferative data.

#### 3.3.3. Apoptosis Studies

**Figure 3:**
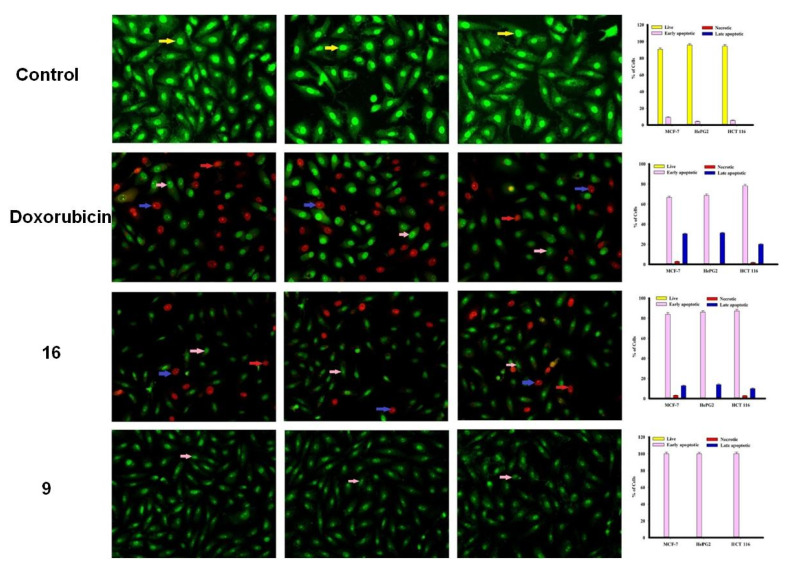

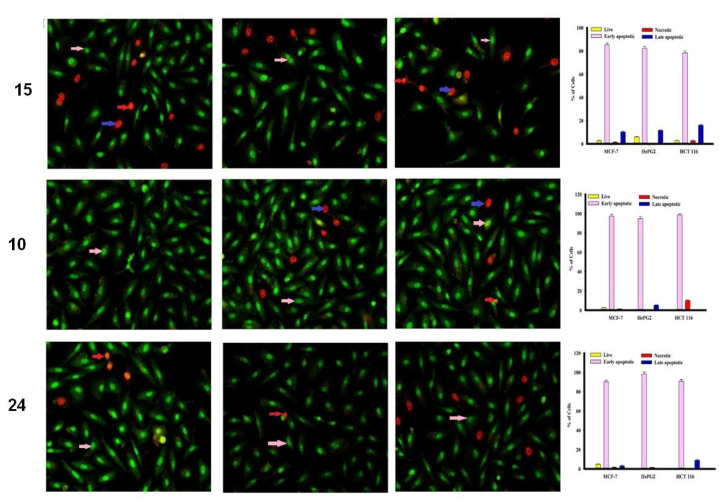
Cell apoptosis observed using fluorescence microscope. Cells were treated with compound **16**, **9**, doxorubicin and control with acridine orange-ethyl bromide staining. Cells were treated with compound **15**, **10** and **24** with acridine orange-ethyl bromide staining.

The MCF-7, HepG2 and HCT 116 cancer cells were categorized by AO/EtBr dual staining [33]. The different colors of the cells indicate different stages of apoptosis. Normal green nucleus indicates living cells; bright green nucleus with fragments of chromatin indicates early apoptotic stage; orange nuclei along with fragmented chromatin fibres shows late apoptosis and uniformly orange nuclei indicate necrotic cells. After 48 h of staining, the potent compounds were treated and examined under a fluorescent microscope. Regularly stained green with normal, round, intact nuclei and cytoplasm indicates the viability of the normal cell (control). As shown in Figure 3, compound **9** and **10** caused a significant rate of cell death in early apoptosis on all the tested three cell lines. However, the percentage of cell death decreased after treatment with compound **15** against HePG2 and HCT 116 cancer cells.

In addition, compound **16** induces a high percentage of necrosis towards MCF-7 and HePG2 cells as well as compound **15** with HCT 116 cells, while compound **9** does not cause death of the necrosis pathway in all cancer cells (Figure 3). Late apoptosis was observed at a high rate after treatment with compound **16** on MCF-7 and HePG2 cells and compound **15** induces more late apoptosis in HCT 116 cells compared to other cancer cells.

### 3.4. Computational Study

#### 3.4.1. Electronic Properties

**Figure 4:**
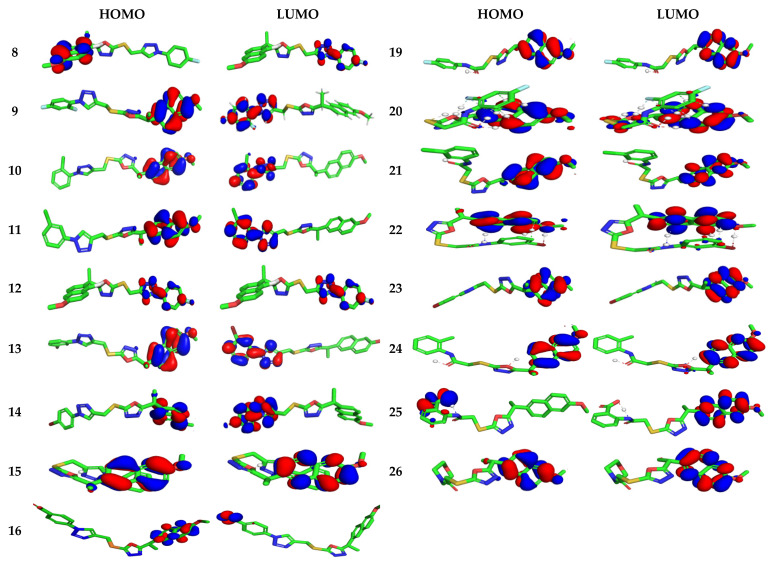
Ground state charge density of FMOs of studied derivatives with contour value = 0.032.

The highest occupied/lowest unoccupied molecular orbitals (HOMOs/LUMOs) of naproxen derivatives are probed at B3LYP/6–31G** level (Figure 4). HOMO area was observed over terminal naproxenyl groups whereas LUMO zone was found to caped over the opposite terminal scaffold in **8**–**11** and **19**–**26** compounds indicating intra-molecular charge transfer (ICT) was observed from HOMOs to LUMOs. The activity of compounds is also strictly associated to the spatial distribution of occupied molecular orbital enlightening the most credible locations in order that certainly attacked by reactive agents. The FMOs superimposed considerably, which is revealing the particularly reactive nature of the drugs. The energies of FMOs, for instance HOMO (*E_HOMO_*), LUMO (*E_LUMO_*), as well as HOMO-LUMO energy gaps (*E_gap_*) are noteworthy parameters to probe molecules’ electronic characteristics. The *E_HOMO_*, *E_LUMO_*, and *E_gap_* of recently synthesized derivatives along with reference drug are displayed in Table S1.

To determine the activity, the measurement of global chemical reactivity descriptors (GCRD) is an essential consideration which is developed as a reported reference [37,38]. In these parts, we estimated the large number of GCRD parameters specified by energy of HOMO with LUMO orbitals such aselectrophilicity index (ω), softness (S), electronegativity (χ), chemical potential (µ) as well as chemical hardness (η) with the help of HOMO/LUMO energies, vertical ionization energy (IP) and vertical electron affinity (EA). The growing HOMO energy is attributed to high efficacy for biomolecules and good electrophile parameters. In addition, decreasing LUMO-energy is related to good nucleophile character for the molecule [39]. The η of phytochemicals is interconnected to aromaticity [40]. 

#### 3.4.2. Molecular Electrostatic and Ionization Potential Maps

**Figure 5:**
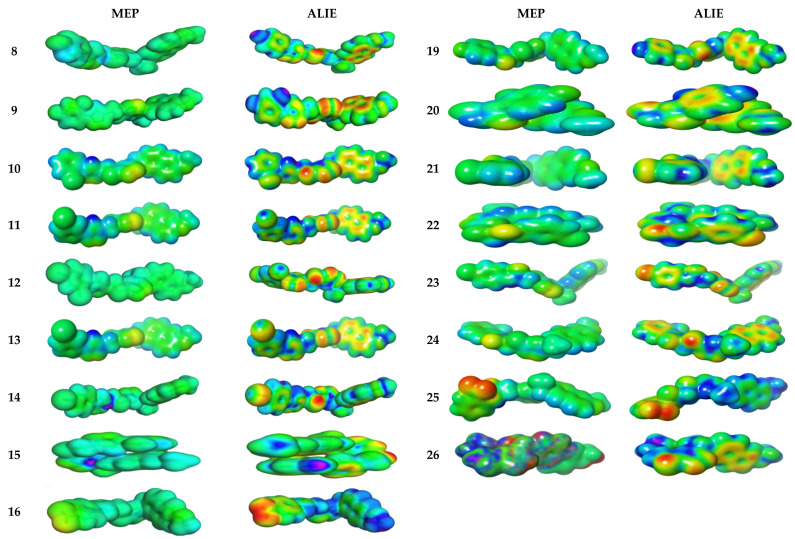
Molecular electrostatic potential surfaces views of studied derivatives with contour value = 0.032.

The molecular electrostatic potential (MEP) is used to explore the reactivity of synthesized hybrids and is measured experimentally by diffraction approaches [41]. MEP maps are important to visualize a region charged within molecules. In Figure 5, the MEP map of the **8**–**11** and **19**–**26** compounds is shown in color visualization. Red color defines high negative potential regions that benefit from electrophilic attacks, while blue indicates high positive potential regions that prefer nucleophilic attacks. The MEP is reduced by ordering green > blue > yellow > orange > red, the red indicates a more intense repulsion while blue defines sufficient attraction. The difference between colors provides useful information about the ability of these atomic sites to produce electrophilic and nucleophilic attacks with the receptor. The swelling of green zones may be due to the nucleophilicity power of the compounds. As expected in **8**–**11** and **19**–**26** compounds, the green and yellow regions, respectively, are shielded upon aromatic and triazole rings, respectively [42]. 

ALIE (average local ionization energy map) is more preferable than MEP for prediction of favorable molecular sites to electrophilic attacks. The red color is bounded to both nitrogen atoms in triazole moiety in all compounds **8**–**11** and **19**–**26**. The electron density in yellow color has high spreading over all molecular backbone for all hybrids **8**–**11** and **19**–**26**. The blue color is more localized in terminal phenyl groups opposite to naproxenyl moiety; thereby, these groups are prone to nucleophilic attacks. The most hypersensitive action is displayed in compounds **8**, **9**, **10**, **15**, **16** and **24** towards electrophilic attackviasurrounding biological media. 

#### 3.4.3. Molecular Docking

**Figure 6:**
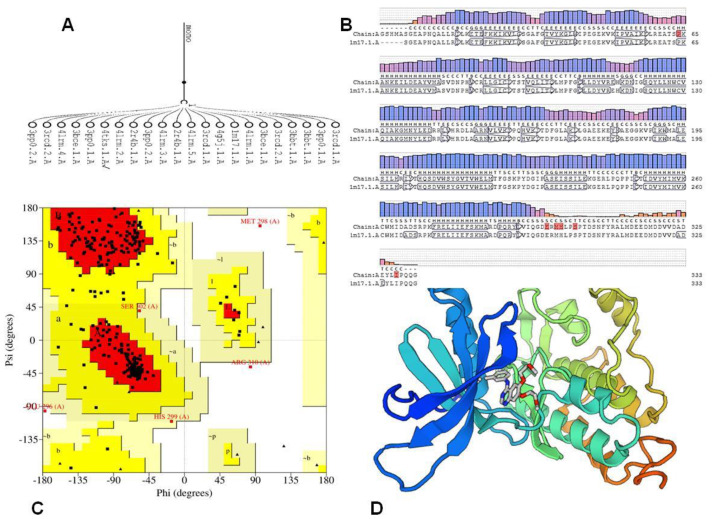
(**A**) Alignment of EGFR sequence with rough models. (**B**) Alignment of Homologues model of 1M17. (**C**) generated model. (**D**) Ramachandran Plot: (A,B,L) Most favoured regions in [red] with 86.1%, (a,b,l,p) Additional allowed regions in yellow with 12.2%, generously allowed regions 0.7 [pale yellow], Disallowed regions [white].

The comparative study for protein sequences using sequence alignment offers valuable data throughout structural and functional analysis through revealing sequence of the structure–function relationships. Three-dimensional (3D) representation of the EGFR kinase domain was established. The mGen THERADER was applied togenerate a 3D loop structure of PDB 1M17 complexed with Erlotinib, which was used as framework for docking experiment [43]. The X-ray structures of the rough model for the EGFR template werecompared with the target protein using comparative SWISS MODEL [44]. The 3D model was further checked and verified using Ramachandran plot at PROCHECK [45]. The amino acids of EGFR active pocket (Lys721, Phe699, Val702, Cys773, Leu820, Asn818, Asp783, Asn784, Gln958, Gln962, Met96, Met964, Leu97722) were identified using CASTp [44] (Figure 6).

In order to find suitable binding modes of the investigated naproxenyl oxadiazols hybrids **8**–**11** and **19**–**26** against EGFR kinase, docking studies were simulated using the Schrödinger software [46]. The reproducibility of the software was validated through redocking of hybrids. The investigated hybrids were successfully redocked with a root mean square deviation (RMSD) less than 2 Å. The fingerprint for ligand-protein-interactions was estimated based on the docking score ΔG. All calculated energies of the docking simulation were summarized in Table 4. The hybrids **8**–**11** and **19**–**26** were successfully capped into the active zone of the enzymes. The docked poses of complexes were obtained and used for energy-minimized by a molecular-mechanics (Oples3e) force field, until neutralization. The poses were selected depending on the lowest binding free energy ΔG with the lowest root means quart deviation (RMSD) between the pose before and after refinement. Finally, the highest scoring function for the tested compounds was applied to evaluate the binding affinities of the tested compounds.

**Figure 7:**
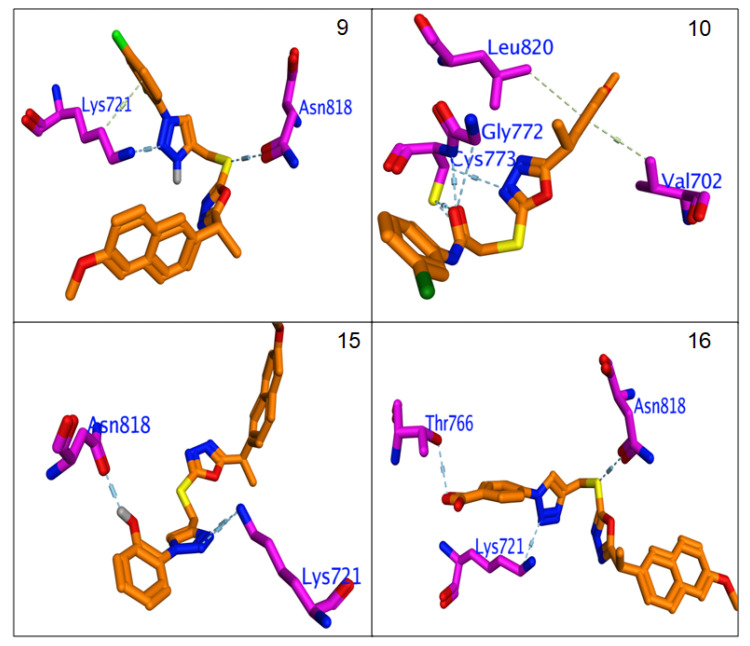
Binding interactions of most potent Naproxen-1,2,3-triazole hybrids **9**, **10**, **15** and **16** against EGFR synthase (1M17).

**Figure 8:**
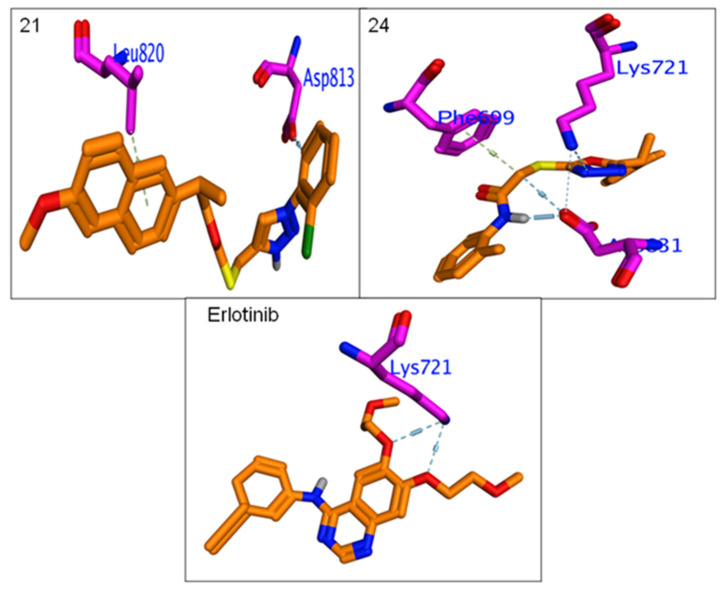
Binding interactions of most potent Naproxen-acetamide hybrids **21**, **24** andreference drug, Erlotinibagainst EGFR synthase (1M17).

All hybrids **8**–**11** and **19**–**26** showed higher binding affinity (−6.3 to −7.14 Kcal/mol.) than Erlotinib (−6.2 Kcal/mol.). The calculated RMSDs are lower than 2 degrees, which reflects the accuracy of the docking process. The most potent compounds, **9**, **10**, **12**, **15**, **16**, **21** and **24**, were selected to clarify their molecular interaction mechanisms for in-depth analysis. The compounds **9**, **10**, **15**, **16** and **24** have promising binding affinities through formation higher number of H & π-π interaction (3 to 5) against EGFR active when compared to Erlotinib (two H-bond),while other compounds **15** and **21** showed the same number of interactions with template drug. It was found that most potent compounds, **9**, **10**, **12**, **15**, **16**, **21** and **24**, were stabilized in binding pocket similar to Erlotinib. The compounds **9**, **15**, **16** and **24** were arranged in parallel style with important Lys7251 to form a strong H-bond interaction, while compounds **9**, **12** and **15** capped binding pocket by formation other important H-bond with Asn818 and localized by perpendicular mode with their amino acid (Figure 7 and Figure 8). The presence of the hydrophobic interactions observed in **9**, **10**, **21** and **24** represents the higher binding affinities through the receptor-ligands with ranged ligand efficiency (−2.95 to −6.74).

## 4. Conclusions

A library of novel naproxen based 1,3,4-oxadiazole hybrids (**8**–**16** and **19**–**26**) has been synthesized and screened for cytotoxicity as EGFR inhibitors. Among the synthesized hybrids, compound2-(4-((5-((*S*)-1-(2-methoxynaphthalen-6-yl)ethyl)-1,3,4-oxadiazol-2-ylthio)methyl)-1*H*-1,2,3-triazol-1-yl)phenol (**15**) was the most potent compound against MCF-7 and HepG2 cancer cells with IC_50_ of 2.13 and 1.63 µg/mL, respectively, and was equipotent to doxorubicin (IC_50_ 1.62 µg/mL) towards HepG2. Furthermore, this compound **15** inhibited EGFR kinase with IC_50_ 0.41 μM compared to standard drug Erlotinib (IC_50_ 0.30 μM). The active compound induces a high percentage of necrosis towards MCF-7, HePG2 and HCT 116 cells. The docking studies, DFT and MEP also supported the biological data. From these results, it can be inferred that these naproxen hybrids have the potential to inhibit EGFR kinase by binding with adenosine triphosphate (ATP) through competitive inhibition in tyrosine kinase domain.

## Figures and Tables

**Table 1:** Physicochemical and pharmacokinetic properties of final compounds **8**–**16** and **19**–**26**.

	Lipinski Parameters									
	MW ^a^	HBA ^b^	HBD ^c^	LogP ^d^	Violations	nROTB ^e^	TPSA ^f^	%ABS ^g^	BBB ^h^	GI ABS ^i^
**8**	461.51	7	0	4.41	0	7	104.16	73.06	No	Low
**9**	479.5	8	0	4.18	1	7	104.16	73.06	No	Low
**10**	477.97	6	0	4.30	0	7	104.16	73.06	No	Low
**11**	477.97	6	0	4.47	0	7	104.16	73.06	No	Low
**12**	512.41	6	0	4.47	2	7	104.16	73.06	No	Low
**13**	522.42	6	0	4.59	2	7	104.16	73.06	No	Low
**14**	522.42	6	0	4.67	2	7	104.16	73.06	No	Low
**15**	459.52	7	1	4.21	0	7	124.39	66.08	No	Low
**16**	487.53	8	1	3.49	0	8	141.46	60.19	No	Low
**19**	437.49	6	1	3.63	0	8	102.55	73.62	No	High
**20**	455.48	7	1	4.02	0	8	102.55	73.62	No	Low
**21**	453.94	5	1	3.71	0	8	102.55	73.62	No	High
**22**	498.39	5	1	3.88	0	8	102.55	73.62	No	Low
**23**	498.39	5	1	3.87	0	8	102.55	73.62	No	Low
**24**	433.52	5	1	3.9	0	8	102.55	73.62	No	High
**25**	463.51	7	2	2.92	0	9	139.85	60.75	No	Low
**26**	413.49	6	0	3.81	0	7	102.99	73.46	No	High

^a^ Molecular weight; ^b^ Hydrogen Bond Acceptor; ^c^ Hydrogen Bond Donor; ^d^ Partition Coefficient; ^e^ Number of rotable bonds; ^f^ Topological Polar Surface Area; ^g^ Absorption%; ^h^ Blood Brain Barrier; ^i^ Gastro-intestinal absorption.

**Table 2:** Cytotoxicity of final compounds **8**–**16** and **19**–**26**.

IC_50_(μg/mL)			
Compounds	MCF-7	HepG2	HCT-116
**8**	7.38	25.49	15.66
**9**	6.47	4.52	7.46
**10**	5.05	3.53	33.91
**11**	60.45	37.88	83.92
**12**	45.7	26.37	28.15
**13**	15.84	7.07	29.28
**14**	7.50	7.63	86.1
**15**	2.13	1.63	29.1
**16**	10.44	10.67	8.36
**19**	387.46	829.66	1107.03
**20**	32.65	31.32	73.42
**21**	25.90	7.00	1.24
**22**	97.29	159.2	20.24
**23**	25.56	14.97	35.15
**24**	8.15	4.88	29.34
**25**	29.79	28.02	29.21
**26**	22.28	23.64	11.09
Doxorubicin	1.41	1.62	2.11

Data represent the mean values of three independent experiments performed in triplicate; Breast cancer cells (MCF-7), Liver cancer cells (HepG2) and colorectal cancer cells (HCT-116) were used. Doxorubicin was used as a positive control.

**Table 3:** Inhibitory activity of derivatives on EGFR.

Compounds	EGFR (IC_50_, μM)
**8**	7.31 ± 0.25
**9**	0.71 ± 0.17
**10**	0.67 ± 0.33
**13**	3.42 ± 0.11
**14**	5.12 ± 0.21
**15**	0.41 ± 0.12
**16**	2.27 ± 0.17
**21**	0.82 ± 0.28
**24**	1.08 ± 0.31
Erlotinib	0.30 ± 0.09

IC_50_ is the concentration required to inhibit the enzyme activity by 50%; Each value is the mean ± S.E of three independent experiments.

**Table 4:** Docking scores (kcal/mol) of Erlotinib, **8**–**11** and **19**–**26** hybrids.

	ΔG	Rmsd	E.vdw	E.Int	*E._H.B_*	Eele	LE
Erlotinib	−6.27	1.43	−33.45	−69.42	−10.72	−37.91	−4.38
**8**	−6.61	1.92	58.42	−83.02	−9.87	−42.21	−3.45
**9**	−6.33	1.15	30.35	−43.38	−11.56	−42.17	−2.95
**10**	−6.53	1.76	34.61	−51.40	−11.27	−36.07	−3.72
**11**	−6.69	1.28	54.56	−47.98	−9.74	−40.32	−2.04
**12**	−6.58	1.38	21.52	−35.34	−10.71	−41.74	−4.76
**13**	−6.83	1.76	56.07	−71.30	−12.22	−36.19	−3.88
**14**	−6.69	1.42	54.59	−33.93	−9.62	−38.08	−1.95
**15**	−7.14	1.22	45.95	−77.82	−10.91	−41.24	−5.87
**16**	−7.00	1.56	−12.27	−44.06	−10.55	−41.11	−2.73
**19**	−6.52	1.86	3.54	−62.10	−9.17	−38.25	−3.50
**20**	−6.39	1.15	8.52	−65.83	−10.70	−39.01	−2.97
**21**	−6.60	0.98	−9.42	−43.49	−10.10	−39.53	−6.74
**22**	−6.33	1.52	−8.45	−29.15	−9.14	−34.41	−4.17
**23**	−6.33	1.55	−2.47	−55.54	−10.02	−32.07	−4.09
**24**	−6.68	1.46	−2.82	−85.90	−8.95	−37.87	−4.56
**25**	−6.33	1.74	−77.81	−41.75	−10.47	−39.31	−3.64
**26**	−6.32	1.24	56.04	−34.78	−11.08	−38.89	−2.82

ΔG: Free binding energy of the ligand from a given conformer, E.Int: Affinity binding energy of hydrogen bond interaction with receptor, *E._H.B_*: Hydrogen bonding energy between protein and ligand. Eele: Electrostatic interaction with the receptor, Evdw: Van der Waals energies between the ligand and the receptor, L.E.: Ligand efficiency.

## Data Availability

Data is contained within the article and supplementary material.

## Supplementary Materials

The analytical data, physical appearance, melting point, %yield, ^1^H NMR, ^13^C NMR and Mass spectra of final derivatives are available online at https://www.mdpi.com/article/10.3390/ph14090870/s1, Figures S1–S17: ^1^H NMR spectra, Figures S18–S34: ^13^C NMR spectra, Figures S35–S50: Mass spectra.
