# Supplementary material for: Naproxen Based 1,3,4-Oxadiazole Derivatives as EGFR Inhibitors: Design, Synthesis, Anticancer, and Computational Studies
Source: Pharmaceuticals (Basel). 2021 Aug 28;14(9):870. doi: 10.3390/ph14090870 (PMC8469912; doi:10.3390/ph14090870)
Supplement: Supplementary file 1 [file pharmaceuticals-14-00870-s001.zip › pharmaceuticals-1337196-supplementary.pdf]

SUPPLEMENTARY MATERIAL

Naproxen based 1,3,4-oxadiazole derivatives as EGFR inhibitors: Design, Synthesis, Anticancer, and Computational Studies

S1. Characterization data of final compounds 8-16 and 19-26

4-((5-((S)-1-(2-methoxynaphthalen-6-yl)ethyl)-1,3,4-oxadiazol-2-ylthio)methyl)-1-(4-fluorophenyl)-1H-1,2,3-triazole (8)

M.p: 99-100; yield:70%; IR (ν, cm^{-1}): 3086, 2939, 1630, 1604, 1592, 1483, 1460, 1390, 1260, 1227, 1213, 1151, 1067, 1041, 1027, 864, 814, 780; ^{1}H NMR (850 MHz, $CDCl_3$): δ 1.83 (d, 3H, $-CH_3$), 3.93 (s, 3H, O-CH_3), 4.46 (brs, 1H, -CH), 4.60 (s, 2H, S-CH_2), 7.11 (d, *J* = 7.6 Hz, 1H, 1 × CH), 7.16 (dd, *J* = 4.56, 8.50 Hz, 1H, 1 × CH), 7.20 (t, *J* = 8.50 Hz, 2H, 2 × CH), 7.37 (d, *J* = 8.50 Hz, 1H, 1 × CH), 7.65-7.72 (m, 5H, 5 × CH), 8.26 (s, 1H, 1 × CH).^{13}C NMR (213 MHz, $CDCl_3$,): δ 19.24, 26.64, 37.50, 55.35, 105.61, 116.72, 116.83, 119.34, 122.77, 125.72, 125.94, 127.61, 129.31, 133.90, 134.75, 157.93, 161.87, 163.04. ESI MS: 462 $[M+H]^+$ $C_{24}H_{20}N_5O_2S$(Calcd): C, 62.46; H, 4.37; N, 15.17; O, 6.93; S, 6.95. Obsd:C,62.44, H, 4.38: N,15.19; O, 6.96; S, 6.94

4-((5-((S)-1-(2-methoxynaphthalen-6-yl)ethyl)-1,3,4-oxadiazol-2-ylthio)methyl)-1-(2,4-difluorophenyl)-1H-1,2,3-triazole (9)

M.p: 119-120, yield: 72%; IR (ν, cm^{-1}): 3090, 2985, 1605, 1575, 1506, 1518, 1476, 1456,1263, 1177, 1146, 1105, 1088, 1045, 958, 855, 823, 771; ^{1}H NMR (850 MHz, $CDCl_3$): **δ** 1.83 (d, *J* = 7.60 Hz, 3H, $-CH_3$), 3.93 (s, 3H, -O-CH_3), 4.47 (brs, 1H, -CH), 4.61 (brs, 2H, -S-CH_2-), 7.05-7.06 (m, 2H, 2 × CH), 7.11 (d, *J* = 7.60 Hz, 1H, 1 × CH), 7.17 (d, *J* = 7.60 Hz, 1H, 1 × CH), 7.37-7.38 (m, 1H, 1 × CH), 7.67-7.72 (m, 3H, 3 × CH), 7.85 (d, *J* = 8.50 Hz, 1H, 1 × CH), 8.34 (s, 1H, 1 × CH). ^{13}C NMR (213 MHz, $CDCl_3$): δ 19.27, 26.62, 37.49, 55.34, 105.34, 105.45, 105.60, 112.54, 112.64, 119.31, 125.73, 125.92, 127.59, 128.86, 129.31, 133.89, 134.79, 157.90, 161.98, 163.10. ESI MS: 480 $[M+H]^+$ $C_{24}H_{19}N_5O_2S$(Calcd): C, 60.12; H, 3.99; N, 14.61; O, 6.67; S, 6.69. Obsd:C,60.09, H, 4.01: N,14.58; O, 6.65; S, 6.67

4-((5-((S)-1-(2-methoxynaphthalen-6-yl)ethyl)-1,3,4-oxadiazol-2-ylthio)methyl)-1-(2-chlorophenyl)-1H-1,2,3-triazole (10)

M.p: 90-92; yield:82 %; IR(ν, cm^{-1}): 3082, 2938, 1605, 1593, 1571, 1481, 1462, 1394, 1263, 1177, 1144, 1069, 1043, 1027, 895, 857, 784, 676; ^{1}H NMR (850 MHz, $CDCl_3$): δ 1.84 (d, *J* = 7.61 Hz, 3H, CH_3), 3.93 (s, 3H, $-OCH_3$), 4.47 (brs, 1H, -CH), 4.60 (s, 2H, -S-CH_2-), 7.11 (d, *J* = 8.50 Hz, 1H, 1 × CH), 7.16 (d, *J* = 7.60 Hz, 1H, 1 × CH), 7.32-7.78 (m, 8H, 8 × CH), 8.33 (s, 1H, 1 × CH). ^{13}C NMR (213 MHz, $CDCl_3$): δ 19.22, 26.61, 37.51, 55.35, 105.61, 118.78, 119.34, 121.01, 125.72, 125.94, 127.62, 128.86, 128.93, 129.30, 130.90, 133.90,

134.73, 135.71, 157.91, 161.48, 162.63. ESI MS: 478 $[M+H]^+$, 480 $[M+H+2]^+$ $C_{24}H_{20}N_5O_2S$(Calcd): C, 60.31; H, 4.22; N, 14.65; O, 6.69; S, 6.71. Obsd: C,60.28, H, 4.24: N,14.63; O, 6.73; S, 6.72

4-((5-((S)-1-(2-methoxynaphthalen-6-yl)ethyl)-1,3,4-oxadiazol-2-ylthio)methyl)-1-(3-chlorophenyl)-1H-1,2,3-triazole (11)

Semisolid; yield:73 %; IR(ν, cm^{-1}): 3078, 2935, 1604, 1581, 1576, 1489, 1651, 1390, 1261, 1114, 1064, 857; ^{1}H NMR (850 MHz, $CDCl_3$): δ 1.83 (d, *J* = 8.50 Hz, 3H, $-CH_3$), 3.93 (s, 3H, $-OCH_3$), 4.45 (q, *J* = 8.50 Hz, 1H, C-H), 4.61 (s, 2H, $-S-CH_2-$), 7.11 (s, 1H, Ar-H), 7.15-7.16 (m, 1H, 1 × CH), 7.36 (d, *J* = 8.5Hz, 1H, 1 × CH), 7.42-7.44 (m, 1H, 1 × CH), 7.46 (t, *J* = 8.5 Hz, 1H, 1 × CH),7.56-7.57 (m. 2H, 2 × CH), 7.67 (s, 1H, 1 × CH), 7.70-7.71 (m, 2H, 2 × CH), 8.10 (s, 1H, 1 × CH). ^{13}C NMR (213 MHz, $CDCl_3$): δ 19.34, 26.17, 37.48, 55.34, 105.62, 119.32, 130.75, 133.34, 134.68, 134.87, 142.70, 157.75, 163.99. ESI MS: 478 $[M+H]^+$, 480 $[M+H+2]^+$ $C_{24}H_{20}N_5O_2S$(Calcd): C, 60.31; H, 4.22; N, 14.65; O, 6.69; S, 6.71. Obsd:C, 60.30; H, 4.23; N, 14.64; O, 6.67; S,6.73

4-((5-((S)-1-(2-methoxynaphthalen-6-yl)ethyl)-1,3,4-oxadiazol-2-ylthio)methyl)-1-(2,4-dichlorophenyl)-1H-1,2,3-triazole (12)

M.p: 102-103; yield:80%; IR (ν, cm^{-1}): 3050, 2991, 2938, 1604, 1570, 1497, 1474, 1660, 1393, 1262, 1229, 1143, 1119, 1071, 1039, 1026, 894, 857, 822; ^{1}H NMR (850 MHz, $CDCl_3$): δ 1.84 (d, *J* = 8.50 Hz, 3H, $-CH_3$), 3.93 (s, 3H, $-OCH_3$), 4.47 (brs, s, 1H, C-H), 4.61 (s, 2H, -S-CH_2-), 7.11 (d, *J* = 7.60 Hz, 1H, 1 × CH), 7.15-7.17 (m, 1H, 1 × CH), 7.37 (d, *J* =8.5 Hz, 1H, 1 × CH), 7.41 (d, *J* = 7.6 Hz, 1H, 1 × CH), 7.50 (d, *J* = 8.5Hz, 1H, 1 × CH), 7.59 (s, 1H, 1 × CH), 7.67 (s, 1H, 1 × CH), 7.69-7.71 (m, 2H, 1 × CH), 8.26 (s, 1H, 1 × CH).^{13}C NMR (213 MHz, $CDCl_3$): δ 19.22, 26.63, 37.48, 55.35, 105.59, 119.33, 125.71, 125.92, 127.61, 128.26, 128.30, 128.85, 129.29, 130.62, 133.89, 136.35, 157.91, 160.34, 163.70. ESI MS: 512 $[M+H]^+$, 514 $[M+H+2]^+$ $C_{24}H_{19}N_5O_2S$(Calcd): C, 56.26; H, 3.74; N, 13.67; O, 6.24; S, 6.26. Obsd: C,56.29, H, 3.70: N,13.65; O, 6.28; S, 6.27

4-((5-((S)-1-(2-methoxynaphthalen-6-yl)ethyl)-1,3,4-oxadiazol-2-ylthio)methyl)-1-(3-bromophenyl)-1H-1,2,3-triazole (13)

M.p: 118-120; yield:86%; IR (ν, cm^{-1}): 3086, 2939, 1604, 1504, 1483, 1461,1439, 1260, 1237, 1213, 1150, 1067, 1041, 1027, 923, 889, 865, 814, 780, 769, 674; ^{1}H NMR (850 MHz, $CDCl_3$): δ1.84 (d, *J* = 8.51 Hz, 3H, $-CH_3$), 3.93 (s, 3H, $-OCH_3$),4.48 (brs, 1H, CH), 4.61 (s, 2H, S-CH_2). 7.11-17 (m, 2H, 2 × CH), 7.37-7.39 (m, 2H, 2 × CH), 7.56-7.72 (m, 5H, 5 × CH), 7.93 (s, 1H, 1 × CH), 8.29 (s, 1H, 1 × CH).^{13}C

NMR (213 MHz, $CDCl_3$): δ 19.23, 26.57, 37.51, 55.35, 105.61, 119.34, 123.42, 123.82, 125.72, 125.96, 127.62, 129.30, 131.14, 131.92, 133.90, 134.67, 157.91, 160.35, 163.72. ESI MS: 522 $[M+H]^+$, 524 $[M+H+2]^+$ $C_{24}H_{20}N_5O_2S$(Calcd): C, 55.18; H, 3.86; N, 13.41; O, 6.13; S, 6.14. Obsd: C,55.15, H, 3.88: N,13.40; O, 6.17; S, 6.15

4-((5-((S)-1-(2-methoxynaphthalen-6-yl)ethyl)-1,3,4-oxadiazol-2-ylthio)methyl)-1-(4-bromophenyl)-1H-1,2,3-triazole (14)

M.p: 160-161; yield:81%; IR (ν, cm^{-1}): 3085, 2938, 1606, 1578, 1496, 1486, 1459, 1393, 1260, 1228, 1215, 1192, 1154, 1067, 1029, 987, 813; ^{1}H NMR (850 MHz, $CDCl_3$): δ 1.75 (brd s, 3H, $-CH_3$), 3.84 (s, 3H, $-O-CH_3$), 4.37 (brs, 1H, -CH), 4.51 (s, 2H, $S-CH_2-$), 7.02 (s, 1H, 1 × CH), 7.08 (d, *J* = 8.5 Hz, 1H, 1 × CH), 7.29 (d, *J* = 8.5 Hz, 1H, 1 × CH), 7.48 (d, *J* = 8.5 Hz, 2H, 2 × CH), 7.53 (d, *J* = 8.5Hz, 2H, 2 × CH), 7.59 (s, 1H, 1 × CH), 7.61-7.63 (m, 2H, 2 × CH), 8.20 (s, 1H, 1 × CH) .^{13}C NMR (213 MHz, $CDCl_3$): δ 19.20, 26.93, 37.50, 55.36, 105.60, 119.36, 122.51, 125.72, 125.94, 127.62, 129.31, 132.98, 133.90, 134.68, 157.93, 159.46, 163.40. ESI MS: 522 $[M+H]^+$, 524 $[M+H+2]^+$ $C_{24}H_{20}N_5O_2S$(Calcd): C, 55.18; H, 3.86; N, 13.41; O, 6.13; S, 6.14. Obsd: C,55.15, H, 3.87: N,13.39; O, 6.15; S, 6.13.

2-(4-((5-((S)-1-(2-methoxynaphthalen-6-yl)ethyl)-1,3,4-oxadiazol-2-ylthio)methyl)-1H-1,2,3-triazol-1-yl)phenol (15)

M.p: 90-92; yield: 70%; IR (ν, cm^{-1}): 3150, 3045, 2937, 1605, 1506, 1480, 1457,1393, 1262, 1228, 1157, 1044, 1027, 895, 856, 822, 753; ^{1}H NMR (850 MHz, $CDCl_3$): δ 1.85 (brs, 3H, $-CH_3$), 3.93 (s, 3H, $-OCH_3$), 4.47 (brs, 1H, -CH), 4.62 (s, 2H, $S-CH_2-$), 7.00 (t, *J* = 8.50 Hz, 1H, 1 × CH), 7.12 (s, 1H, 1 × CH), 7.17 (d, *J* = 8.50 Hz, 1H, 1 × CH), 7.19 (d, *J* = 8.50 Hz, 1H, 1 × CH), 7.32 (t, *J* = 8.50 Hz, 1H, 1 × CH), 7.37-7.38 (m, 2H, 2 × CH), 7.68-7.72 (m, 3H, 3 × CH), 8.46 (s, 1H, 1 × CH), 9.70 (s, 1H, Ar-OH). ^{13}C NMR (213 MHz, $CDCl_3$): δ 19.22, 25.84, 37.52, 55.35, 105.61, 119.35, 119.49, 120.41, 125.71, 125.96, 127.63, 128.30, 129.30, 129.85, 133.92, 134.68, 149.20, 157.93, 162.15, 164.50. ESI MS: 460 $[M-H]^+$, $C_{24}H_{21}N_5O_3S$(Calcd): C, 62.73; H, 4.61; N, 15.24; O, 10.45; S, 6.98. Obsd:C,62.71, H, 4.63: N,15.22; O, 10.49; S, 6.99.

3-(4-((5-((S)-1-(2-methoxynaphthalen-6-yl)ethyl)-1,3,4-oxadiazol-2-ylthio)methyl)-1H-1,2,3-triazol-1-yl)benzoic acid (16)

M.p: 184-185; yield:89%; IR (ν, cm^{-1}): 3155, 3085, 2939, 1690, 1633, 1606, 1593, 1483, 1459, 1392, 1264, 1152, 1067, 813; ^{1}H NMR (850 MHz, $CDCl_3$): δ 1.84 (d, *J* = 7.6 Hz, 3H, CH-CH3), 3.92 (s, $-OCH_3$, 3H), 4.47-4.49 (m, 1H, C-H), 4.64 (s, 2H, $S-CH_2-$), 7.10 (d, *J* = 7.6

Hz, 1H, 1 × CH), 7.15 (s, 1H, 1 × CH), 7.38 (s, 1H, 1 × CH), 7.64-7.72 (m, 4H, 4 × CH), 8.04 (d, *J* = 8.50 Hz, 1H, 1 × CH), 8.18 (d, *J* = 8.50 Hz, 1H, 1 × CH), 8.37 (s, 1H, 1 × CH), 8.44 (s, 1H, Ar-COOH). ^{13}C NMR (213 MHz, $CDCl_3$): δ 19.25, 26.64, 37.49, 55.34, 105.60, 119.32, 121.78, 125.43, 125.71, 127.63, 128.30, 129.31, 130.31, 131.29, 133.91, 134.67, 137.15, 157.90, 160.00, 163.31, 168.48. ESI MS: 486 [M-H]$^+$, $C_{25}H_{21}N_5O_4S$(Calcd): C, 61.59; H, 4.34; N, 14.36; O, 13.13; S, 6.58. Obsd:C,61.57, H, 4.35;N,14.34; O, 13.15;S, 6.60.

2-(5-((S)-1-(2-methoxynaphthalen-6-yl)ethyl)-1,3,4-oxadiazol-2-ylthio)-N-(4-fluorophenyl) acetamide (19)

M.p: 220-221; yield:71%; IR (ν, cm^{-1}): 3195 (N-H), 3050, 2966, 1734, 1640, 1592, 1509, 1483, 1377, 1247, 1225, 1198, 1159, 1032, 939, 882; ^{1}H NMR (850 MHz, $CDCl_3$): δ 1.68 (d, *J* = 8.50 Hz, 3H, CH_3), 3.93-3.97 (m, 5H, -O-CH_3, -S-CH_2-), 4.06 (q, *J* = 8.50 Hz, 1H, -CH), 7.02 (d, *J* = 8.5Hz, 1H, 1 × CH), 7.10 (s, 1H, 1 × CH), 7.15 (d, *J* = 8.5Hz, 2H, 2 × CH), 7.19-7.21 (m, 2H, 2 × CH), 7.42 (d, *J* = 8.50Hz, 1H, 1 × CH), 7.58 (t, *J* = 8.50 Hz, 2H, 2 × CH), 7.72-7.77 (m, 2H, 2× CH), 9.35(s, 1H, N-H). ^{13}C NMR (213 MHz, $CDCl_3$): δ 18.84, 33.05, 42.26, 55.34, 105.56, 105.68, 116.49, 116.60, 118.86, 119.40, 125.86, 126.61, 128.81,129.66, 129.88, 129.93, 133.42, 135.20, 136.21, 157.52, 162.05, 163.21, 169.32, 175.49. ESI MS: 438 [M+H]$^+$, $C_{23}H_{20}N_3O_3S$(Calcd): C, 63.14; H, 4.61; N, 9.60; O, 10.97; S, 7.33. Obsd: C, 63.15, H, 4.60: N,9.59; O, 10.98; S, 7.34

2-(5-((S)-1-(2-methoxynaphthalen-6-yl)ethyl)-1,3,4-oxadiazol-2-ylthio)-N-(2,4-difluorophenyl)acetamide (20)

M.p: 102-103; yield:89%; IR (ν, cm^{-1}): 3150 (N-H), 3087, 2940, 1681, 1606, 1569, 1505, 1483, 1470, 1260, 1144, 1028, 850, 813; ^{1}H NMR (850 MHz, $CDCl_3$): δ 1.84 (d, *J* = 8.50 Hz, 3H, -CH_3), 3.94-4.06 (m, 3H, O-CH_3, 2H, -S-CH_2-), 4.49 (q, *J* = 8.50 Hz, 1H, -CH), 6.95-6.97 (m, 1H, 1 × CH), 7.13 (d, 7.60 Hz, 1H, 1 × CH), 7.17-7.19 (m, 1H, 1 × CH), 7.36-7.37 (m, 1H, 1 × CH), 7.52 (t, J = 8.50 Hz, 1H, 1 × CH), 7.67 (s, 1H, 1 × CH), 7.71-7.73 (m, 2H, 2 × CH), 8.26 (s, 1H, 1 × CH), 9.30 (s, 1H, N-H). ^{13}C NMR (213 MHz, $CDCl_3$): δ 19.25, 29.30, 37.52, 55.37, 105.82, 119.40, 122.77, 125.59, 125.91, 127.89, 128.94, 129.31, 133.94, 134.56, 157.98, 164.38, 165.89, 171.03. ESI MS: 456 [M+H]$^+$, $C_{23}H_{19}N_3O_3S$(Calcd): C, 60.65; H, 4.20; N, 9.23; O, 10.54; S, 7.04. Obsd: C, 60.64, H, 4.21: N,9.24; O, 10.55; S, 7.03

2-(5-((S)-1-(2-methoxynaphthalen-6-yl)ethyl)-1,3,4-oxadiazol-2-ylthio)-N-(3-chlorophenyl) acetamide (21)

M.p: 82-83; yield: 90%; IR (ν, cm^{-1}): 3148, 3050, 2934, 1634, 1593, 1480, 1263, 1158, 1029, 852; ^1H NMR (850 MHz, $CDCl_3$): δ 1.86 (d, *J* = 8.50 Hz, 3H, CH_3), 3.90-3.95 (m, 5H, -O-CH_3, -S-CH_2-), 4.49 (q, *J* = 8.50 Hz, 1H, -C-H), 7.10 (d, *J* = 8.50 Hz, 1H, 1 × CH), 7.13 (d, *J* = 8.5 Hz, 1H, 1 × CH), 7.18 (d, *J* = 8.5 Hz, 1H, 1 × CH), 7.24 (t, *J* = 8.50 Hz, 1 × CH), 7.33 (s, 1H, 1 × CH), 7.38 (d, *J* = 8.5Hz, 1H, 1 × CH), 7.69 (d, *J* = 8.5Hz, 2H, 2 × CH), 7.72-7.75 (m, 2H, 2 × CH), 9.47(s, 1H, N-H).^{13}C NMR (213 MHz, $CDCl_3$): δ 19.21, 26.93, 37.55, 55.37, 105.64, 117.90, 119.44, 119.96, 124.65, 125.59, 126.00, 126.40, 127.78, 129.32, 129.96, 134.00, 134.67, 138.84, 158.00, 162.19, 165.64, 172.79. ESI MS: 454 $[M+H]^+$, 456 $[M+H+2]^+$ $C_{23}H_{20}N_3O_3S$(Calcd): C, 60.86; H, 4.44; N, 9.26; O, 10.57; S, 7.06. Obsd: C,60.85, H, 4.45: N,9.25; O, 10.58; S, 7.05

2-(5-((S)-1-(2-methoxynaphthalen-6-yl)ethyl)-1,3,4-oxadiazol-2-ylthio)-N-(3-bromophenyl) acetamide (22)

M.p: 129-130; yield:90%; IR (ν, cm^{-1}): 3111, 3040, 2933, 1671, 1595, 1539, 1471, 1456, 1263, 1214, 1028, 850; ^1H NMR (850 MHz, $CDCl_3$): δ 1.86 (d, *J* = 8.50 Hz, 3H, -CH_3), 3.92-3.94 (m, 5H, -O-CH_3, -S-CH_2-), 4.49 (q, *J* = 8.50 Hz, 1H, -CH), 7.13 (d, J = 8.5Hz, 1H, 1 × CH), 7.17-7.19 (m, 2H, 2 × CH), 7.25 (d, *J* = 8.5Hz, 1H, 1 × CH), 7.38 (d, *J* = 8.5Hz, 1H, 1 × CH), 7.43 (d, *J* = 8.5Hz, 1H, 1 × CH), 7.69 (s, 1H, 1 × CH), 7.72-7.75 (m, 2H, 2 × CH), 7.84 (s, 1H, 1 × CH), 9.45 (s, 1H, -N-H).^{13}C NMR (213 MHz, $CDCl_3$): δ 19.22, 26.14, 37.56, 55.37, 105.64, 118.40, 119.45, 122.65, 122.77, 125.60, 126.00, 127.60, 127.76, 128.87, 129.32, 130.26, 134.01, 134.38, 138.97, 159.45, 161.71, 163.15, 175.43. ESI MS: 498 $[M+H]^+$, 500 $[M+H+2]^+$ $C_{23}H_{20}N_3O_3S$(Calcd): C, 55.43; H, 4.04; N, 8.43; O, 9.63; S, 6.43. Obsd: C, 55.42, H, 4.06: N,8.42; O, 9.62; S, 6.45

2-(5-((S)-1-(2-methoxynaphthalen-6-yl)ethyl)-1,3,4-oxadiazol-2-ylthio)-N-(4-bromophenyl) acetamide (23)

M.p: 148-149; yield:71%; IR (ν, cm^{-1}):3101, 3041, 2935, 1675, 1525, 1405, 1263, 1025, 855; ^1H NMR (850 MHz, $CDCl_3$): δ 1.86 (d, *J* = 7.60 Hz, 3H, -CH_3), 3.92-3.94 (m, 5H, O-CH_3, S-CH_2-), 4.48 (q, *J* = 8.50 Hz, 1H, -CH), 7.13 (d, *J* = 8.50, 1H, 1 × CH), 7.37 (d, *J* = 8.5Hz, 1H, 1 × CH), 7.42 (d, *J*= 8.5Hz, 2H, 2 × CH), 7.45 (d, *J* = 8.5Hz, 2H, 2 × CH), 7.59 (t, *J* = 8.5Hz, 1H, 1 × CH), 7.68 (s, 1H, 1 × CH), 7.71-7.75 (m, 2H, 1 × CH), 9.45 (s, 1H, N-H). ^{13}C NMR (213 MHz, $CDCl_3$): δ 19.22, 30.96, 37.54, 55.37, 105.64, 1117.18, 119.45, 121.43, 123.26, 125.58, 125.99, 126.89, 127.76, 128.86, 129.31, 129.62, 131.94, 132.72, 134.00, 134.36, 136.81, 158.01, 163.26, 165.51,

171.04. ESI MS: 498 $[M+H]^+$, 500 $[M+H+2]^+$ $C_{23}H_{20}N_3O_3S$(Calcd): C, 55.43; H, 4.04; N, 8.43; O, 9.63; S, 6.43. Obsd: C, 55.44, H, 4.03: N,8.44; O, 9.64; S, 6.45

2-(5-((S)-1-(2-methoxynaphthalen-6-yl)ethyl)-1,3,4-oxadiazol-2-ylthio)-N-o-tolylacetamide (24)

M.p: 109-110; yield:75%; IR (ν, cm^{-1}): 3315, 2931, 1672, 1596, 1549, 1485, 1470, 1456, 1371, 1251, 1214, 1159, 1028, 850; ^1H NMR (850 MHz, $CDCl_3$): δ 1.85 (d, *J* = 7.60 Hz, 3H, -CH-**C<u>H</u>**$_3$), 2.27 (s, 3H, Ar-CH_3), 3.94 (s, 3H, O-CH_3), 4.0 (brs, 2H, -S-CH_2-), 4.49 (q, *J* = 8.5Hz, 1H, C-H), 7.08 (t, *J* = 8.5Hz, 1H, 1 × CH), 7.13 (s, 1H, 1 × CH), 7.17-7.22 (m, 3H, 3 × CH), 7.36 (d, *J* = 8.5Hz, 1H, 1 × CH), 7.68 (s, 1H, 1 × CH), 7.71-7.74 (m, 2H, 2 × CH), 7.90 (d, *J* = 8.5Hz, 1H, 1 × CH), 8.91 (s, 1H, -N-H). ^{13}C NMR (213 MHz, $CDCl_3$): δ 18.04, 19.24, 26.93, 37.51, 55.36, 105.64, 119.41, 122.46, 125.24, 125.58, 125.93, 126.67, 127.73, 128.88, 129.05, 129.32, 130.55, 133.97, 135.71, 158.25, 160.55, 163.01, 176.59. ESI MS: 434 $[M+H]^+$, $C_{24}H_{23}N_3O_3S$(Calcd): C, 66.49; H, 5.35; N, 9.69; O, 11.07; S, 7.40. Obsd: C, 66.50, H, 5.34, N,9.68; O, 11.09; S, 7.41

2-(2-(5-((S)-1-(2-methoxynaphthalen-6-yl)ethyl)-1,3,4-oxadiazol-2-ylthio)acetamido)benzoic acid (25)

M.p: 130-131; yield:68%; IR (ν, cm^{-1}): 3150 , 2976, 1690, 1684, 1605, 1576, 1483, 1470, 1393, 1262, 1228, 1214, 1152, 1028, 852, 767; ^1H NMR (850 MHz, $CDCl_3$): δ 1.72 (d, *J* = 8.50 Hz, 3H, CH_3), 3.92-3.94 (m, 5H, O-CH_3, S-CH_2-), 4.02-4.07 (m, 1H, -CH), 7.11-7.19 (m, 3H, 3 × CH), 7.42-7.54 (m, 3H, 3 × CH), 7.72-7.81 (m, 3H, 3 × CH), 7.84 (s, 1H, 1 × CH), 8.58 (s. 1H, NH), 10.71 (s, 1H, -COOH). ^{13}C NMR (213 MHz, $CDCl_3$,): δ 18.07, 33.93, 45.65, 55.38, 105.73, 119.52, 126.31, 127.00, 127.93, 129.03, 129.32, 133.99, 134.12, 135.64, 145.83, 158.04, 158.76, 160.18, 169.76, 174.15. ESI MS: 464 $[M+H]^+$, $C_{24}H_{21}N_3O_5S$(Calcd): C, 62.19; H, 4.57; N, 9.07; O, 17.26; S, 6.92. Obsd: C, 62.20, H, 4.56, N, 9.08; O, 17.25; S, 6.93

2-(5-((S)-1-(2-methoxynaphthalen-6-yl)ethyl)-1,3,4-oxadiazol-2-ylthio)-1-morpholino ethanone (26)

M.p: 74-75; yield:70%; IR (ν, cm^{-1}): 3050, 2936, 1633, 1605, 1481, 1457, 1392, 1263, 1228, 1149, 1068, 1027, 850, 812; ^1H NMR (850 MHz, $CDCl_3$): δ 1.83 (d, *J* = 8.5 Hz, 3H, -CH_3), 3.59 (t, *J* = 8.5Hz, 2H, -N-CH_2-), 3,66 (t, *J* = 8.5Hz, 2H, N-CH_2-), 3.70 (t, *J* = 8.5Hz, 2H, O-CH_2-), 3.73 (t, *J* = 8.5Hz, 2H, O-CH_2-), 3.94 (brs, 5H, O-CH_3, S-CH_2-), 4.48 (q, *J* = 8.5Hz, 1H, -CH), 7.14-7.19 (m, 3H, 3 × CH), 7.36-

7.39 (m, 1H, 1 × CH), 7.68 (s, 1H, 1 × CH), 7.72-7.75 (m, 1H, 1 × CH). ^{13}C NMR (213 MHz, $CDCl_3$,): δ 19.35, 37.69, 42.63, 46.54, 55.36, 66.46, 105.64, 119.32, 119.45, 125.55, 125.74, 127.62, 127.76, 129.34, 133.91, 134.85, 157.91, 162.83, 164.38, 174.52. ESI MS: 414 $[M+H]^+$, $C_{21}H_{23}N_3O_4S$(Calcd): C, 61.00; H, 5.61; N, 10.16; O, 15.48; S, 7.75. Obsd:C, 60.01, H, 5.60: N, 10.17; O, 15.50; S, 7.73

Table S1. The calculated GCRD derived from frontier molecular orbitals for **8-11** and **19-26**

	E_{HOMO}	E_{LUMO}	E_{gap}	IP	EA	η	μ	S	χ	ω	$ω^{\pm}$
**8**	-5.83	-1.89	-3.94	5.83	1.89	1.97	-4.85	0.51	3.79	-3.86	9.507
**9**	-5.71	-1.89	-3.82	5.71	1.89	1.91	-4.75	0.52	3.78	-3.80	9.450
**10**	-5.69	-1.82	-3.87	5.69	1.82	1.94	-4.73	0.52	3.65	-3.76	9.175
**11**	-5.67	-2.04	-3.64	5.67	2.04	1.82	-4.76	0.55	4.08	-3.86	10.097
**12**	-5.72	-2.00	-3.72	5.72	2.00	1.86	-4.79	0.54	4.01	-3.86	9.959
**13**	-5.67	-2.06	-3.61	5.67	2.06	1.80	-4.77	0.55	4.14	-3.86	10.207
**14**	-5.82	-2.03	-3.79	5.82	2.03	1.90	-4.88	0.53	4.07	-3.93	10.105
**15**	-6.11	-1.80	-4.31	6.11	1.80	2.16	-5.03	0.46	3.63	-3.96	9.234
**16**	-1.87	-0.58	-1.29	1.87	0.58	0.64	-1.55	1.55	1.16	-1.22	2.940
**19**	-5.90	-1.52	-4.38	5.90	1.52	2.19	-4.81	0.46	3.15	-3.71	8.154
**20**	-5.77	-1.61	-4.15	5.77	1.61	2.08	-4.73	0.48	3.28	-3.69	8.400
**21**	-6.07	-1.70	-4.38	6.07	1.70	2.19	-4.98	0.46	3.45	-3.89	8.841
**22**	-5.92	-1.76	-4.16	5.92	1.76	2.08	-4.88	0.48	3.55	-3.84	9.015
**23**	-5.89	-1.51	-4.38	5.89	1.51	5.13	-4.79	-0.21	0.01	3.32	9.958
**24**	-5.88	-1.53	-4.35	5.88	1.53	2.18	-4.79	0.46	3.15	-3.70	8.148
**25**	-1.57	-0.20	-1.37	1.57	0.20	0.68	-1.23	1.46	0.57	-0.89	1.592
**26**	-5.70	-1.34	-4.36	5.70	1.34	2.18	-4.61	0.46	2.84	-3.52	7.445

Figure S1-S17: ^{1}H NMR of compounds

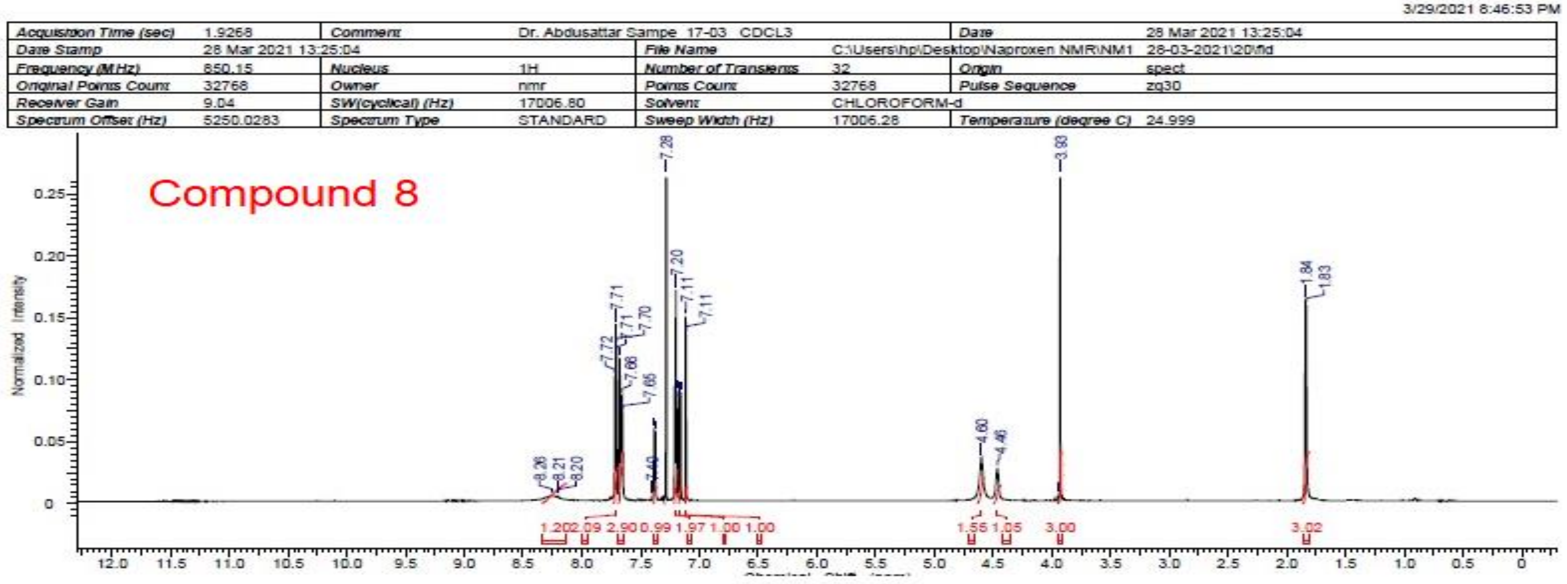

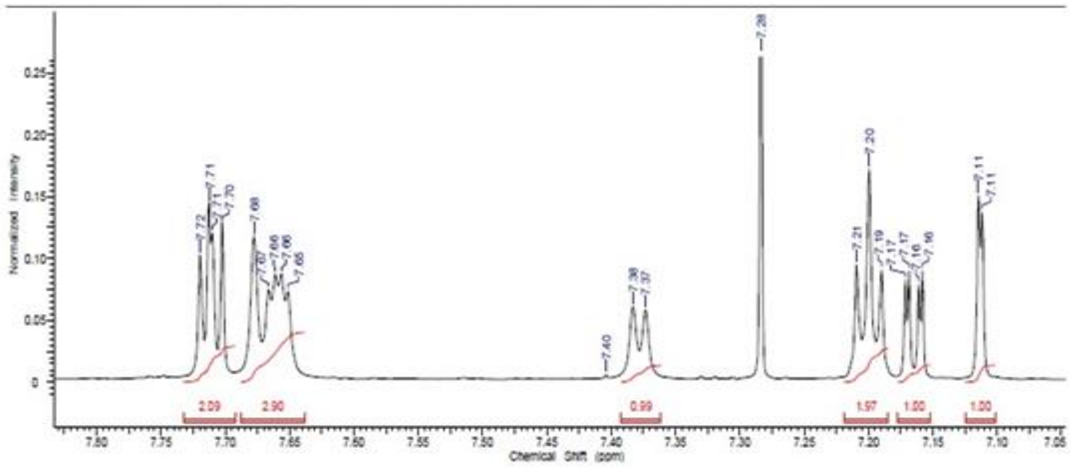

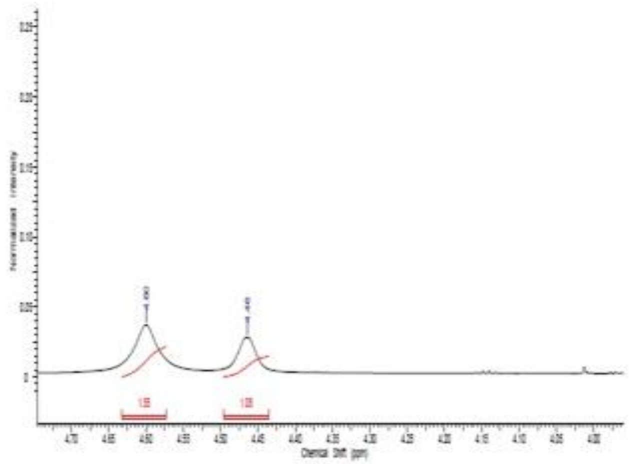

3/29/2021 11:45:17 PM

Acquisition Time (sec)	1.9268	Comment	Dr. Abdusattar Sampe 20-03 CDCL3			Date	28 Mar 2021 16:05:04
Date Stamp	28 Mar 2021 16:05:04			File Name	C:\Users\hp\Desktop\Naproxen NMR\NM4 28-03-2021\50\fid		
Frequency (MHz)	850.15	Nucleus	1H	Number of Transients	32	Origin	spect
Original Points Count	32768	Owner	nmr	Points Count	32768	Pulse Sequence	zg30
Receiver Gain	12.45	SW(cyclical) (Hz)	17006.80	Solvent	CHLOROFORM-d		
Spectrum Offset (Hz)	5250.0283	Spectrum Type	STANDARD	Sweep Width (Hz)	17006.28	Temperature (degree C)	24.999

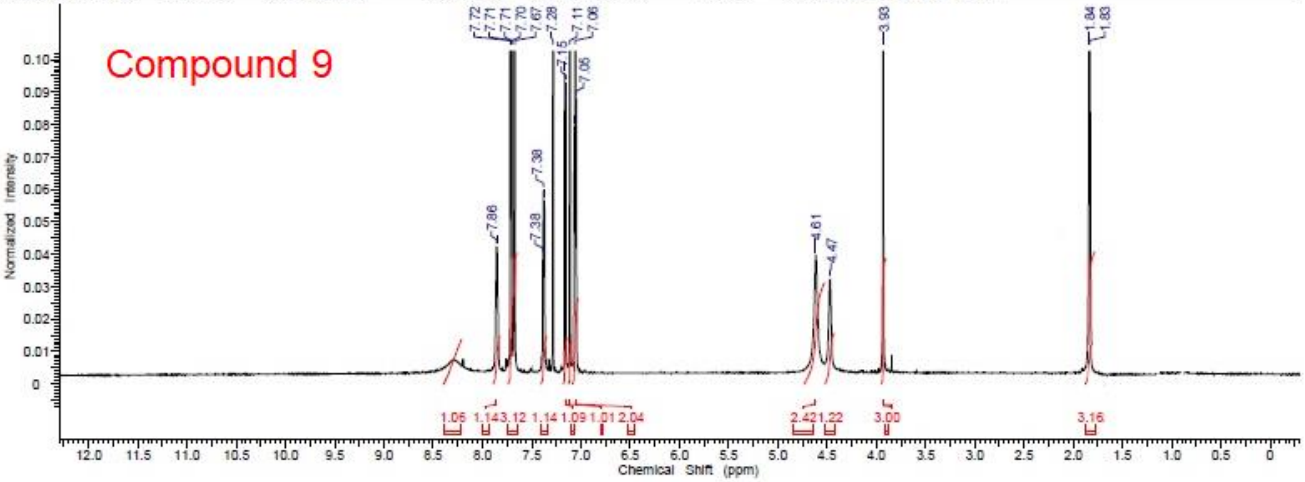

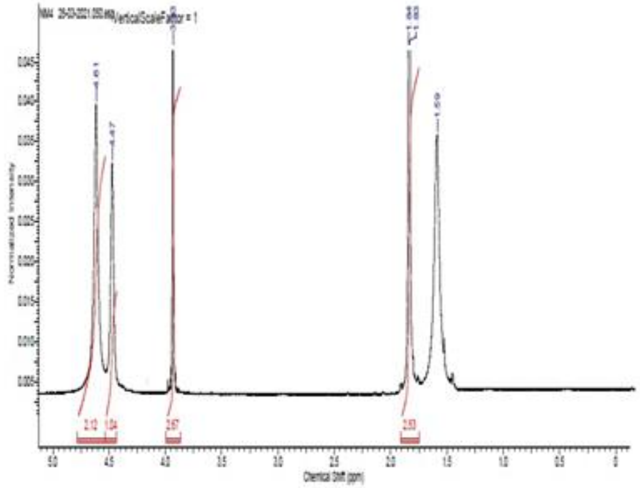

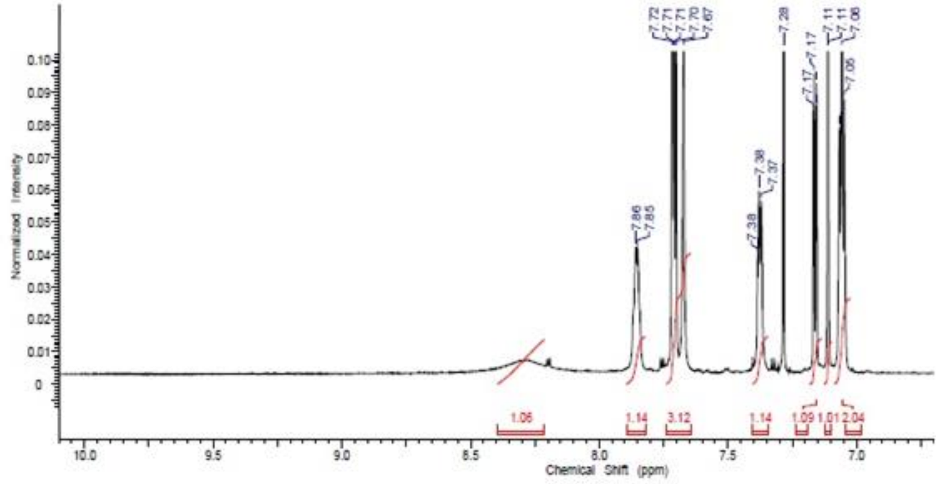

3/30/2021 1:33:23 AM

Acquisition Time (sec)	1.9268	Comment	Dr. Abdusattar Sampe 23-03 CDCL3			Date	28 Mar 2021 18:45:04
Date Stamp	28 Mar 2021 18:45:04			File Name	C:\Users\hp\Desktop\Naproxen NMR\NM7 28-03-2021\80\fid		
Frequency (MHz)	850.15	Nucleus	1H	Number of Transients	32	Origin	spect
Original Points Count	32768	Owner	nmr	Points Count	32768	Pulse Sequence	zg30
Receiver Gain	12.46	SW(cyclical) (Hz)	17006.80	Solvent	CHLOROFORM-d		
Spectrum Offset (Hz)	5250.0283	Spectrum Type	STANDARD	Sweep Width (Hz)	17006.28	Temperature (degree C)	25.001

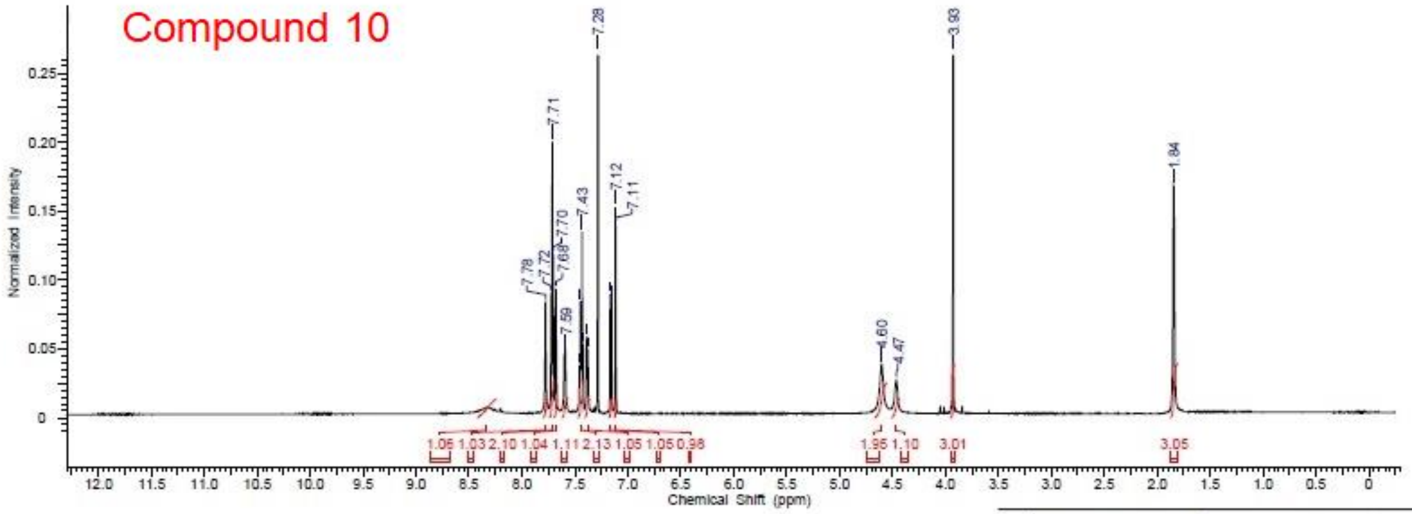

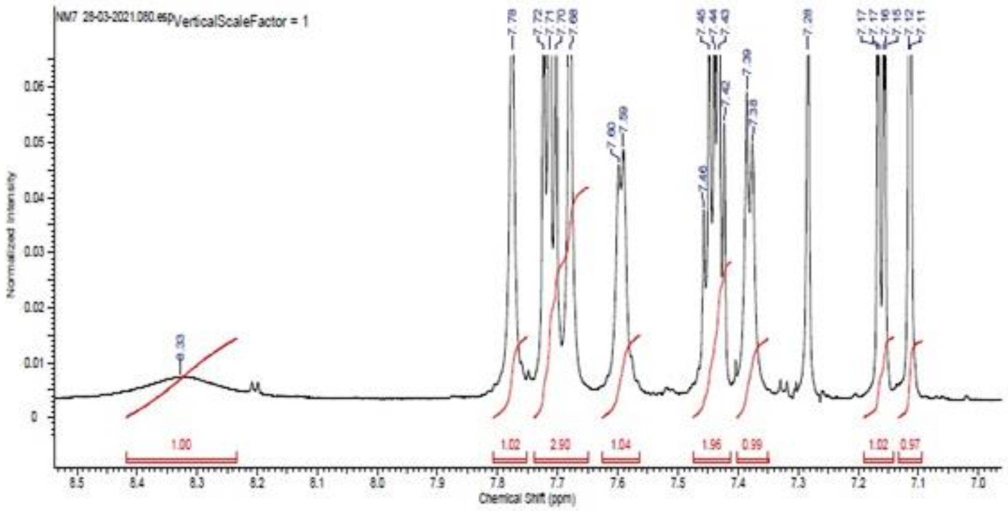

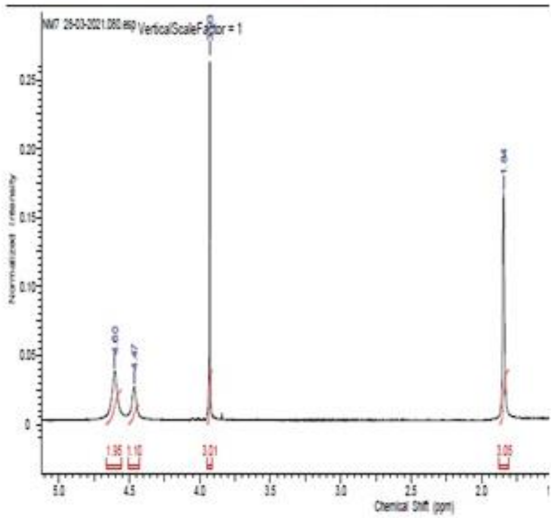

3/30/2021 1:57:36 AM

Acquisition Time (sec)	1.9268	Comment	Dr. Abdusattar Sampe 25-03 CDCL3			Date	28 Mar 2021 20:29:36
Date Stamp	28 Mar 2021 20:29:36			File Name	C:\Users\hp\Desktop\Naproxen NMR\NM9 28-03-2021\100\fid		
Frequency (MHz)	850.15	Nucleus	1H	Number of Transients	32	Origin	spect
Original Points Count	32768	Owner	nmr	Points Count	32768	Pulse Sequence	zg30
Receiver Gain	10.55	SW(cyclical) (Hz)	17006.80	Solvent	CHLOROFORM-d		
Spectrum Offset (Hz)	5250.0283	Spectrum Type	STANDARD	Sweep Width (Hz)	17006.28	Temperature (degree C)	25.000

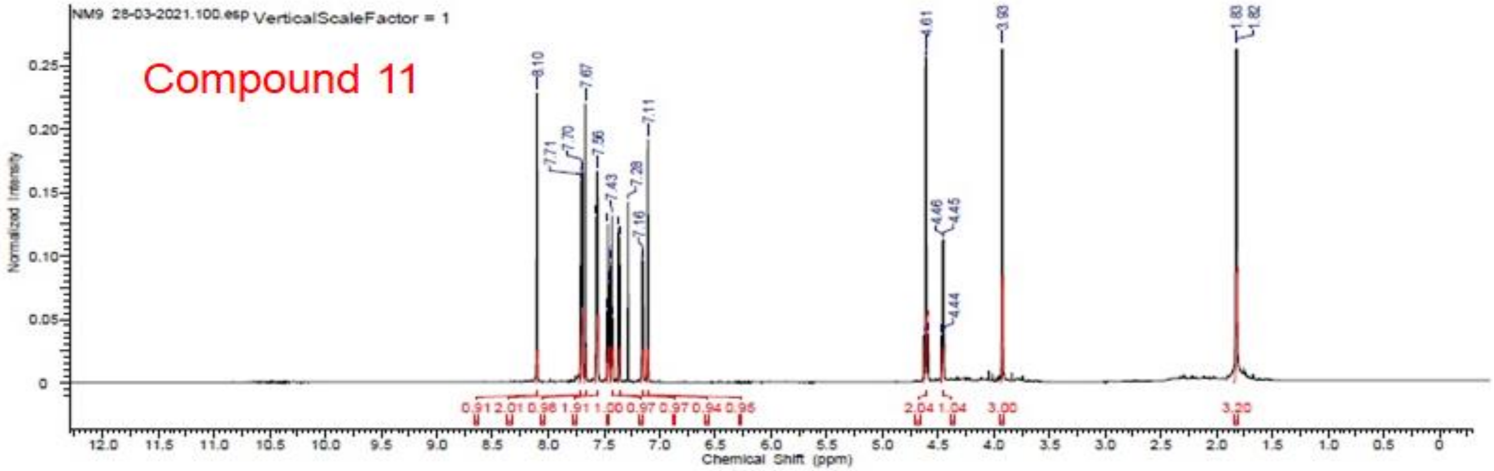

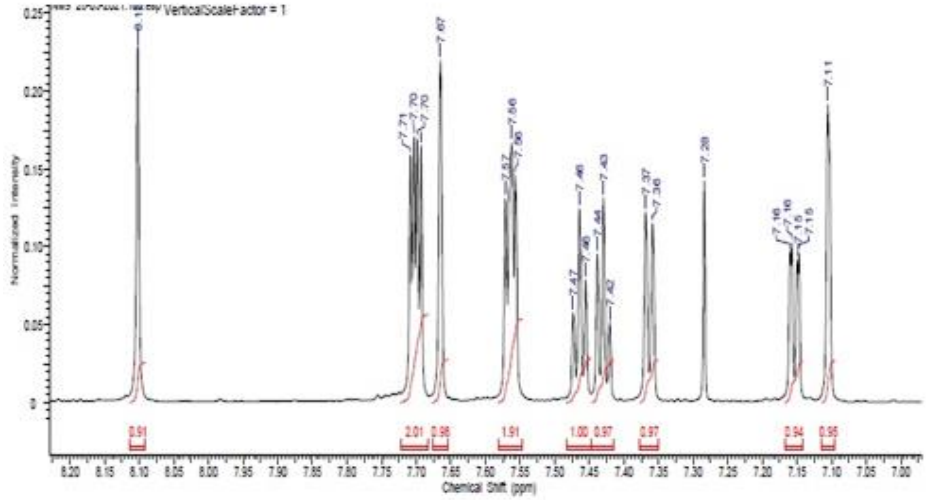

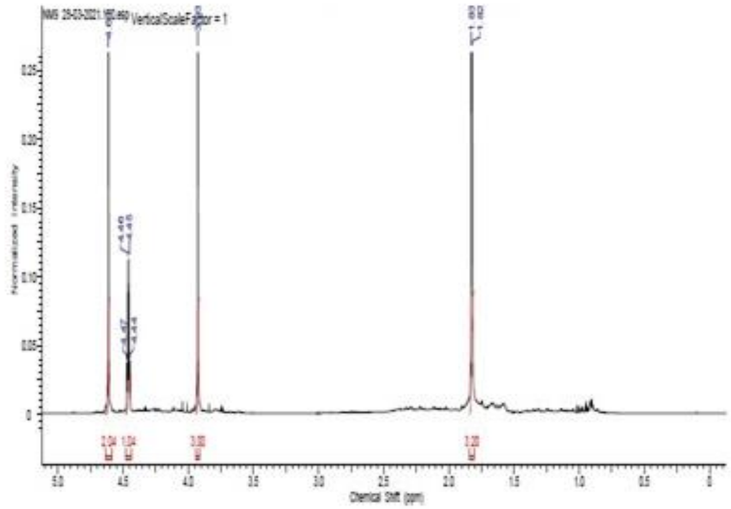

3/29/2021 9:55:14 PM

Acquisition Time (sec)	1.9268	Comment	Dr. Abdusattar Sampe 18-03 CDCL3			Date	28 Mar 2021 14:18:24
Date Stamp	28 Mar 2021 14:18:24			File Name	C:\Users\hp\Desktop\Naproxen NMR\NM2 28-03-2021\30\fid		
Frequency (MHz)	850.15	Nucleus	1H	Number of Transients	32	Origin	spect
Original Points Count	32768	Owner	nmr	Points Count	32768	Pulse Sequence	zg30
Receiver Gain	11.37	SW(cyclical) (Hz)	17006.80	Solvent	CHLOROFORM-d		
Spectrum Offset (Hz)	5250.0283	Spectrum Type	STANDARD	Sweep Width (Hz)	17006.28	Temperature (degree C)	25.001

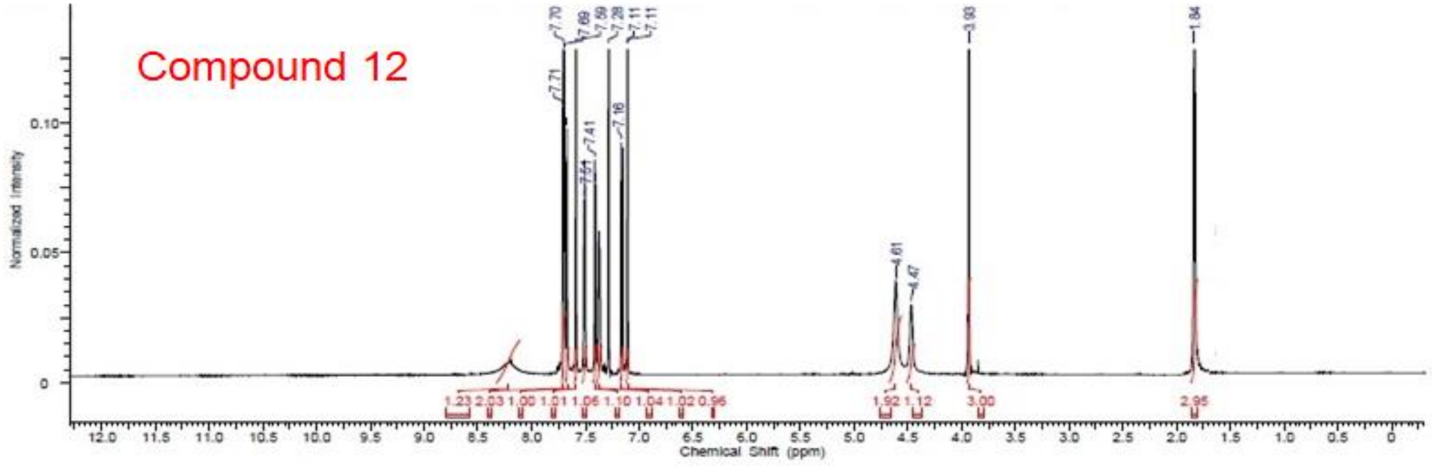

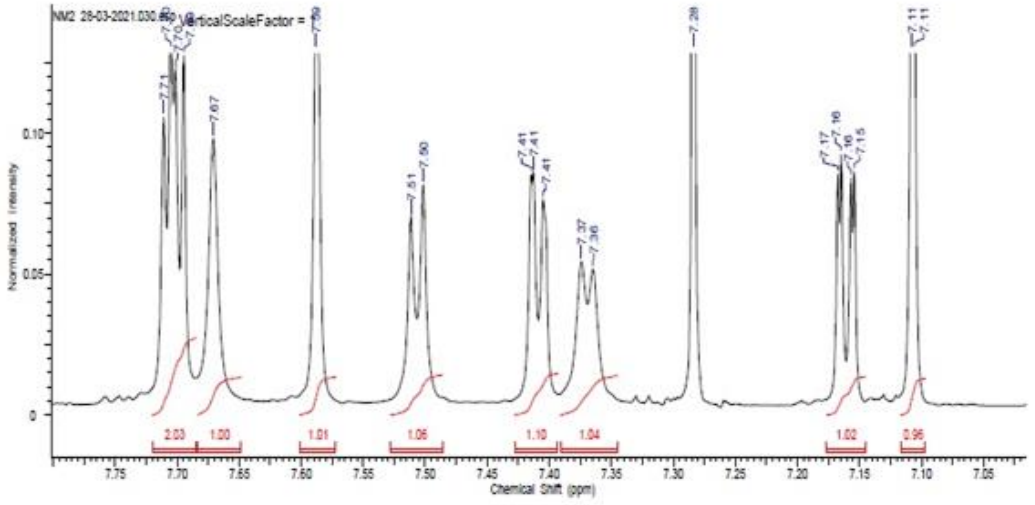

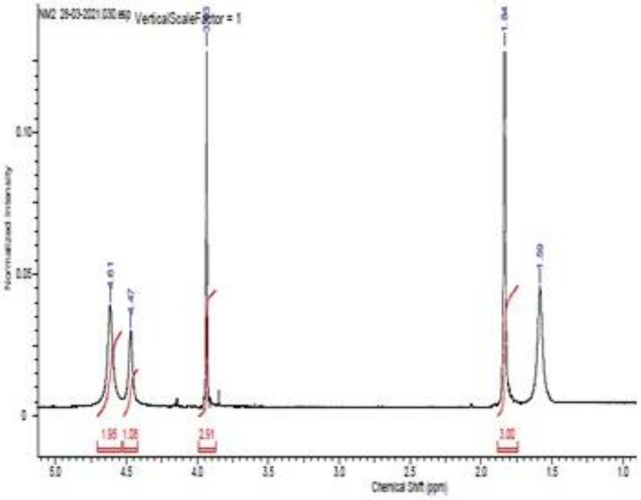

4/30/2021 1:53:27 PM

Acquisition Time (sec)	1.9268	*Comment*	Dr. Abdusattar Sampe 24-03 CDCL3	*Date*	28 Mar 2021 19:38:24
Date Stamp	28 Mar 2021 19:38:24	*File Name*	C:\Users\hp\Desktop\Naproxen NMR\NM8 28-03-2021\90\fid		
Frequency (MHz)	850.15	*Nucleus*	1H	*Number of Transients*	32
Origin	spect				
Original Points Count	32768	*Owner*	nmr	*Points Count*	32768
Pulse Sequence	zg30				
Receiver Gain	11.37	*SW(cyclical) (Hz)*	17006.80	*Solvent*	CHLOROFORM-d
Spectrum Offset (Hz)	5250.0283	*Spectrum Type*	STANDARD	*Sweep Width (Hz)*	17006.28
Temperature (degree C)	25.001				

Compound 13

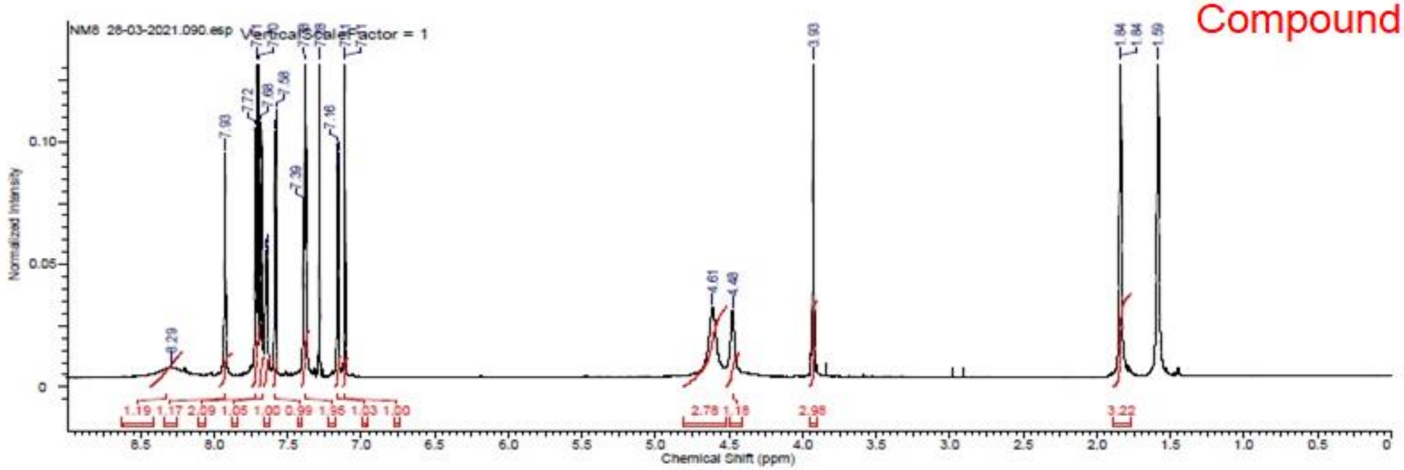

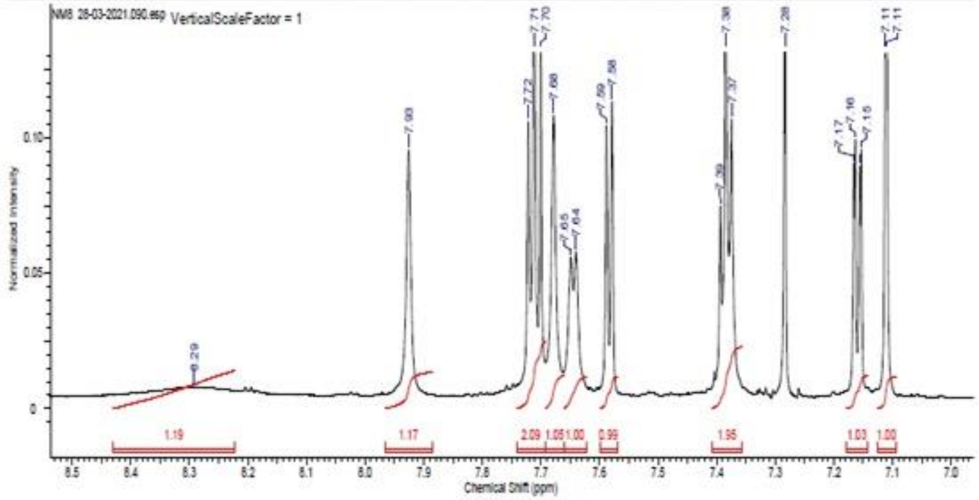

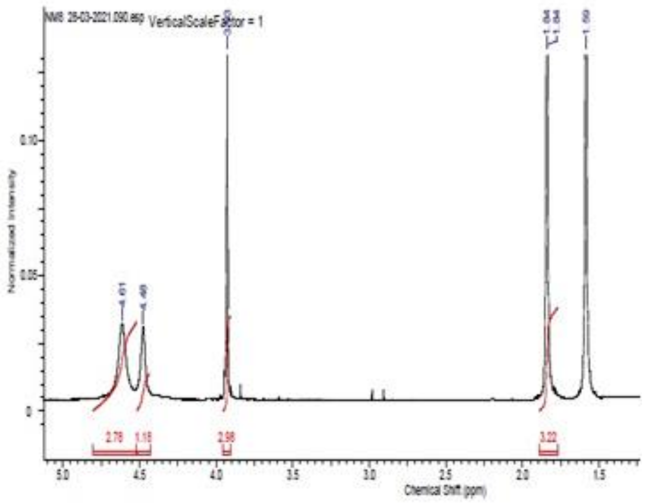

4/30/2021 1:43:48 AM

Acquisition Time (sec)	1.9268	*Comment*	Dr. Abdusattar Sampe 21-03 CDCL3			*Date*	28 Mar 2021 16:56:16
Date Stamp	28 Mar 2021 16:56:16			*File Name*	C:\Users\hp\Desktop\Naproxen NMR\NM5 28-03-2021\60\fid		
Frequency (MHz)	850.15	*Nucleus*	1H	*Number of Transients*	32	*Origin*	spect
Original Points Count	32768	*Owner*	nmr	*Points Count*	32768	*Pulse Sequence*	zg30
Receiver Gain	12.46	*SW(cyclical) (Hz)*	17006.80	*Solvent*	CHLOROFORM-d		
Spectrum Offset (Hz)	5171.6201	*Spectrum Type*	STANDARD	*Sweep Width (Hz)*	17006.28	*Temperature (degree C)*	25.001

Compound 14

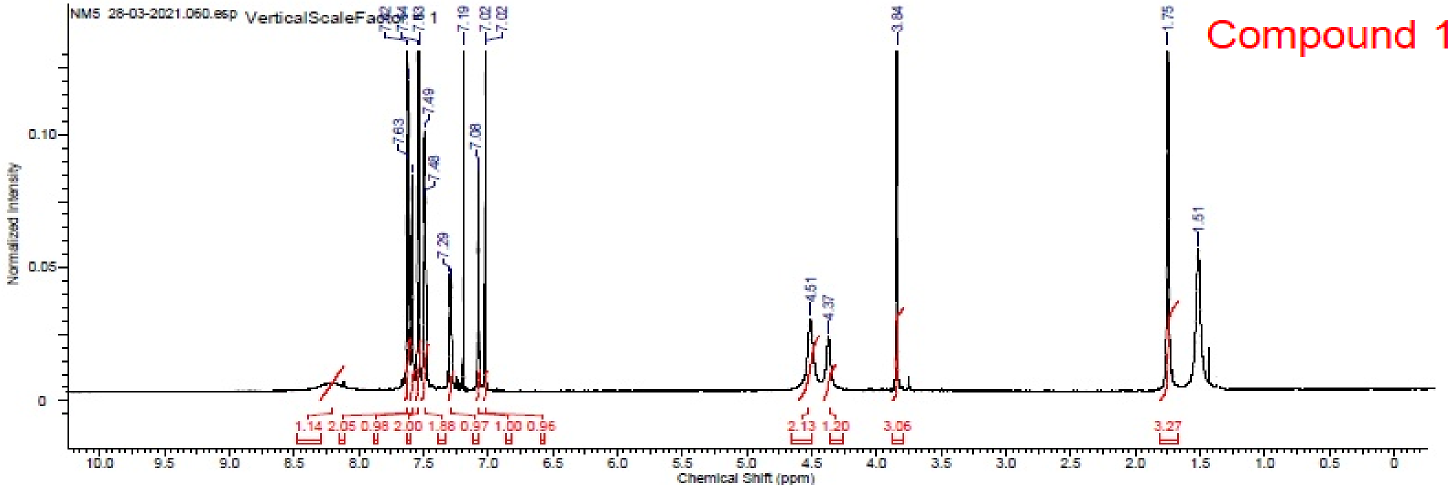

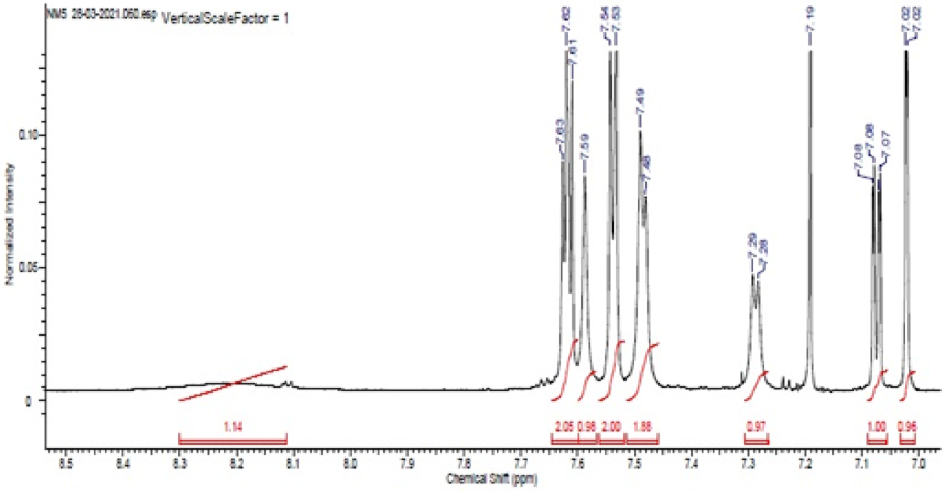

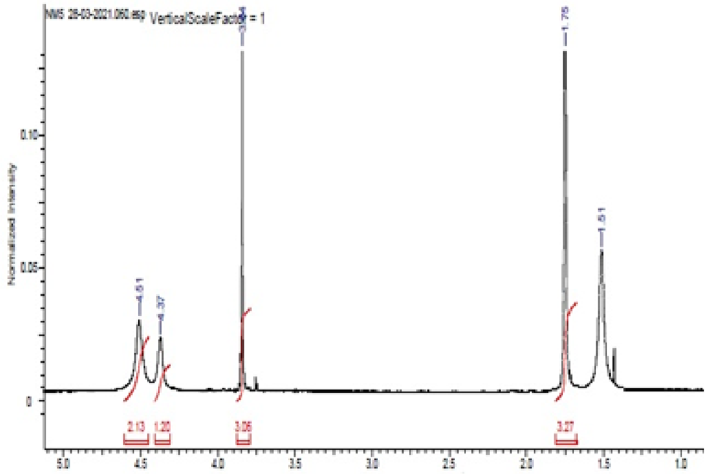

4/30/2021 1:45:14 PM

Acquisition Time (sec)	1.9268	Comment	Dr. Abdusattar Sampe 22-03 CDCL3			Date	28 Mar 2021 17:51:44
Date Stamp	28 Mar 2021 17:51:44			File Name	C:\Users\hp\Desktop\Naproxen NMR\NM6 28-03-2021\70\fid		
Frequency (MHz)	850.15	Nucleus	1H	Number of Transients	32	Origin	spect
Original Points Count	32768	Owner	nmr	Points Count	32768	Pulse Sequence	zg30
Receiver Gain	11.37	SW(cyclical) (Hz)	17006.80	Solvent	CHLOROFORM-d		
Spectrum Offset (Hz)	5250.0283	Spectrum Type	STANDARD	Sweep Width (Hz)	17006.28	Temperature (degree C)	25.000

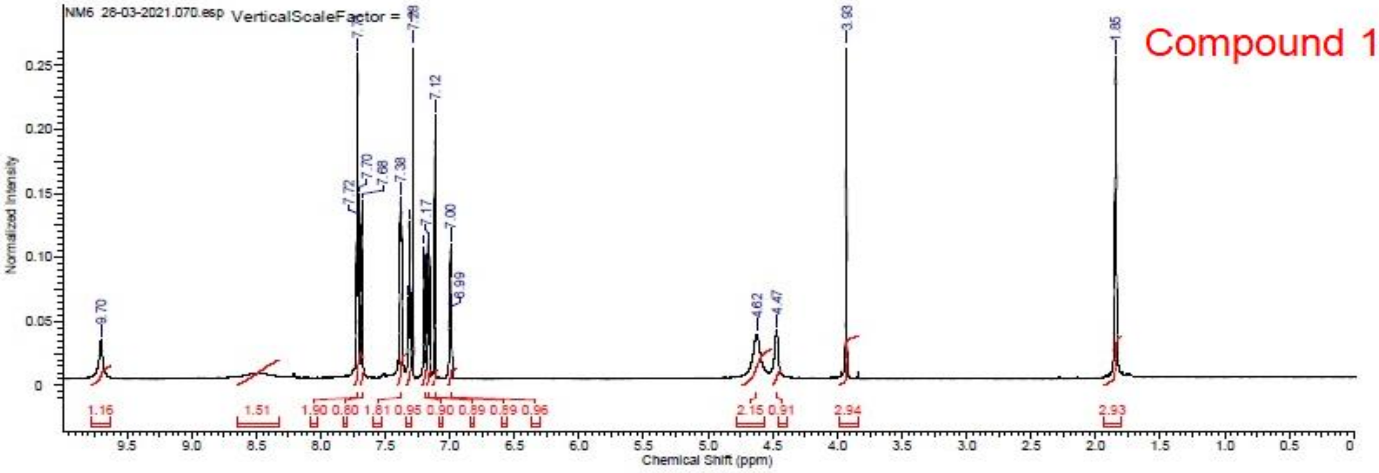

Compound 15

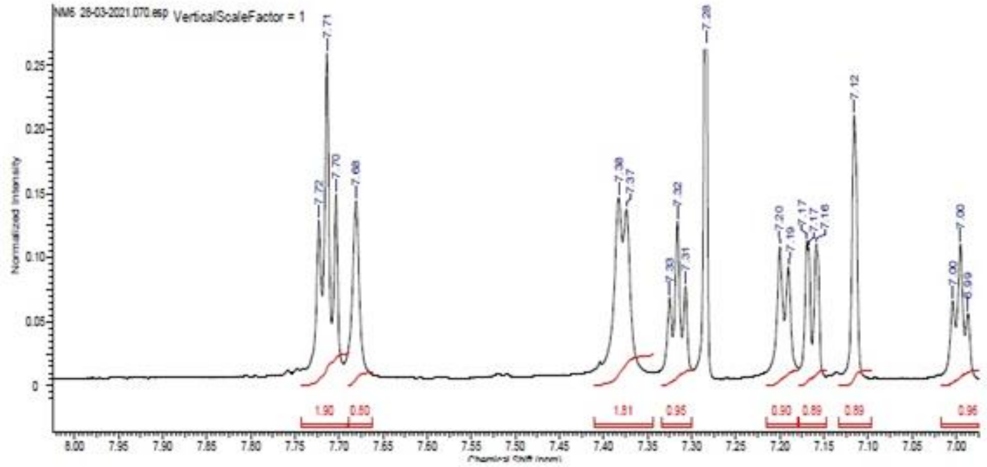

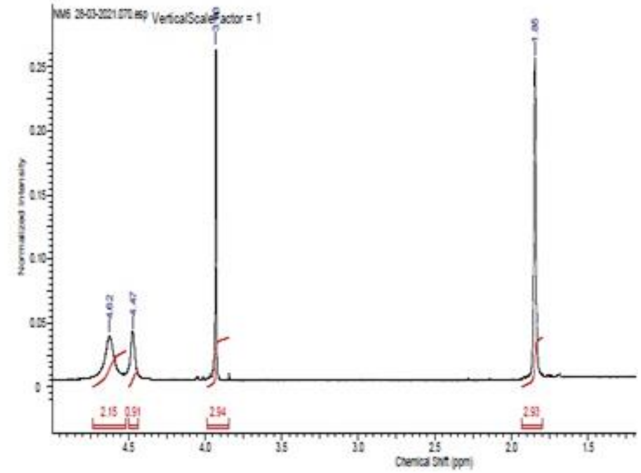

Compound 16

3/29/2021 10:14:06 PM

Acquisition Time (sec)	1.9268	Comment	Dr. Abdusattar Sampe 19-03 CDCL3			Date	28 Mar 2021 15:11:44
Date Stamp	28 Mar 2021 15:11:44			File Name	C:\Users\hp\Desktop\Naproxen NMR\NM3 28-03-2021\40\fid		
Frequency (MHz)	850.15	Nucleus	1H	Number of Transients	32	Origin	spect
Original Points Count	32768	Owner	nmr	Points Count	32768	Pulse Sequence	zg30
Receiver Gain	12.46	SW(cyclical) (Hz)	17006.80	Solvent	CHLOROFORM-d		
Spectrum Offset (Hz)	5250.0283	Spectrum Type	STANDARD	Sweep Width (Hz)	17006.28	Temperature (degree C)	25.000

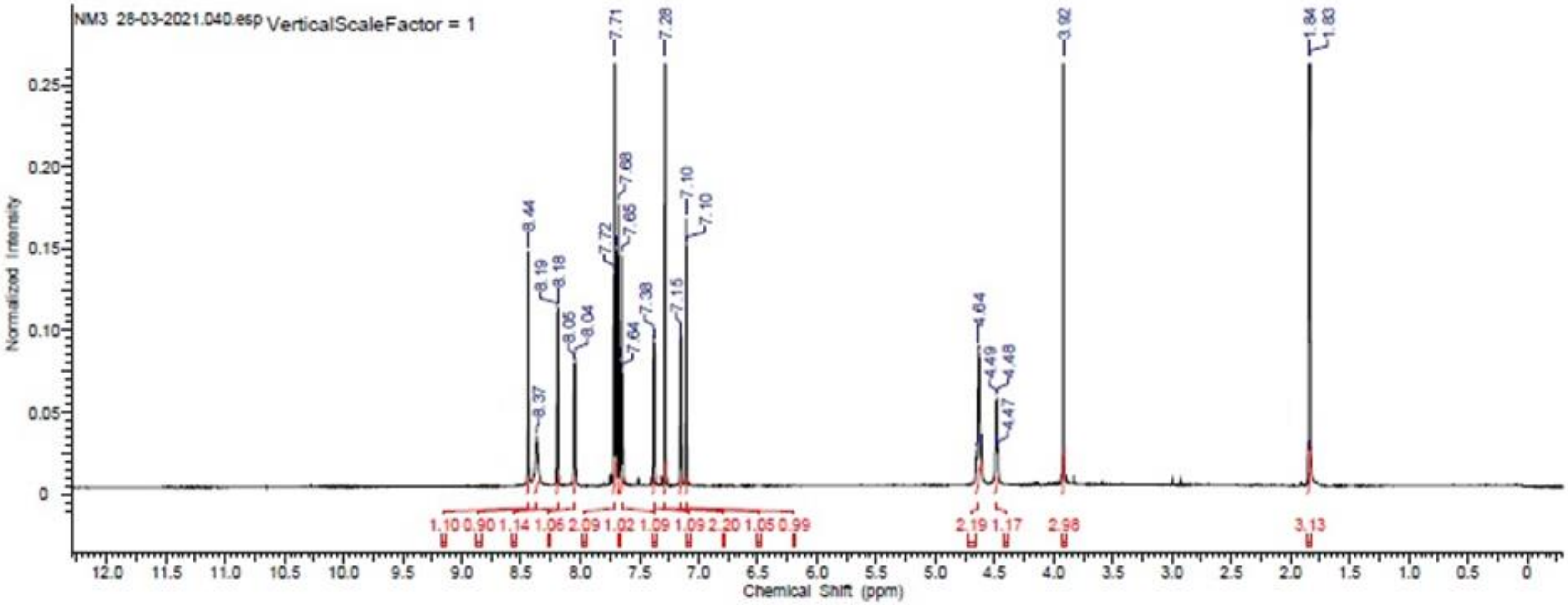

4/30/2021 2:40:13 PM

Acquisition Time (sec)	1.9268	*Comment*	Dr. Abdusattar Sampe 26-03 CDCL3			*Date*	28 Mar 2021 21:22:56
Date Stamp	28 Mar 2021 21:22:56			*File Name*	C:\Users\hp\Desktop\Naproxen NMR\NM10 28-03-2021\110\fid		
Frequency (MHz)	850.15	*Nucleus*	1H	*Number of Transients*	32	*Origin*	spect
Original Points Count	32768	*Owner*	nmr	*Points Count*	32768	*Pulse Sequence*	zg30
Receiver Gain	11.37	*SW(cyclical) (Hz)*	17006.80	*Solvent*	CHLOROFORM-d		
Spectrum Offset (Hz)	5250.0283	*Spectrum Type*	STANDARD	*Sweep Width (Hz)*	17006.28	*Temperature (degree C)*	25.000

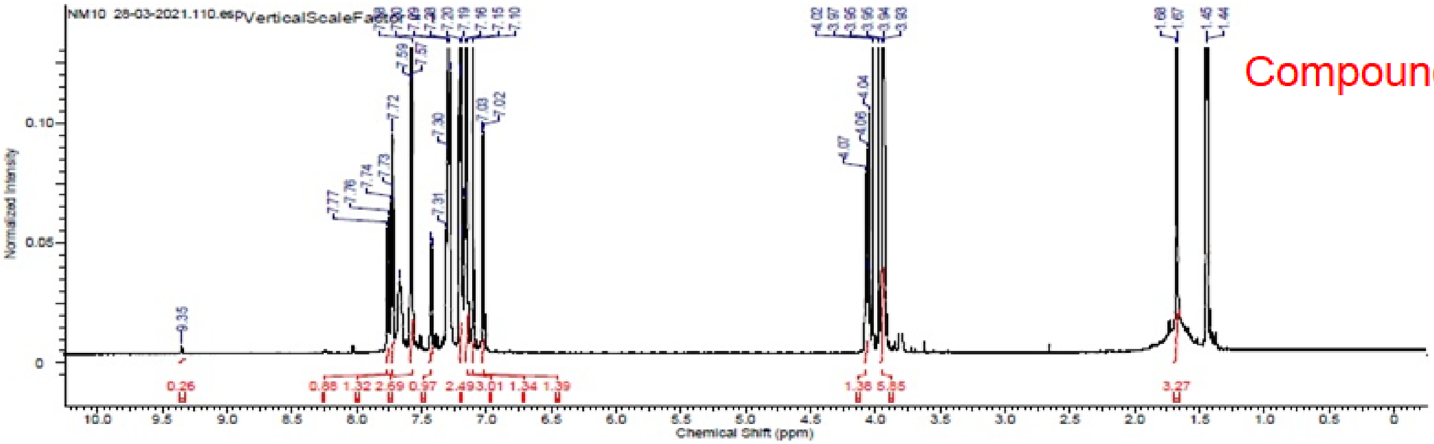

Compound 19

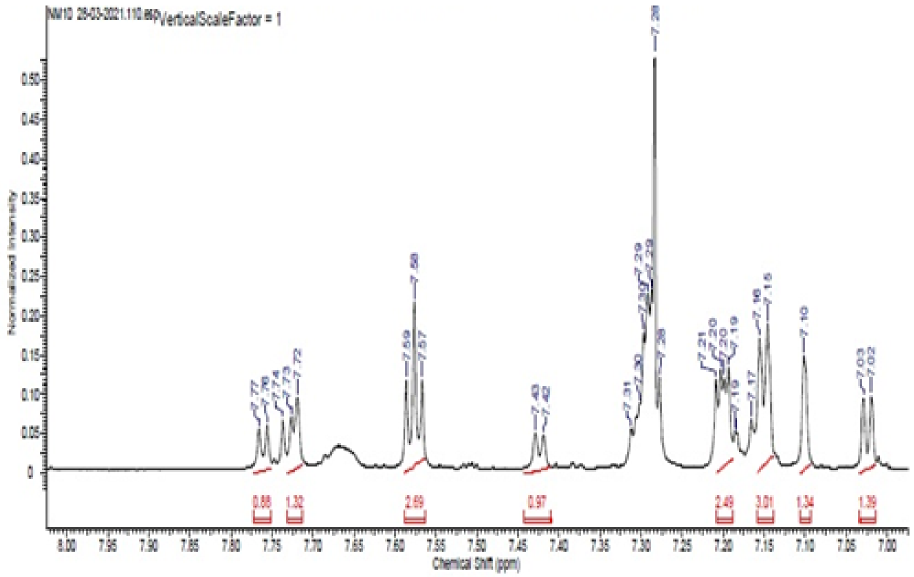

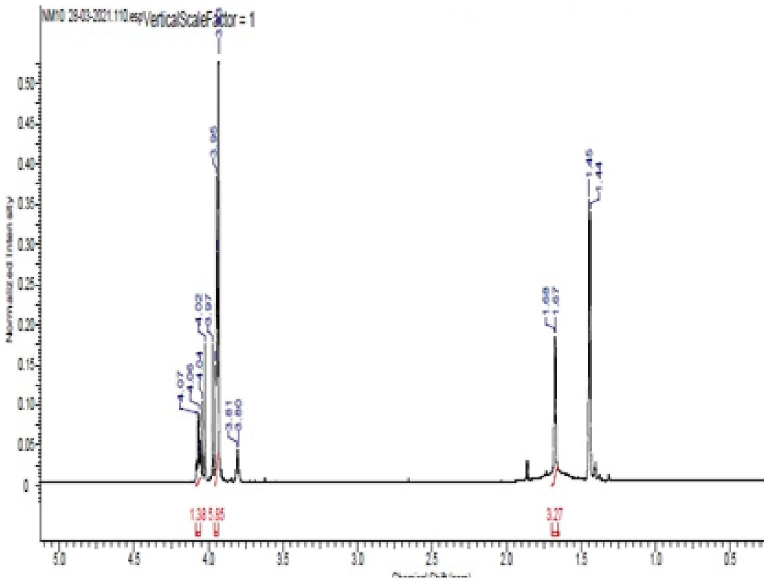

4/30/2021 2:51:21 PM

Acquisition Time (sec)	1.9268	*Comment*	Dr. Abdusattar Sampe 27-03 CDCL3			*Date*	28 Mar 2021 22:16:16
Date Stamp	28 Mar 2021 22:16:16			*File Name*	C:\Users\hp\Desktop\Naproxen NMR\NM11 28-03-2021\120\fid		
Frequency (MHz)	850.15	*Nucleus*	1H	*Number of Transients*	32	*Origin*	spect
Original Points Count	32768	*Owner*	nmr	*Points Count*	32768	*Pulse Sequence*	zg30
Receiver Gain	11.37	*SW(cyclical) (Hz)*	17006.80	*Solvent*	CHLOROFORM-d		
Spectrum Offset (Hz)	5250.0283	*Spectrum Type*	STANDARD	*Sweep Width (Hz)*	17006.28	*Temperature (degree C)*	24.998

Compound 20

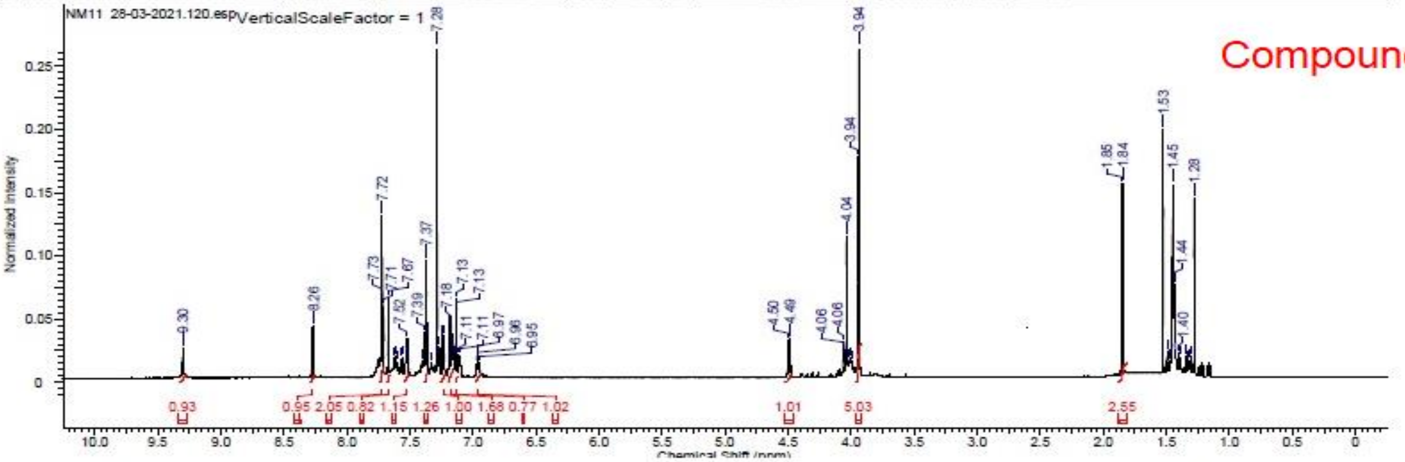

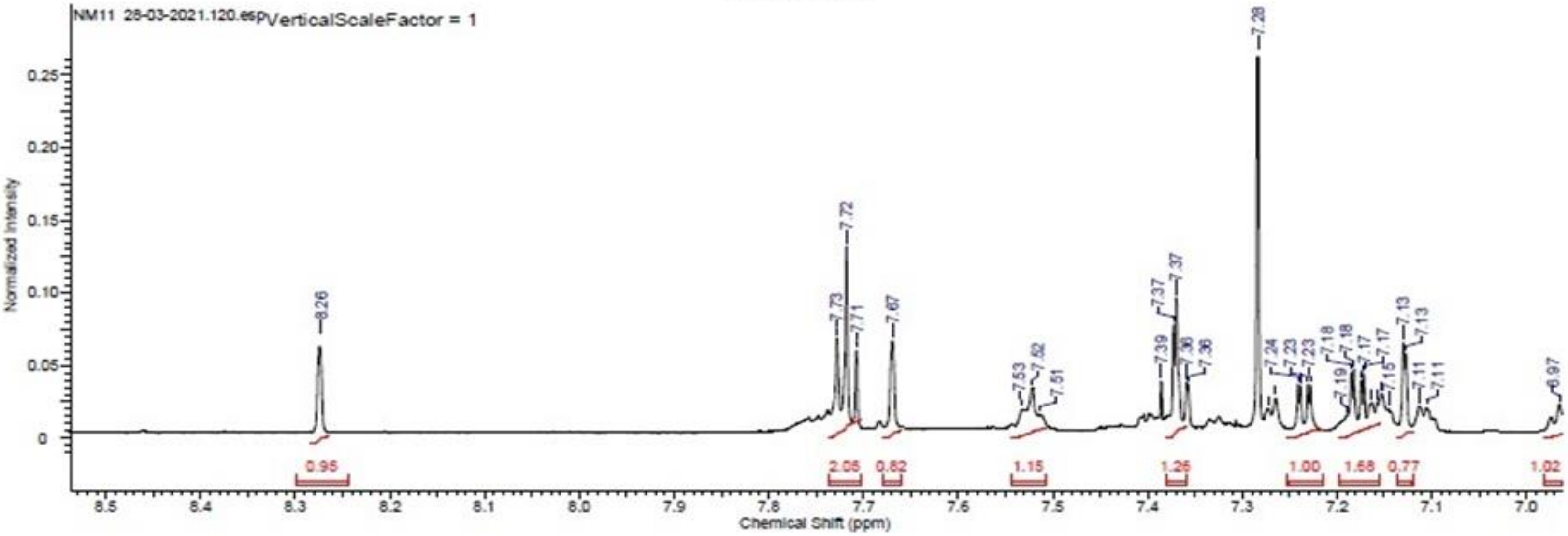

4/30/2021 2:23:49 PM

Acquisition Time (sec)	1.9268	*Comment*	Dr. Abdusattar Sampe 28-03 CDCL3			*Date*	28 Mar 2021 23:09:36
Date Stamp	28 Mar 2021 23:09:36			*File Name*	C:\Users\hp\Desktop\Naproxen NMR\NM12 28-03-2021\130\fid		
Frequency (MHz)	850.15	*Nucleus*	1H	*Number of Transients*	32	*Origin*	spect
Original Points Count	32768	*Owner*	nmr	*Points Count*	32768	*Pulse Sequence*	zg30
Receiver Gain	12.46	*SW(cyclical) (Hz)*	17006.80	*Solvent*	CHLOROFORM-d		
Spectrum Offset (Hz)	5250.0283	*Spectrum Type*	STANDARD	*Sweep Width (Hz)*	17006.28	*Temperature (degree C)*	25.000

Compound 21

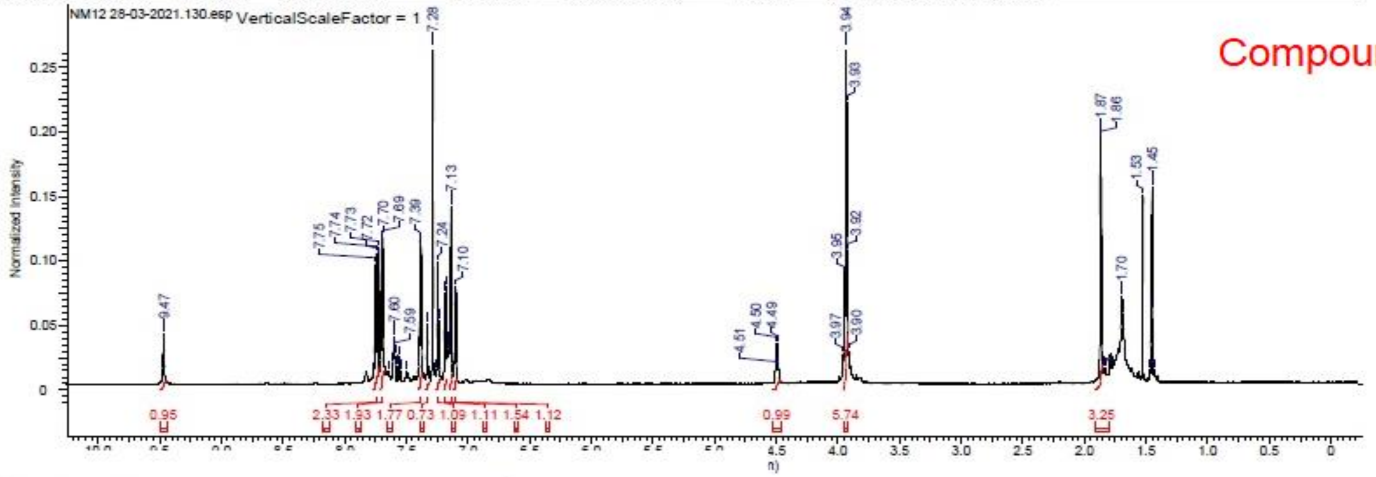

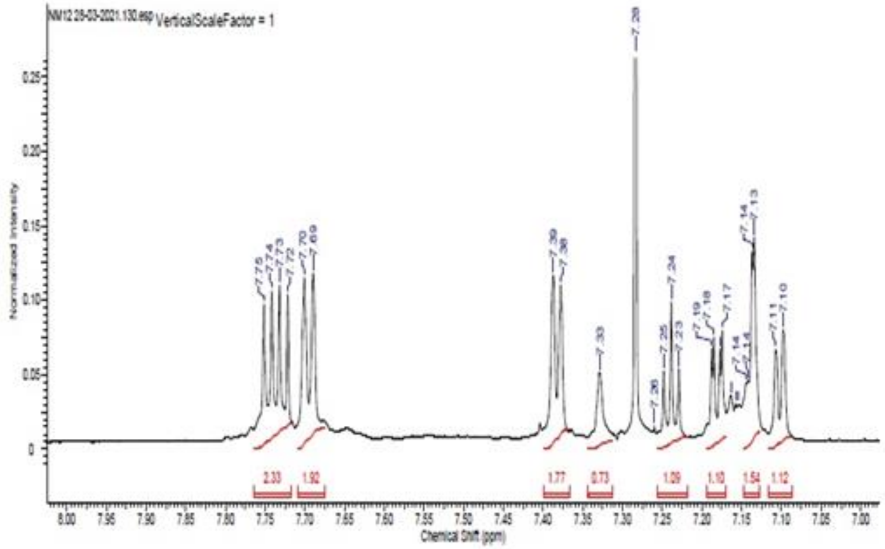

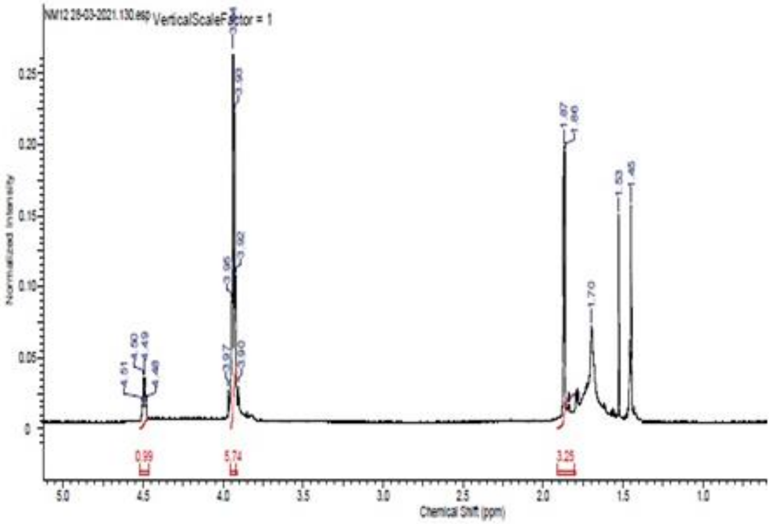

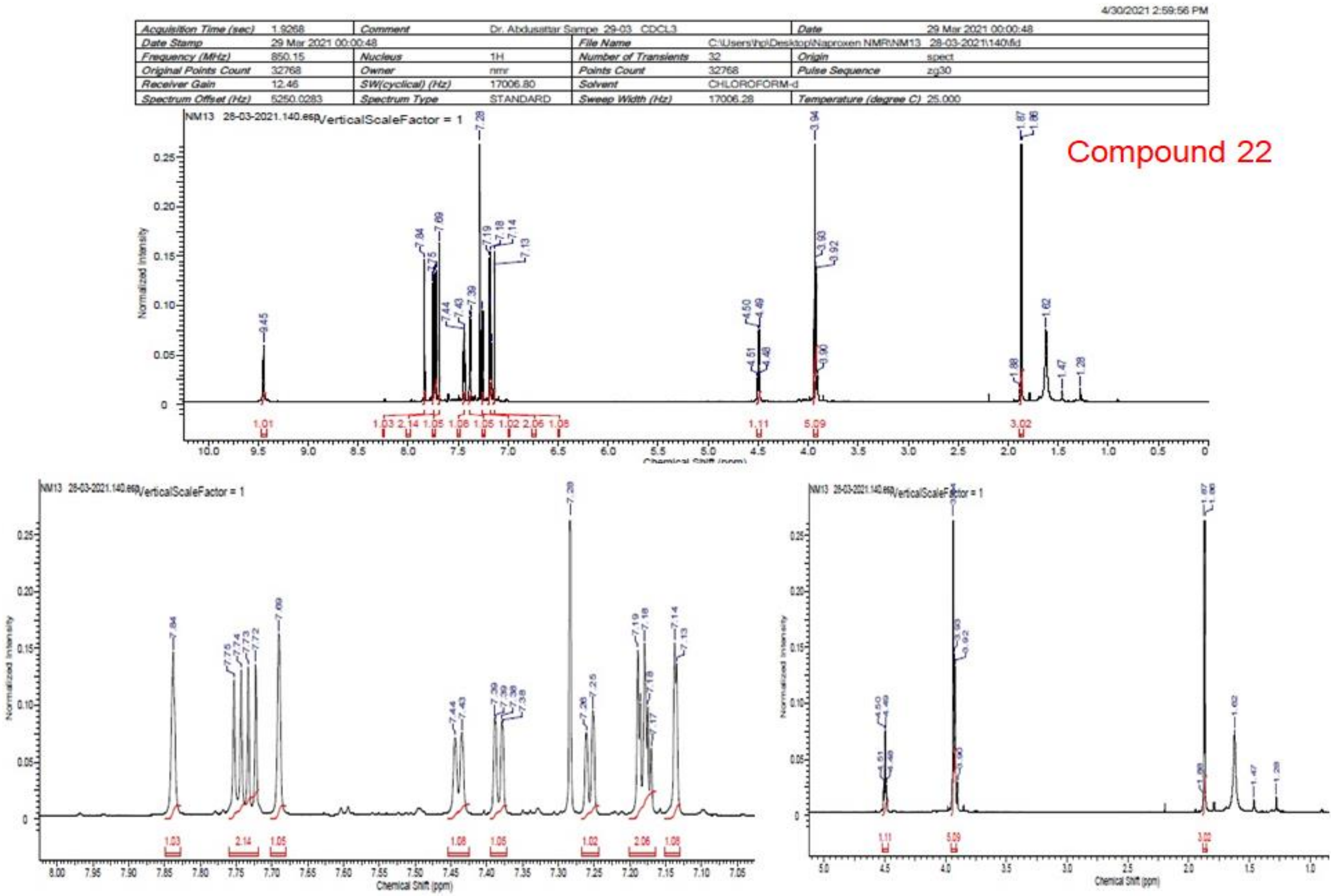
4/30/2021 2:59:56 PM
Acquisition Time (sec) 1.9268
Comment Dr. Abdusattar Sampe 29-03 CDCL3
Date 29 Mar 2021 00:00:48
Date Stamp 29 Mar 2021 00:00:48
File Name C:\Users\hp\Desktop\Naproxen NMR\NM13 28-03-2021\140\fid
Frequency (MHz) 850.15
Nucleus 1H
Number of Transients 32
Origin spect
Original Points Count 32768
Owner nmr
Points Count 32768
Pulse Sequence zg30
Receiver Gain 12.46
SW(cyclical) (Hz) 17006.80
Solvent CHLOROFORM-d
Spectrum Offset (Hz) 5250.0283
Spectrum Type STANDARD
Sweep Width (Hz) 17006.28
Temperature (degree C) 25.000
NM13 28-03-2021.140.esp VerticalScaleFactor = 1
Normalized Intensity
Chemical Shift (ppm)

Compound 22

4/30/2021 4:08:32 PM

Acquisition Time (sec)	1.9268	Comment	Dr. Abdusattar Sampe 33-03 CDCL3			Date	29 Mar 2021 03:34:08
Date Stamp	29 Mar 2021 03:34:08			File Name	C:\Users\hp\Desktop\Naproxen NMR\ NM17 28-03-2021\180\fid		
Frequency (MHz)	850.15	Nucleus	1H	Number of Transients	32	Origin	spect
Original Points Count	32768	Owner	nmr	Points Count	32768	Pulse Sequence	zg30
Receiver Gain	12.46	SW(cyclical) (Hz)	17006.80	Solvent	CHLOROFORM-d		
Spectrum Offset (Hz)	5250.0283	Spectrum Type	STANDARD	Sweep Width (Hz)	17006.28	Temperature (degree C)	24.997

Compound 23

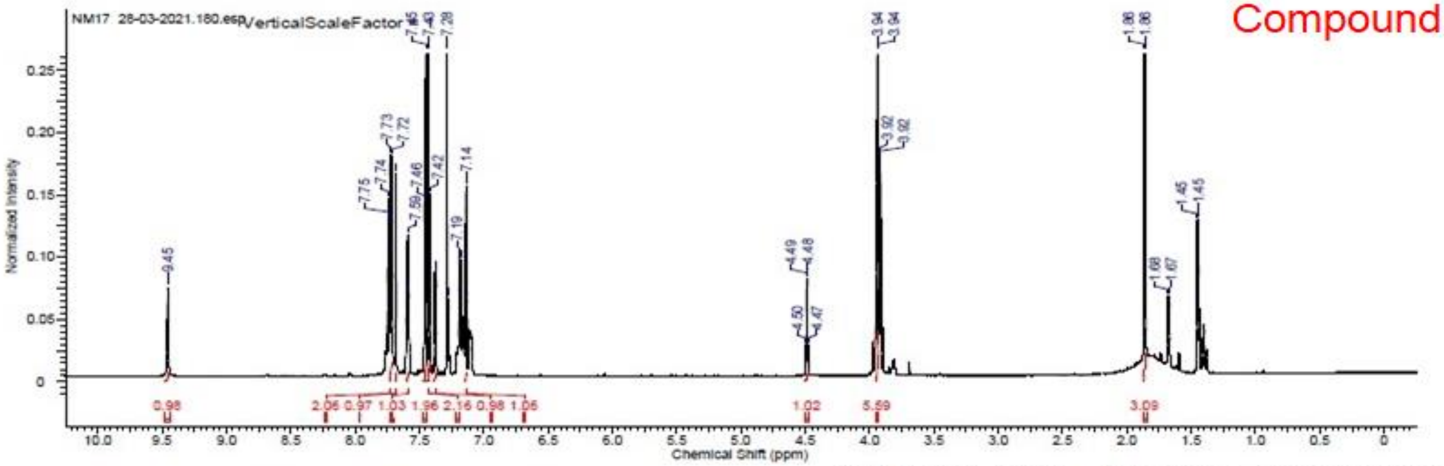

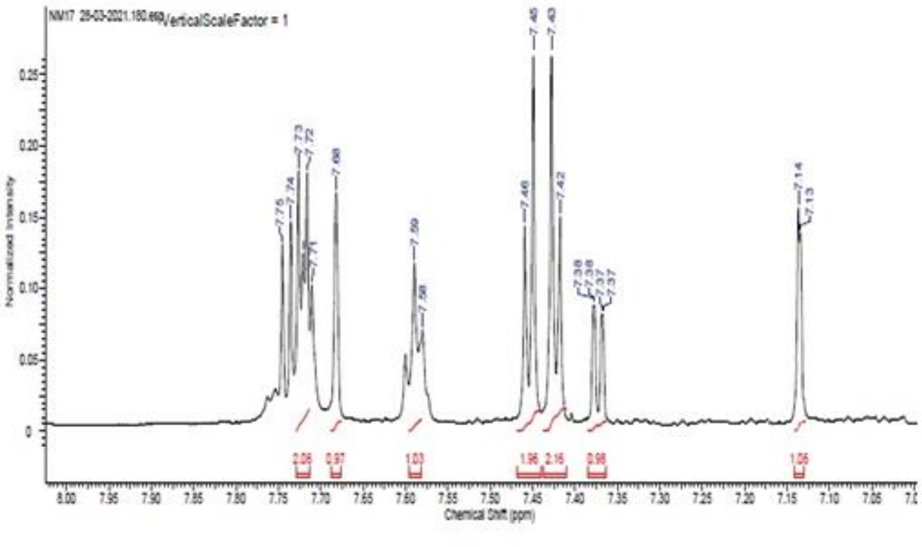

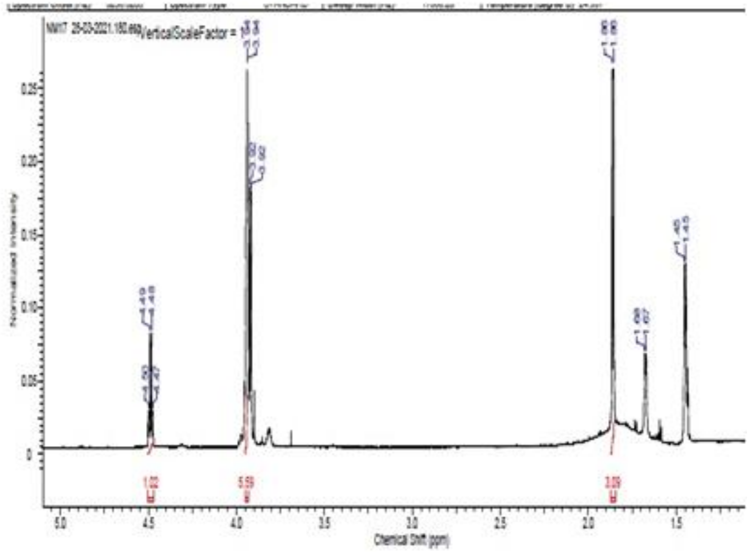

4/30/2021 3:51:45 PM

Acquisition Time (sec)	1.9268	*Comment*	Dr. Abdusattar Sampe 32-03 CDCL3			*Date*	29 Mar 2021 02:40:48
Date Stamp	29 Mar 2021 02:40:48			*File Name*	C:\Users\hp\Desktop\Naproxen NMR\ NM16 28-03-2021\170\fid		
Frequency (MHz)	850.15	*Nucleus*	1H	*Number of Transients*	32	*Origin*	spect
Original Points Count	32768	*Owner*	nmr	*Points Count*	32768	*Pulse Sequence*	zg30
Receiver Gain	12.46	*SW(cyclical) (Hz)*	17006.80	*Solvent*	CHLOROFORM-d		
Spectrum Offset (Hz)	5250.0283	*Spectrum Type*	STANDARD	*Sweep Width (Hz)*	17006.28	*Temperature (degree C)*	24.999

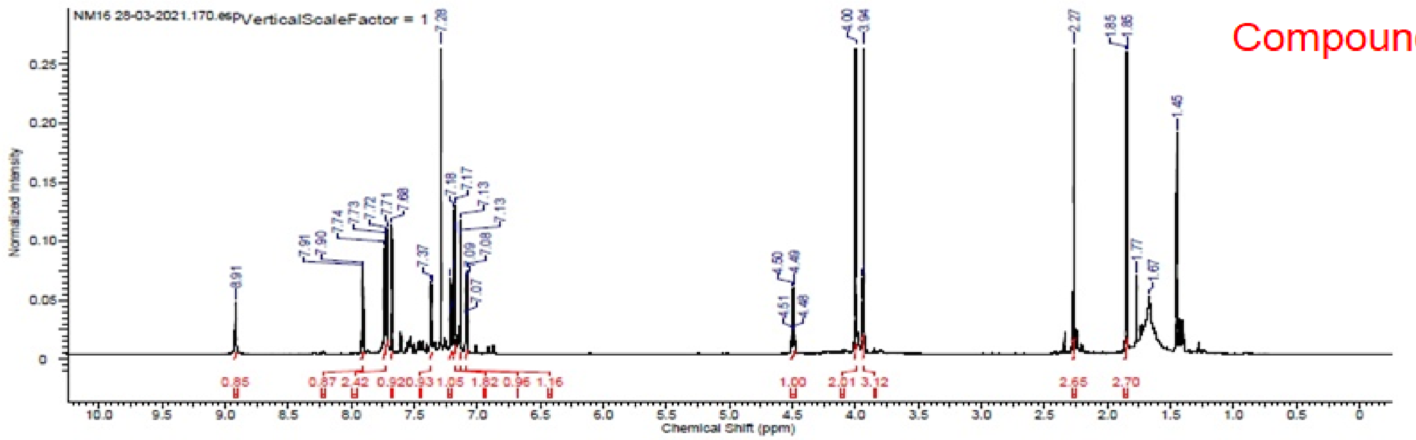

Compound 24

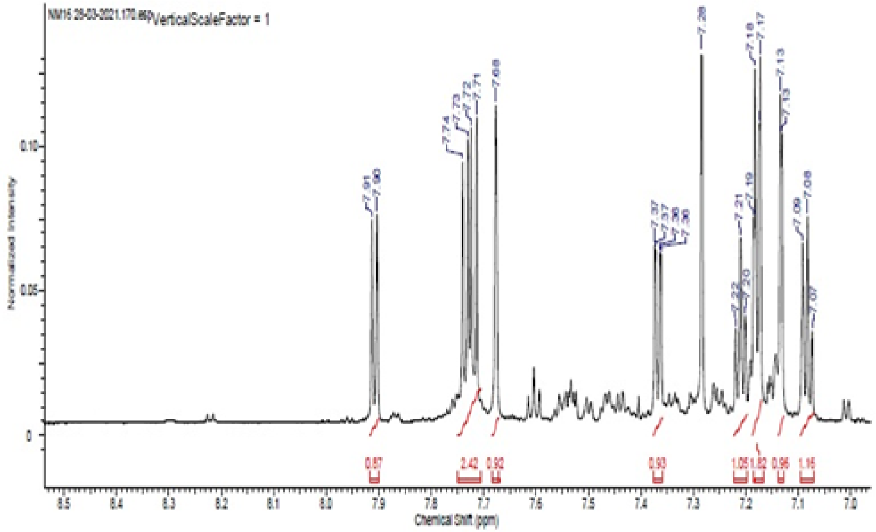

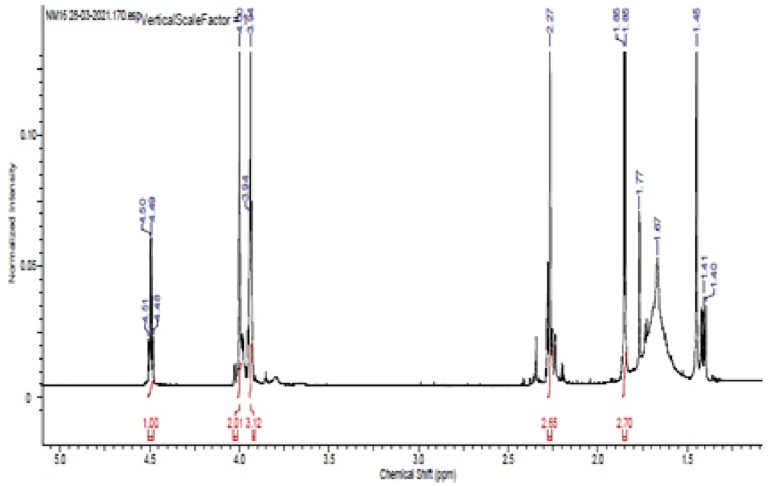

4/30/2021 3:32:40 PM

Acquisition Time (sec)	1.9268	Comment	Dr. Abdusattar Sampe 31-03 CDCL3			Date	29 Mar 2021 01:47:28
Date Stamp	29 Mar 2021 01:47:28			File Name	C:\Users\hp\Desktop\Naproxen NMR\NM15 28-03-2021\160\fid		
Frequency (MHz)	850.15	Nucleus	1H	Number of Transients	32	Origin	spect
Original Points Count	32768	Owner	nmr	Points Count	32768	Pulse Sequence	zg30
Receiver Gain	12.46	SW(cyclical) (Hz)	17006.80	Solvent	CHLOROFORM-d		
Spectrum Offset (Hz)	5250.0283	Spectrum Type	STANDARD	Sweep Width (Hz)	17006.28	Temperature (degree C)	24.998

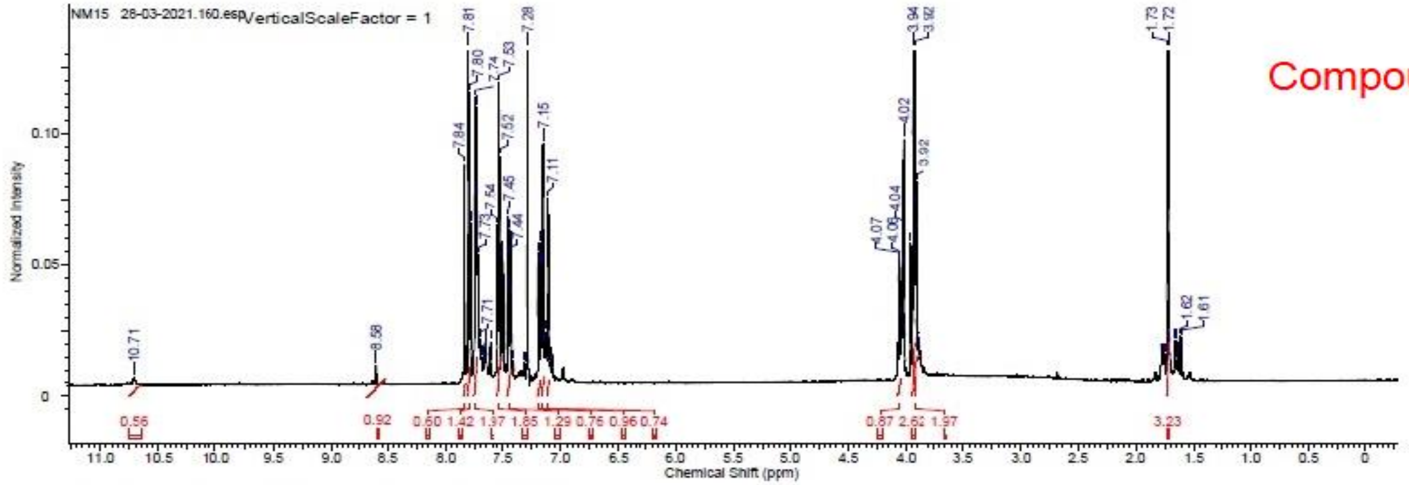

Compound 25

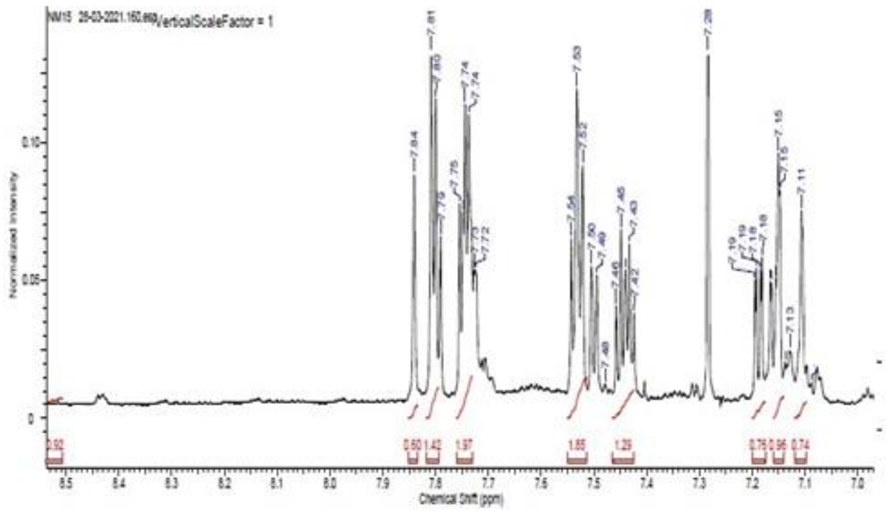

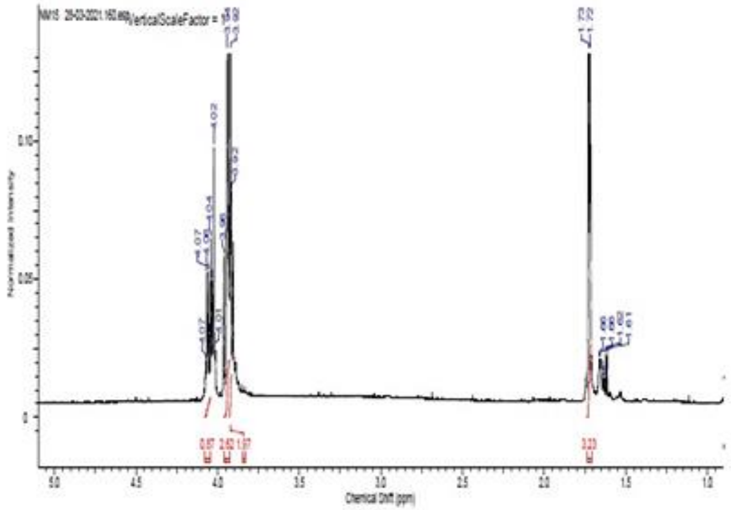

4/30/2021 3:14:40 PM

Acquisition Time (sec)	1.9268	*Comment*	Dr. Abdusattar Sampe 30-03 CDCL3			*Date*	29 Mar 2021 00:56:16
Date Stamp	29 Mar 2021 00:56:16			*File Name*	C:\Users\hp\Desktop\Naproxen NMR\NM14 28-03-2021\150\fid		
Frequency (MHz)	850.15	*Nucleus*	1H	*Number of Transients*	32	*Origin*	spect
Original Points Count	32768	*Owner*	nmr	*Points Count*	32768	*Pulse Sequence*	zg30
Receiver Gain	11.37	*SW(cyclical) (Hz)*	17006.80	*Solvent*	CHLOROFORM-d		
Spectrum Offset (Hz)	5250.0283	*Spectrum Type*	STANDARD	*Sweep Width (Hz)*	17006.28	*Temperature (degree C)*	25.000

Compound 26

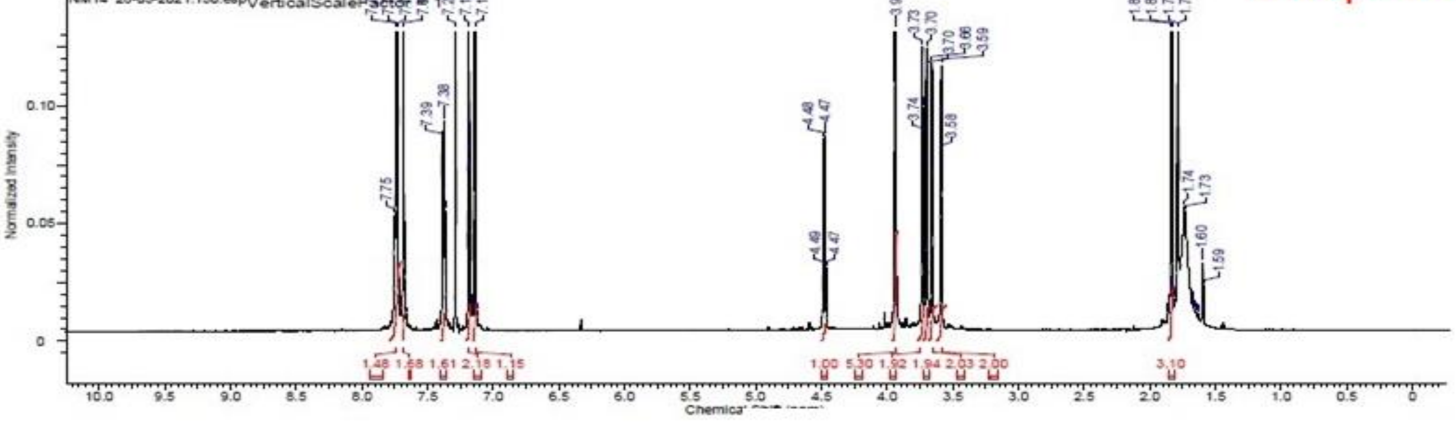

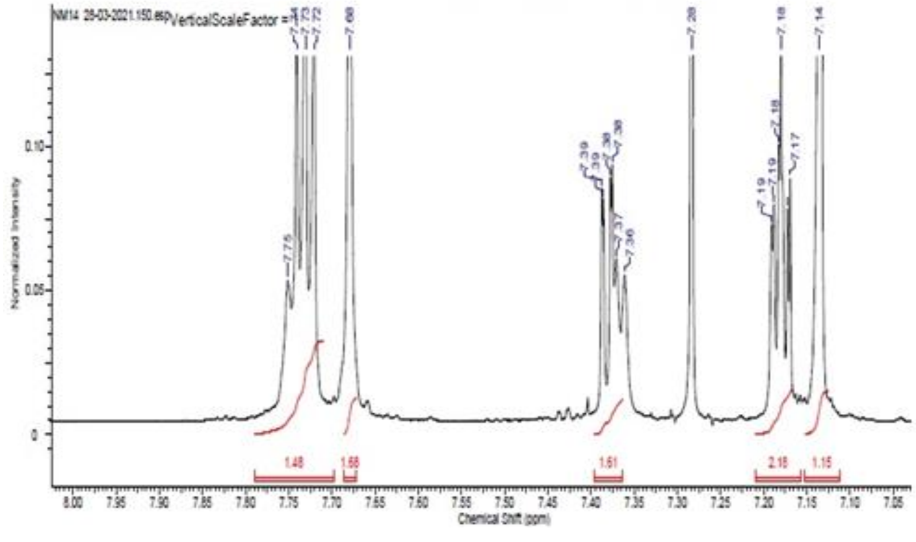

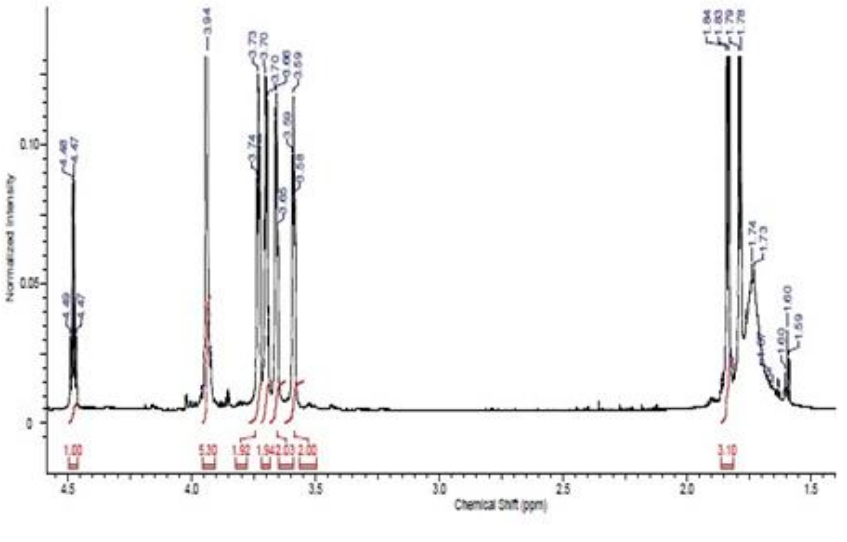

Figure S18-S34: ^{13}C NMR of compounds

Compound 8

4/28/2021 11:14:55 PM

Acquisition Time (sec)	0.6423	*Comment*	Dr. Abdusattar Sampe 17-03 CDCL3	*Date*	28 Mar 2021 14:12:00
Date Stamp	28 Mar 2021 14:12:00	*File Name*	C:\Users\hp\Desktop\Naproxen NMR\NM1 28-03-2021\21\fid		
Frequency (MHz)	213.77	*Nucleus*	13C	*Number of Transients*	1024	*Origin*	spect
Original Points Count	32768	*Owner*	nmr	*Points Count*	32768	*Pulse Sequence*	zgpg30
Receiver Gain	186.93	*SW(cyclical) (Hz)*	51020.41	*Solvent*	CHLOROFORM-d		
Spectrum Offset (Hz)	21376.0332	*Spectrum Type*	STANDARD	*Sweep Width (Hz)*	51018.85	*Temperature (degree C)*	25.000

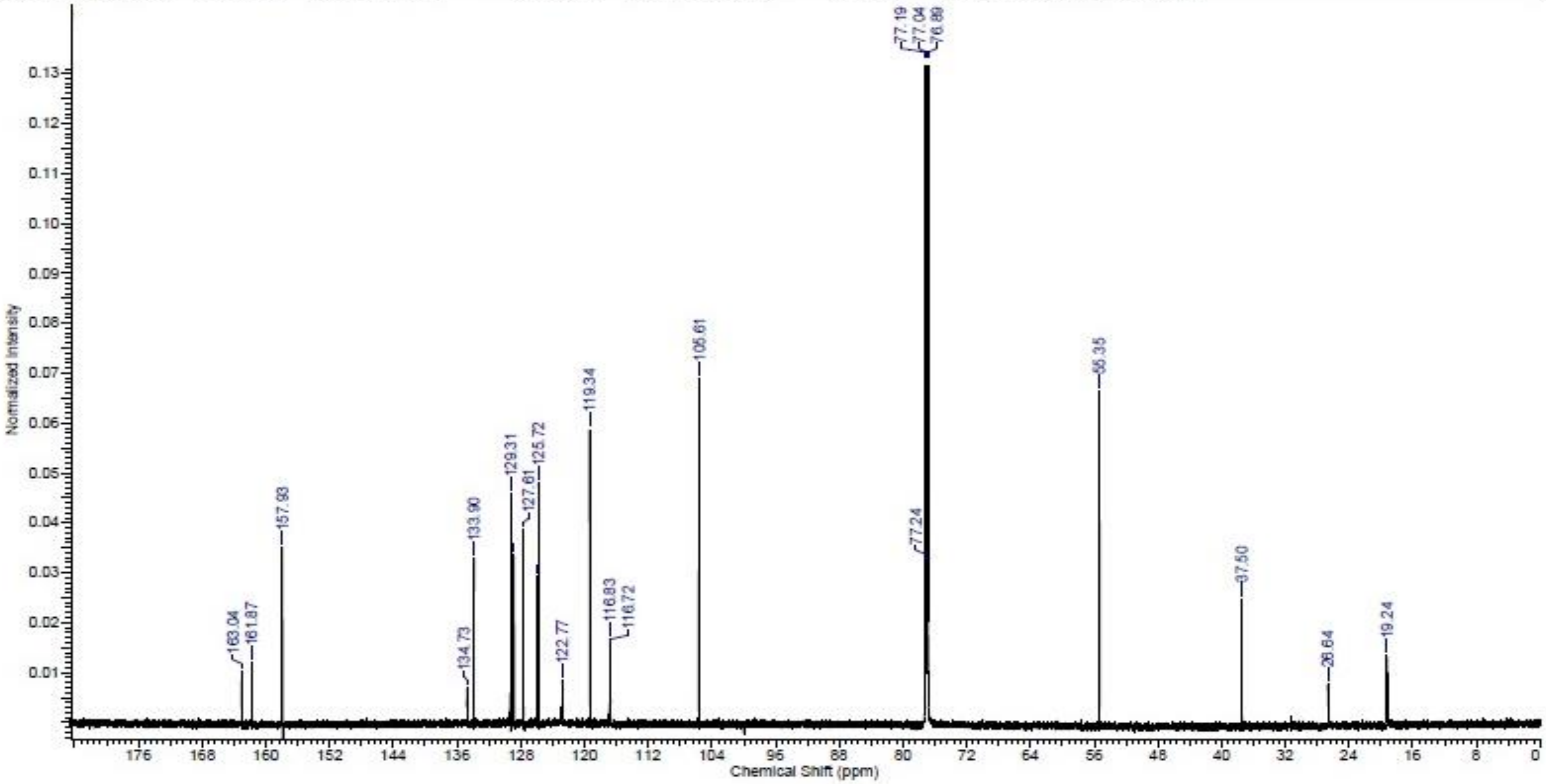

Compound 9

4/28/2021 10:44:41 PM

Acquisition Time (sec)	0.6423	*Comment*	Dr. Abdusattar Sampe 20-03 CDCL3			*Date*	28 Mar 2021 16:52:00
Date Stamp	28 Mar 2021 16:52:00			*File Name*	C:\Users\hp\Desktop\Naproxen NMR\NM4 28-03-2021\51\fid		
Frequency (MHz)	213.77	*Nucleus*	13C	*Number of Transients*	1024	*Origin*	spect
Original Points Count	32768	*Owner*	nmr	*Points Count*	32768	*Pulse Sequence*	zgpg30
Receiver Gain	186.93	*SW(cyclical) (Hz)*	51020.41	*Solvent*	CHLOROFORM-d		
Spectrum Offset (Hz)	21376.0332	*Spectrum Type*	STANDARD	*Sweep Width (Hz)*	51018.85	*Temperature (degree C)*	24.999

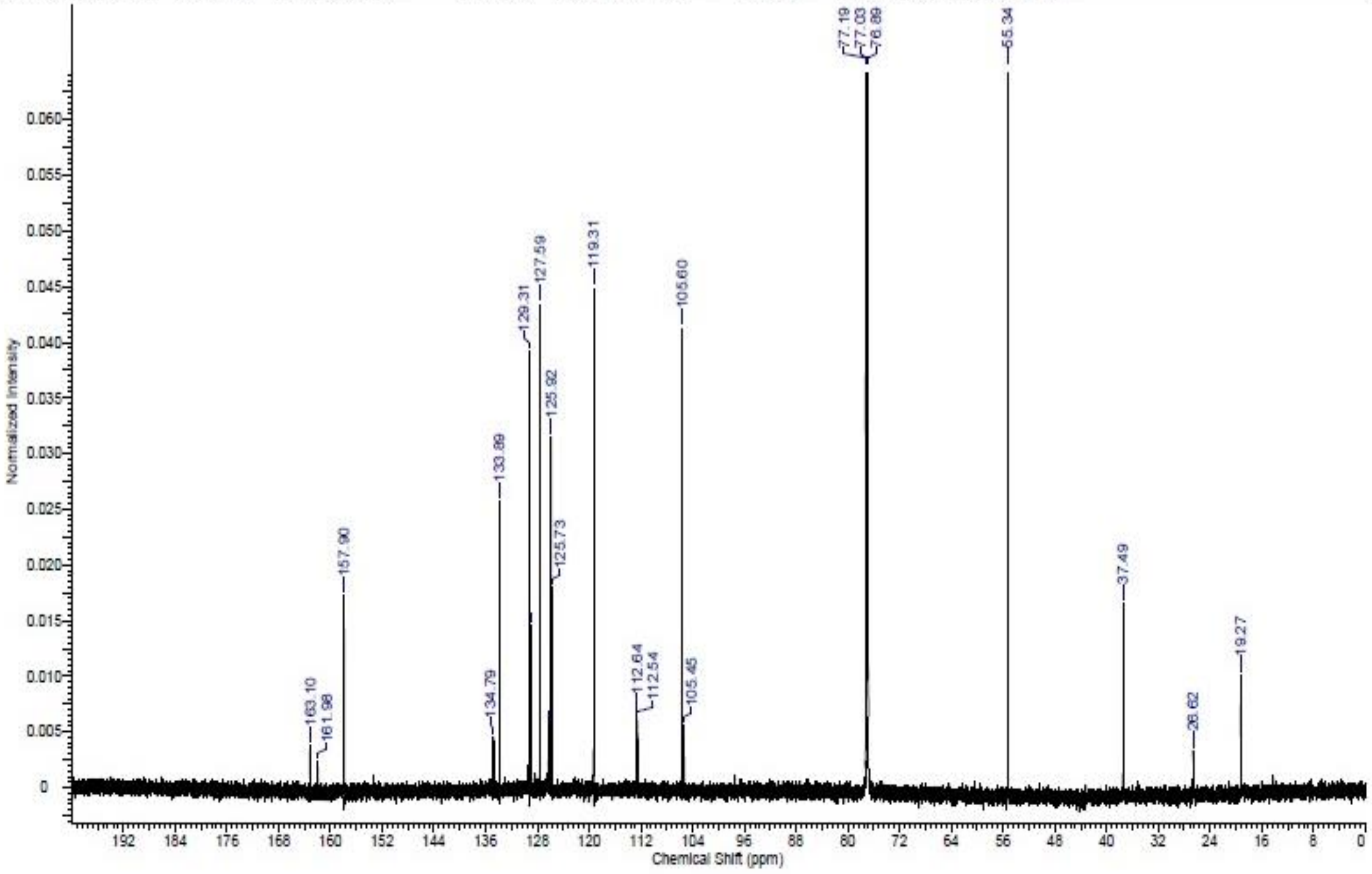

Compound 10

4/28/2021 10:39:20 PM

Acquisition Time (sec)	0.6423	*Comment*	Dr. Abdusattar Sampe 23-03 CDCL3			*Date*	28 Mar 2021 19:32:00
Date Stamp	28 Mar 2021 19:32:00			*File Name*	C:\Users\hp\Desktop\Naproxen NMR\NM7 28-03-2021\81\fid		
Frequency (MHz)	213.77	*Nucleus*	13C	*Number of Transients*	1024	*Origin*	spect
Original Points Count	32768	*Owner*	nmr	*Points Count*	32768	*Pulse Sequence*	zgpg30
Receiver Gain	186.93	*SW(cyclical) (Hz)*	51020.41	*Solvent*	CHLOROFORM-d		
Spectrum Offset (Hz)	21376.0332	*Spectrum Type*	STANDARD	*Sweep Width (Hz)*	51018.85	*Temperature (degree C)*	25.001

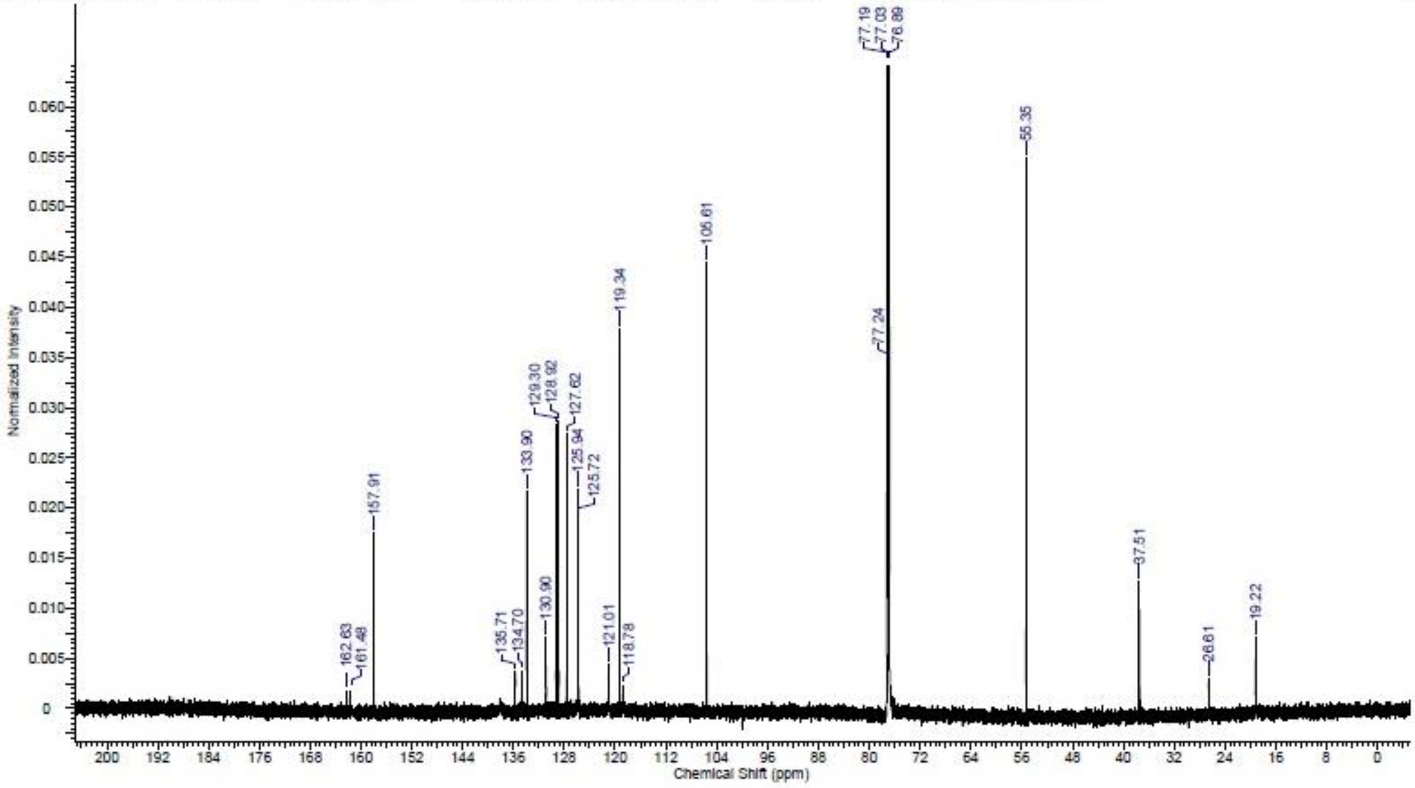

Compound 11

3/30/2021 2:05:53 AM

Acquisition Time (sec)	0.6423	Comment	Dr. Abdusattar Sampe 25-03 CDCL3			Date	28 Mar 2021 21:18:40
Date Stamp	28 Mar 2021 21:18:40			File Name	C:\Users\hp\Desktop\Naproxen NMR\NM9 28-03-2021\102\fid		
Frequency (MHz)	213.77	Nucleus	13C	Number of Transients	1024	Origin	spect
Original Points Count	32768	Owner	nmr	Points Count	32768	Pulse Sequence	zgpg30
Receiver Gain	186.93	SW(cyclical) (Hz)	51020.41	Solvent	CHLOROFORM-d		
Spectrum Offset (Hz)	21376.0332	Spectrum Type	STANDARD	Sweep Width (Hz)	51018.85	Temperature (degree C)	24.999

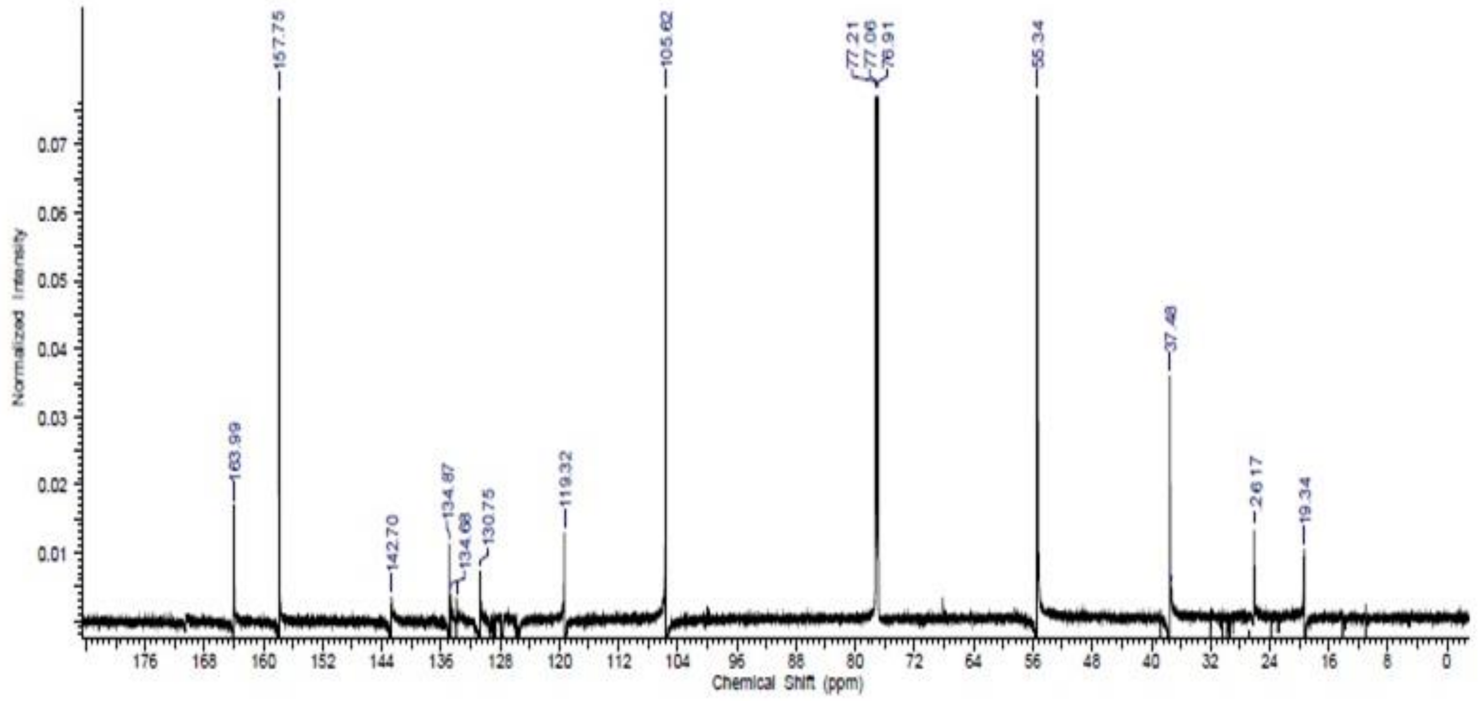

Compound 12

4/28/2021 10:49:36 PM

Acquisition Time (sec)	0.6423	*Comment*	Dr. Abdusattar Sampe 18-03 CDCL3			*Date*	28 Mar 2021 15:07:28
Date Stamp	28 Mar 2021 15:07:28			*File Name*	C:\Users\hp\Desktop\Naproxen NMR\NM2 28-03-2021\31\fid		
Frequency (MHz)	213.77	*Nucleus*	13C	*Number of Transients*	1024	*Origin*	spect
Original Points Count	32768	*Owner*	nmr	*Points Count*	32768	*Pulse Sequence*	zgpg30
Receiver Gain	186.93	*SW(cyclical) (Hz)*	51020.41	*Solvent*	CHLOROFORM-d		
Spectrum Offset (Hz)	21376.0332	*Spectrum Type*	STANDARD	*Sweep Width (Hz)*	51018.85	*Temperature (degree C)*	25.000

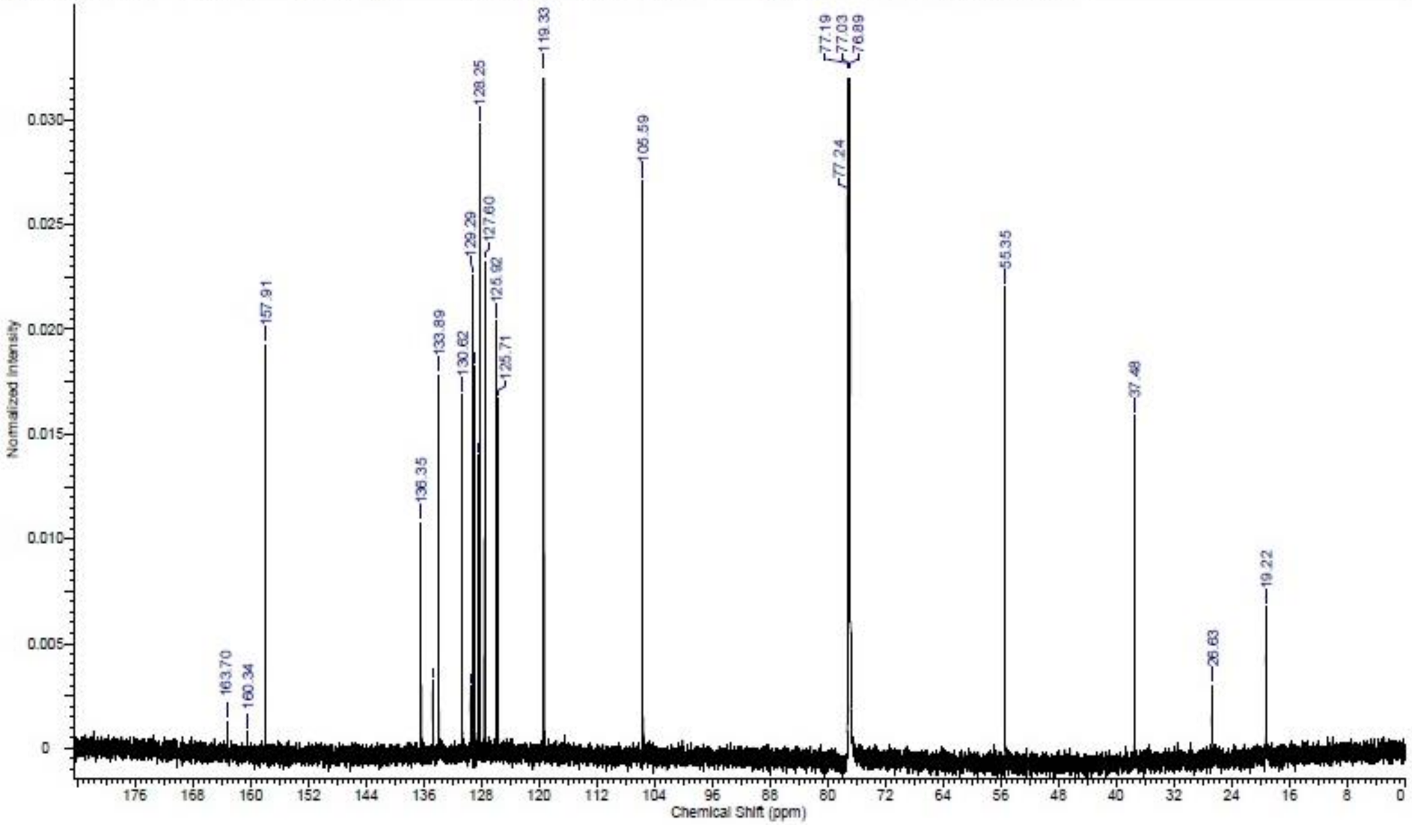

Compound 13

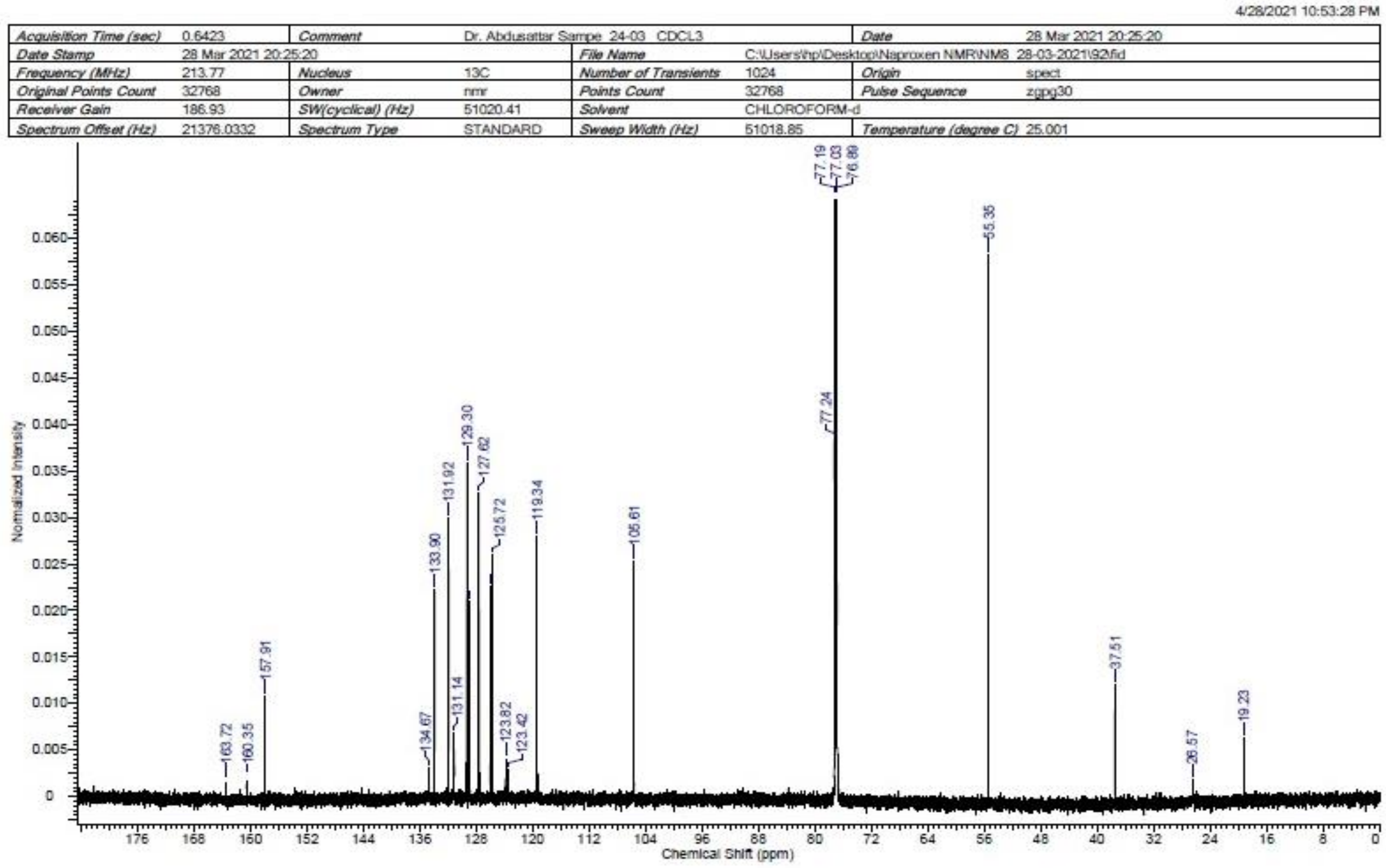
4/28/2021 10:53:28 PM
Acquisition Time (sec) 0.6423
Comment Dr. Abdusattar Sampe 24-03 CDCL3
Date 28 Mar 2021 20:25:20
Date Stamp 28 Mar 2021 20:25:20
File Name C:\Users\hp\Desktop\Naproxen NMR\NM8 28-03-2021\92\fid
Frequency (MHz) 213.77
Nucleus 13C
Number of Transients 1024
Origin spect
Original Points Count 32768
Owner nmr
Points Count 32768
Pulse Sequence zgpg30
Receiver Gain 186.93
SW(cyclical) (Hz) 51020.41
Solvent CHLOROFORM-d
Spectrum Offset (Hz) 21376.0332
Spectrum Type STANDARD
Sweep Width (Hz) 51018.85
Temperature (degree C) 25.001
163.72
160.35
157.91
134.67
133.90
131.92
131.14
129.30
127.62
125.72
123.82
123.42
119.34
105.61
77.24
77.19
77.03
76.89
55.35
37.51
26.57
19.23
Normalized Intensity
Chemical Shift (ppm)

Compound 14

4/28/2021 11:01:03 PM

Acquisition Time (sec)	0.6423	Comment	Dr. Abdusattar Sampe 21-03 CDCL3			Date	28 Mar 2021 17:45:20
Date Stamp	28 Mar 2021 17:45:20			File Name	C:\Users\hp\Desktop\Naproxen NMR\NM5 28-03-2021\61\fid		
Frequency (MHz)	213.77	Nucleus	13C	Number of Transients	1024	Origin	spect
Original Points Count	32768	Owner	nmr	Points Count	32768	Pulse Sequence	zgpg30
Receiver Gain	186.93	SW(cyclical) (Hz)	51020.41	Solvent	CHLOROFORM-d		
Spectrum Offset (Hz)	21376.0332	Spectrum Type	STANDARD	Sweep Width (Hz)	51018.85	Temperature (degree C)	25.001

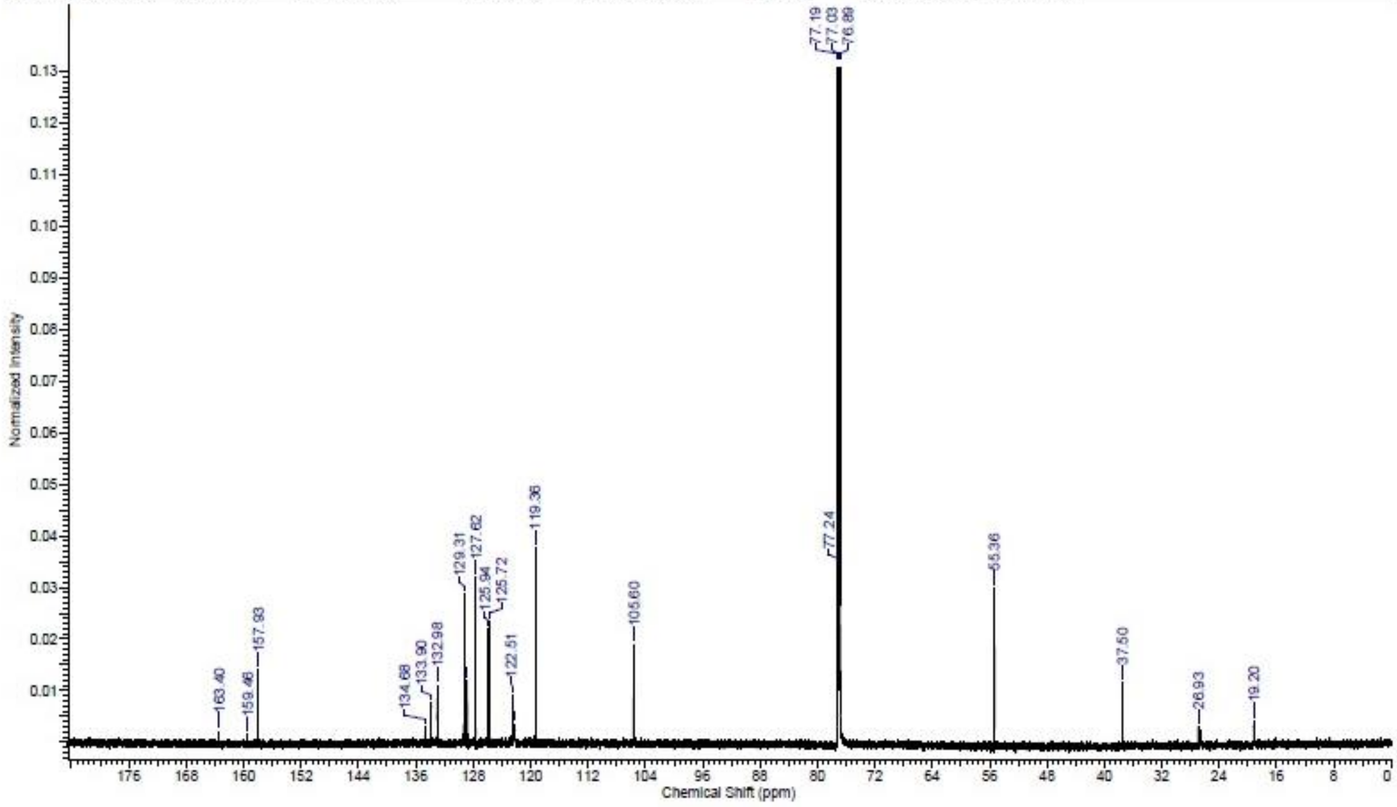

Compound 15

4/28/2021 11:05:23 PM

Acquisition Time (sec)	0.6423	*Comment*	Dr. Abdusattar Sampe 22-03 CDCL3			*Date*	28 Mar 2021 18:15:12
Date Stamp	28 Mar 2021 18:15:12			*File Name*	C:\Users\hp\Desktop\Naproxen NMR\NM6 28-03-2021\71\fid		
Frequency (MHz)	213.77	*Nucleus*	13C	*Number of Transients*	1024	*Origin*	spect
Original Points Count	32768	*Owner*	nmr	*Points Count*	32768	*Pulse Sequence*	zgpg30
Receiver Gain	186.93	*SW(cyclical) (Hz)*	51020.41	*Solvent*	CHLOROFORM-d		
Spectrum Offset (Hz)	21376.0332	*Spectrum Type*	STANDARD	*Sweep Width (Hz)*	51018.85	*Temperature (degree C)*	25.001

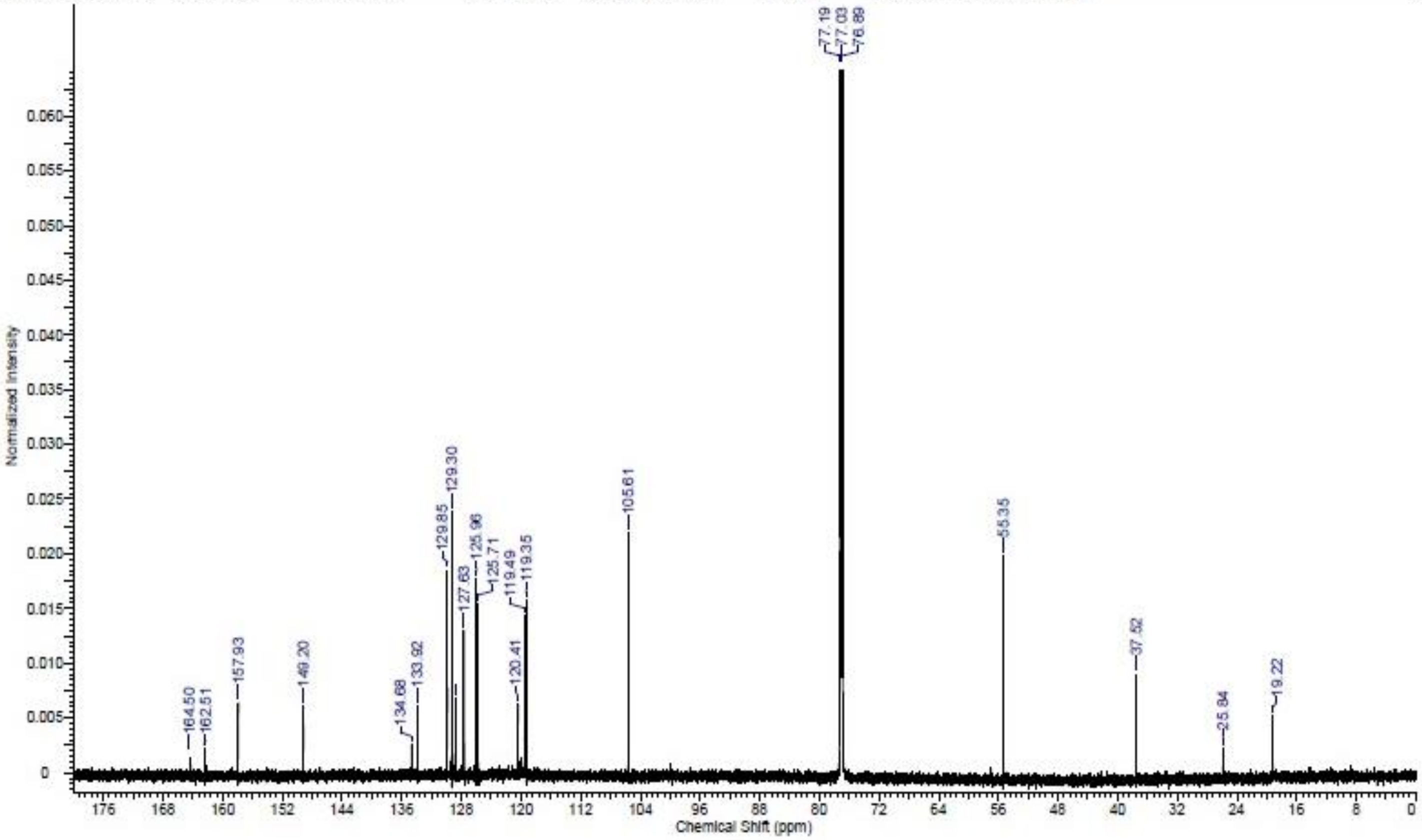

Compound 16

4/28/2021 11:11:25 PM

Acquisition Time (sec)	0.6423	*Comment*	Dr. Abdusattar Sampe 19-03 CDCL3			*Date*	28 Mar 2021 15:58:40
Date Stamp	28 Mar 2021 15:58:40			*File Name*	C:\Users\hp\Desktop\Naproxen NMR\NM3 28-03-2021\41\fid		
Frequency (MHz)	213.77	*Nucleus*	13C	*Number of Transients*	1024	*Origin*	spect
Original Points Count	32768	*Owner*	nmr	*Points Count*	32768	*Pulse Sequence*	zgpg30
Receiver Gain	186.93	*SW(cyclical) (Hz)*	51020.41	*Solvent*	CHLOROFORM-d		
Spectrum Offset (Hz)	21376.0332	*Spectrum Type*	STANDARD	*Sweep Width (Hz)*	51018.85	*Temperature (degree C)*	24.999

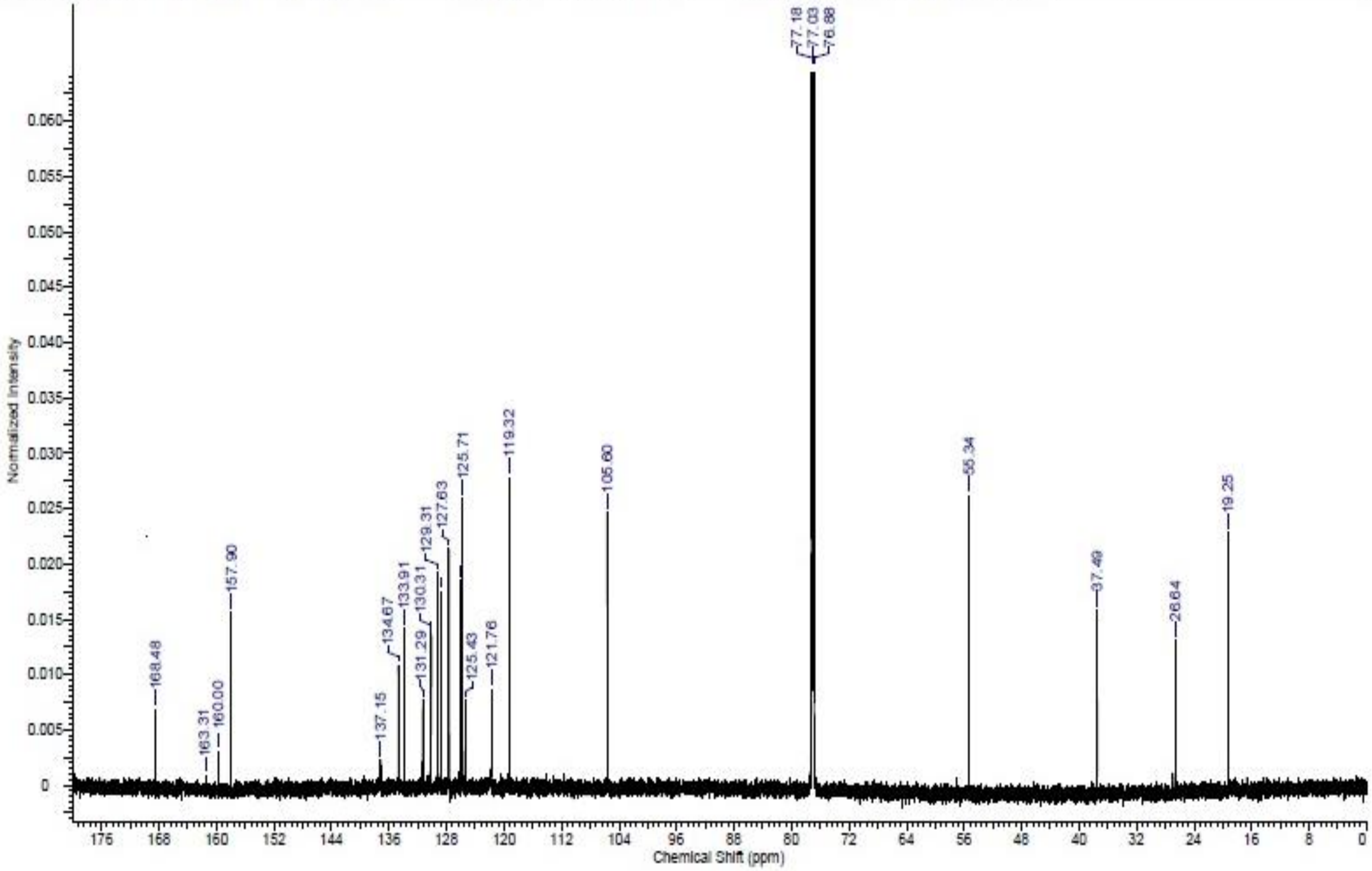

Compound 19

4/28/2021 11:25:19 PM

Acquisition Time (sec)	0.6423	*Comment*	Dr. Abdusattar Sampe 26-03 CDCL3			*Date*	28 Mar 2021 22:12:00
Date Stamp	28 Mar 2021 22:12:00			*File Name*	C:\Users\hp\Desktop\Naproxen NMR\NM10 28-03-2021\112\fid		
Frequency (MHz)	213.77	*Nucleus*	13C	*Number of Transients*	1024	*Origin*	spect
Original Points Count	32768	*Owner*	nmr	*Points Count*	32768	*Pulse Sequence*	zgpg30
Receiver Gain	186.93	*SW(cyclical) (Hz)*	51020.41	*Solvent*	CHLOROFORM-d		
Spectrum Offset (Hz)	21376.0332	*Spectrum Type*	STANDARD	*Sweep Width (Hz)*	51018.85	*Temperature (degree C)*	25.001

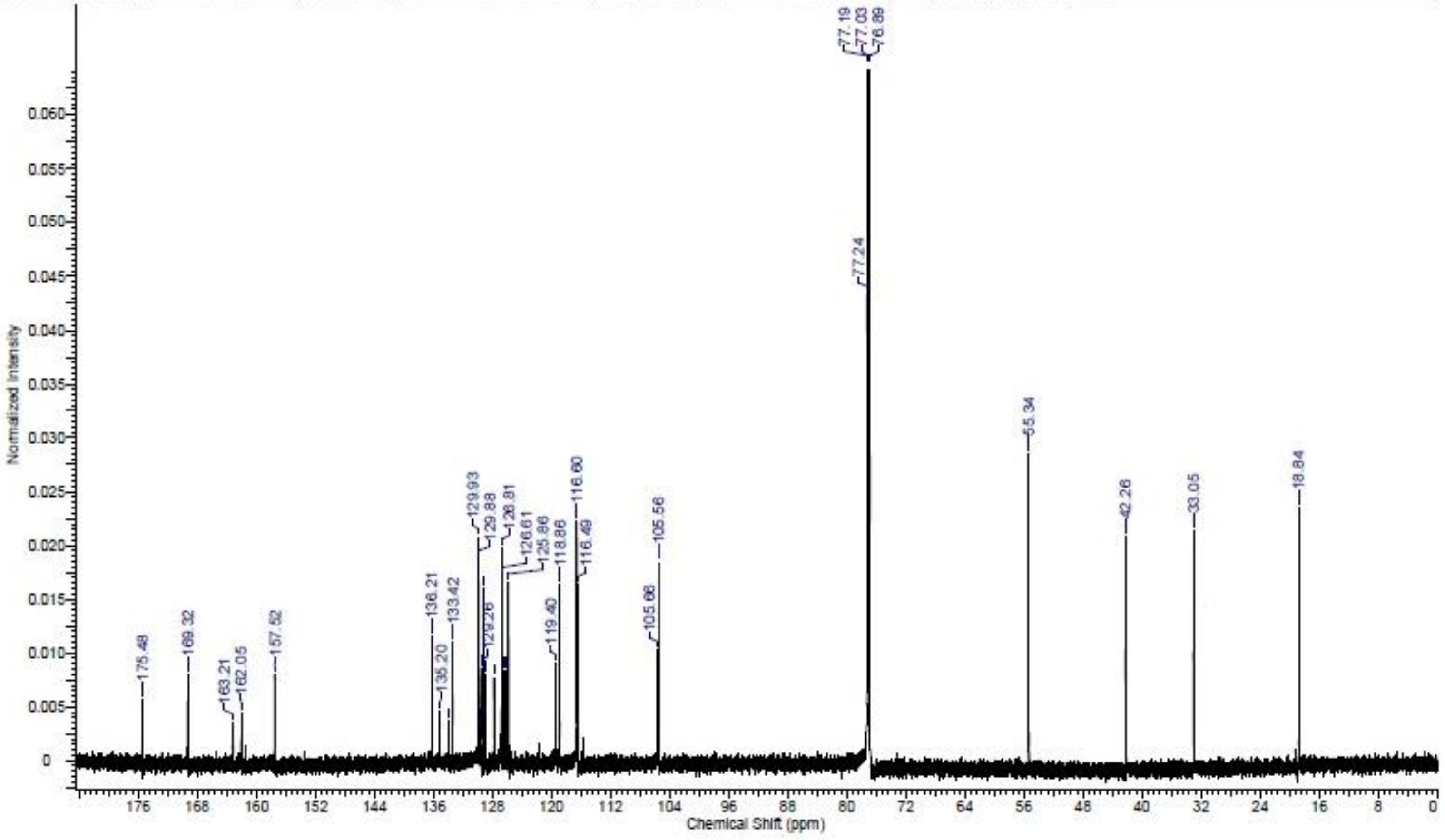

Compound 20

4/28/2021 11:30:24 PM

Acquisition Time (sec)	0.6423	*Comment*	Dr. Abdusattar Sampe 27-03 CDCL3			*Date*	28 Mar 2021 23:03:12
Date Stamp	28 Mar 2021 23:03:12			*File Name*	C:\Users\hp\Desktop\Naproxen NMR\NM11 28-03-2021\122\fid		
Frequency (MHz)	213.77	*Nucleus*	13C	*Number of Transients*	1024	*Origin*	spect
Original Points Count	32768	*Owner*	nmr	*Points Count*	32768	*Pulse Sequence*	zgpg30
Receiver Gain	186.93	*SW(cyclical) (Hz)*	51020.41	*Solvent*	CHLOROFORM-d		
Spectrum Offset (Hz)	21376.0332	*Spectrum Type*	STANDARD	*Sweep Width (Hz)*	51018.85	*Temperature (degree C)*	25.002

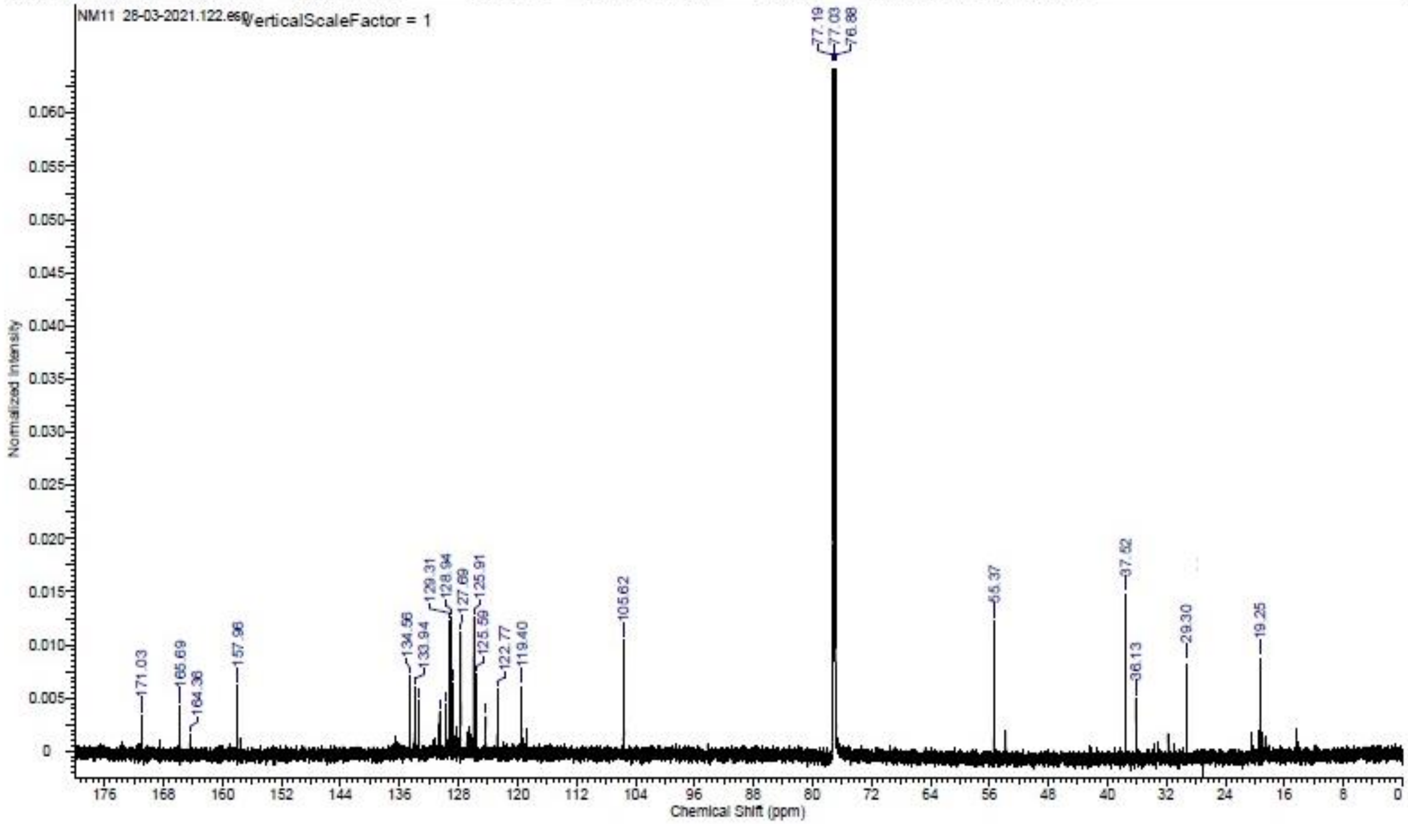

Compound 21

4/28/2021 11:34:12 PM

Acquisition Time (sec)	0.6423	Comment	Dr. Abdusattar Sampe 28-03 CDCL3			Date	28 Mar 2021 23:56:32
Date Stamp	28 Mar 2021 23:56:32			File Name	C:\Users\hp\Desktop\Naproxen NMR\NM12 28-03-2021\131\fid		
Frequency (MHz)	213.77	Nucleus	13C	Number of Transients	1024	Origin	spect
Original Points Count	32768	Owner	nmr	Points Count	32768	Pulse Sequence	zgpg30
Receiver Gain	186.93	SW(cyclical) (Hz)	51020.41	Solvent	CHLOROFORM-d		
Spectrum Offset (Hz)	21376.0332	Spectrum Type	STANDARD	Sweep Width (Hz)	51018.85	Temperature (degree C)	25.002

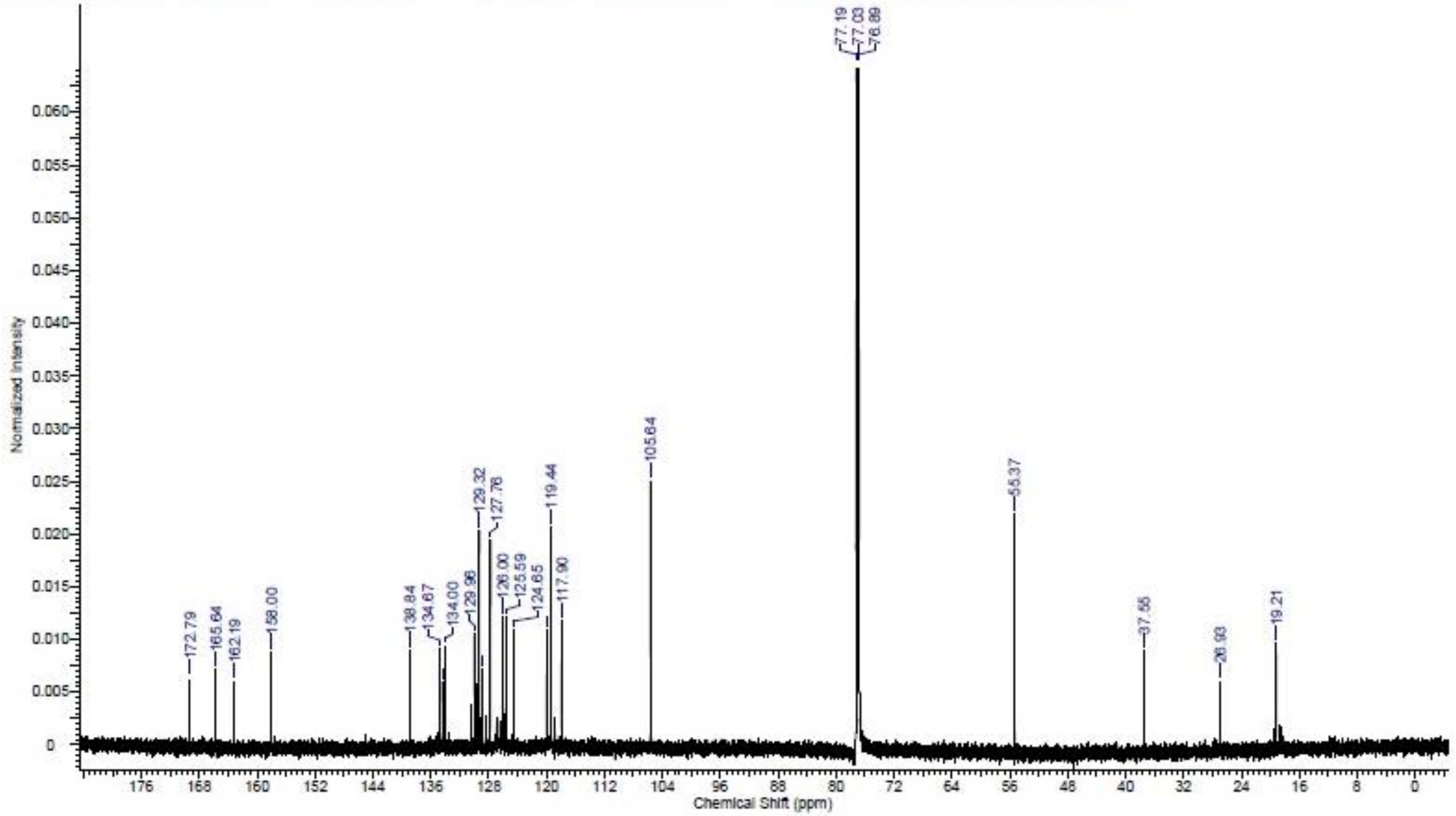

Compound 22

3/31/2021 12:42:44 AM

Acquisition Time (sec)	0.6423	Comment	Dr. Abdusattar Sampe 29-03 CDCL3			Date	29 Mar 2021 00:49:52
Date Stamp	29 Mar 2021 00:49:52			File Name	C:\Users\hp\Desktop\Naproxen NMR\NM13 28-03-2021\141\fid		
Frequency (MHz)	213.77	Nucleus	13C	Number of Transients	1024	Origin	spect
Original Points Count	32768	Owner	nmr	Points Count	32768	Pulse Sequence	zgpg30
Receiver Gain	186.93	SW(cyclical) (Hz)	51020.41	Solvent	CHLOROFORM-d		
Spectrum Offset (Hz)	21376.0332	Spectrum Type	STANDARD	Sweep Width (Hz)	51018.85	Temperature (degree C)	25.000

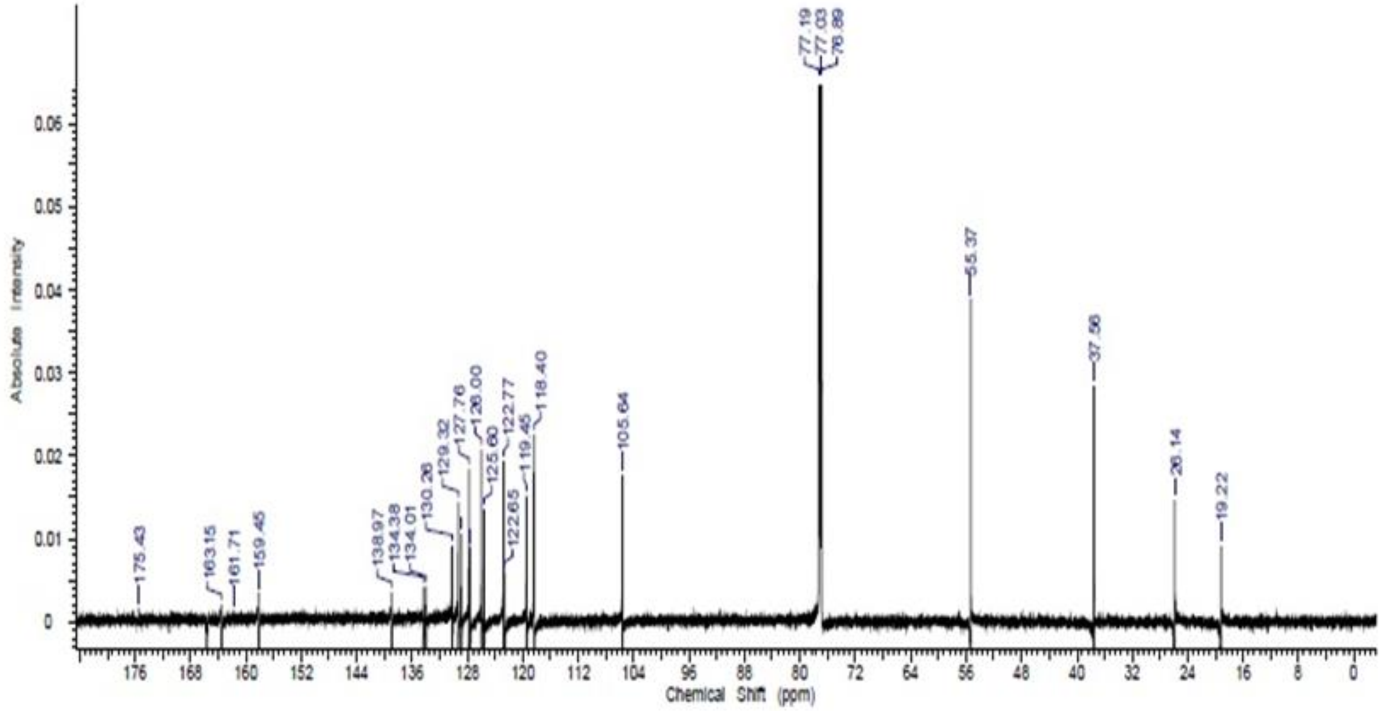

Compound 23

4/16/2021 6:03:48 PM

RMS of Noise 507469.5313

Acquisition Time (sec)	0.6423	*Comment*	Dr. Abdusattar Sampe 33-03 CDCL3			*Date*	29 Mar 2021 04:21:04
Date Stamp	29 Mar 2021 04:21:04			*File Name*	C:\Users\hp\Desktop\Naproxen NMR\ NM17 28-03-2021\181\fid		
Frequency (MHz)	213.77	*Nucleus*	13C	*Number of Transients*	1024	*Origin*	spect
Original Points Count	32768	*Owner*	nmr	*Points Count*	32768	*Pulse Sequence*	zgpg30
Receiver Gain	186.93	*SW(cyclical) (Hz)*	51020.41	*Solvent*	CHLOROFORM-d		
Spectrum Offset (Hz)	21376.0332	*Spectrum Type*	STANDARD	*Sweep Width (Hz)*	51018.85	*Temperature (degree C)*	25.000

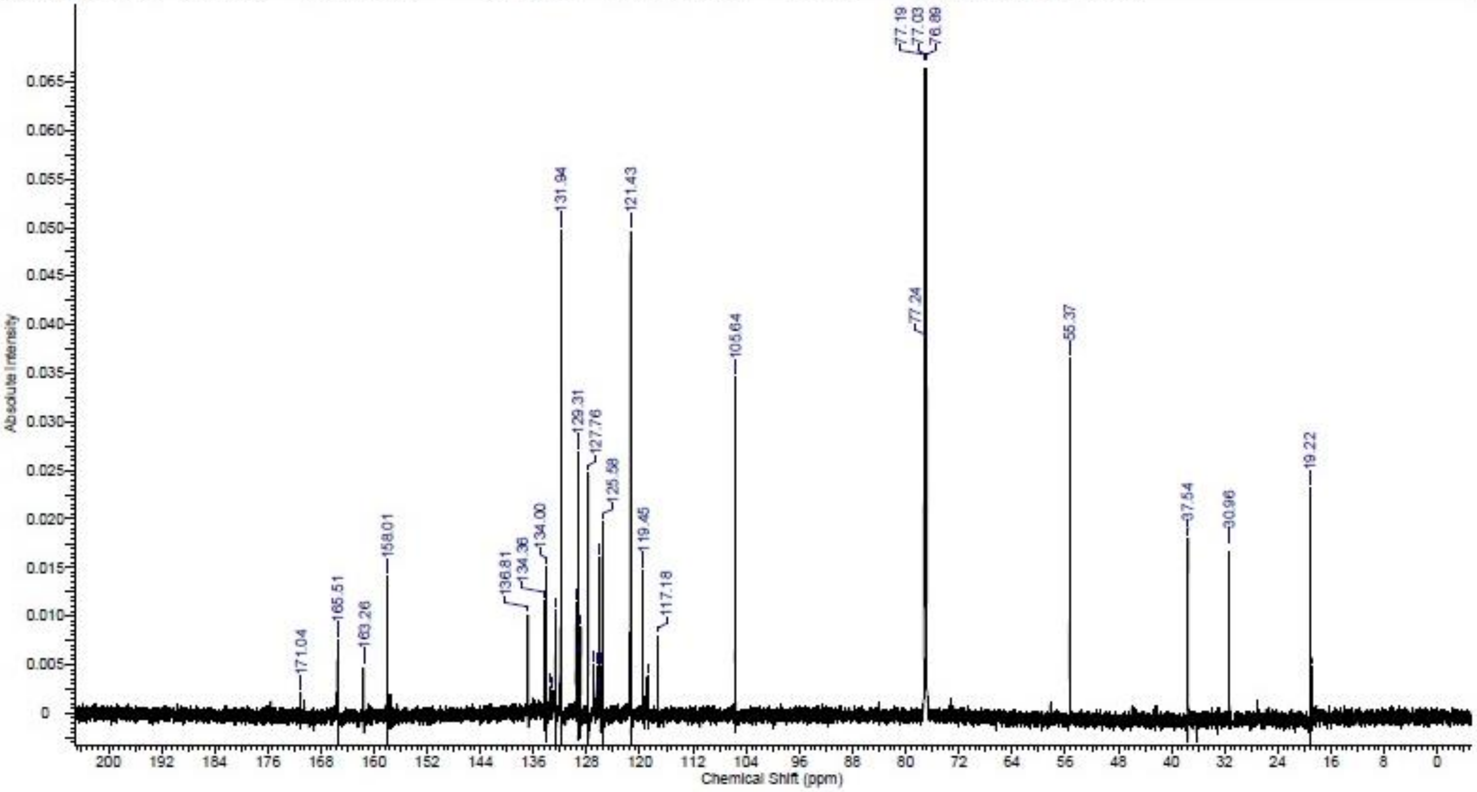

Compound 24

4/17/2021 2:16:28 AM

Acquisition Time (sec)	0.6423	Comment	Dr. Abdusattar Sampe 32-03 CDCL3			Date	29 Mar 2021 03:27:44
Date Stamp	29 Mar 2021 03:27:44			File Name	C:\Users\hp\Desktop\Naproxen NMR\ NM16 28-03-2021\171\fid		
Frequency (MHz)	213.77	Nucleus	13C	Number of Transients	1024	Origin	spect
Original Points Count	32768	Owner	nmr	Points Count	32768	Pulse Sequence	zgpg30
Receiver Gain	186.93	SW(cyclical) (Hz)	51020.41	Solvent	CHLOROFORM-d		
Spectrum Offset (Hz)	21376.0332	Spectrum Type	STANDARD	Sweep Width (Hz)	51018.85	Temperature (degree C)	25.000

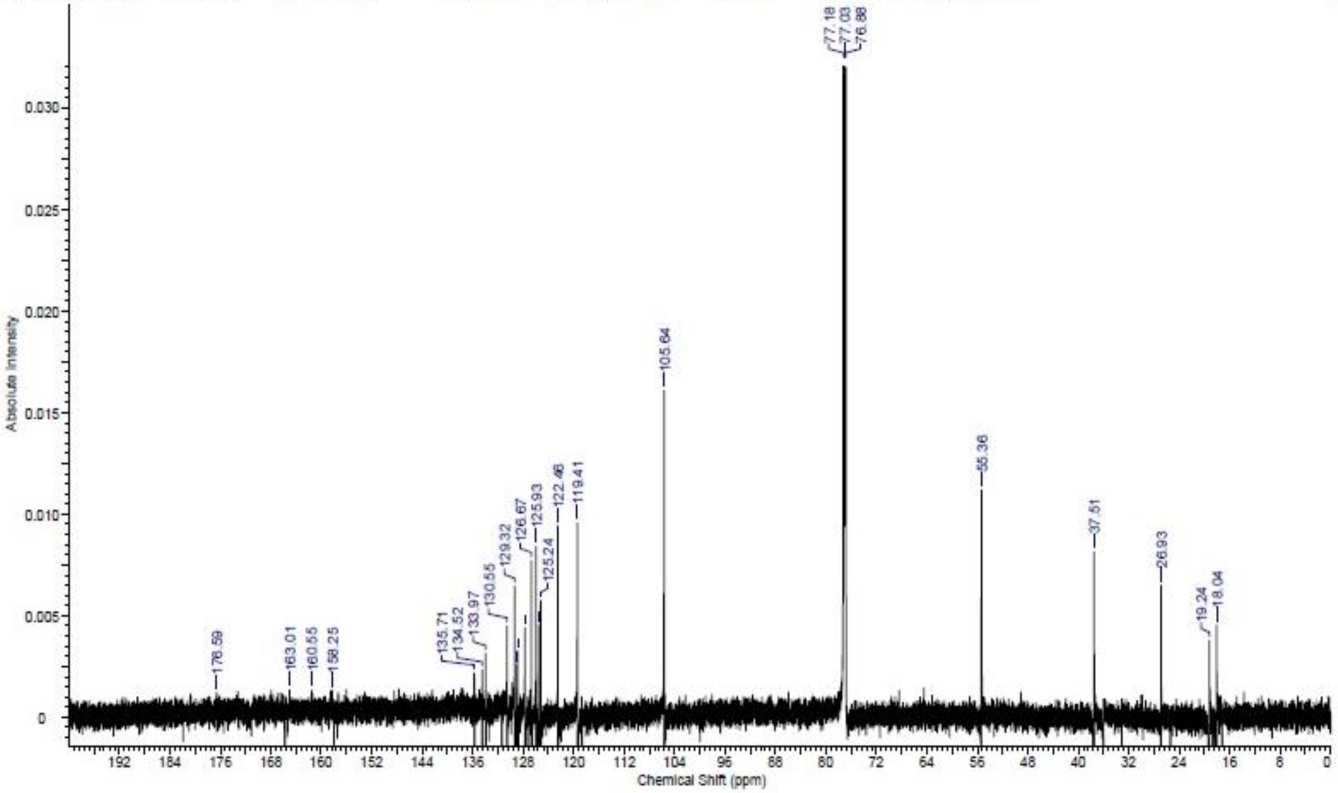

Compound 25

4/28/2021 11:42:53 PM

Acquisition Time (sec)	0.6423	*Comment*	Dr. Abdusattar Sampe 31-03 CDCL3			*Date*	29 Mar 2021 02:36:32
Date Stamp	29 Mar 2021 02:36:32			*File Name*	C:\Users\hp\Desktop\Naproxen NMR\NM15 28-03-2021\161\fid		
Frequency (MHz)	213.77	*Nucleus*	13C	*Number of Transients*	1024	*Origin*	spect
Original Points Count	32768	*Owner*	nmr	*Points Count*	32768	*Pulse Sequence*	zgpg30
Receiver Gain	186.93	*SW(cyclical) (Hz)*	51020.41	*Solvent*	CHLOROFORM-d		
Spectrum Offset (Hz)	21376.0332	*Spectrum Type*	STANDARD	*Sweep Width (Hz)*	51018.85	*Temperature (degree C)*	25.001

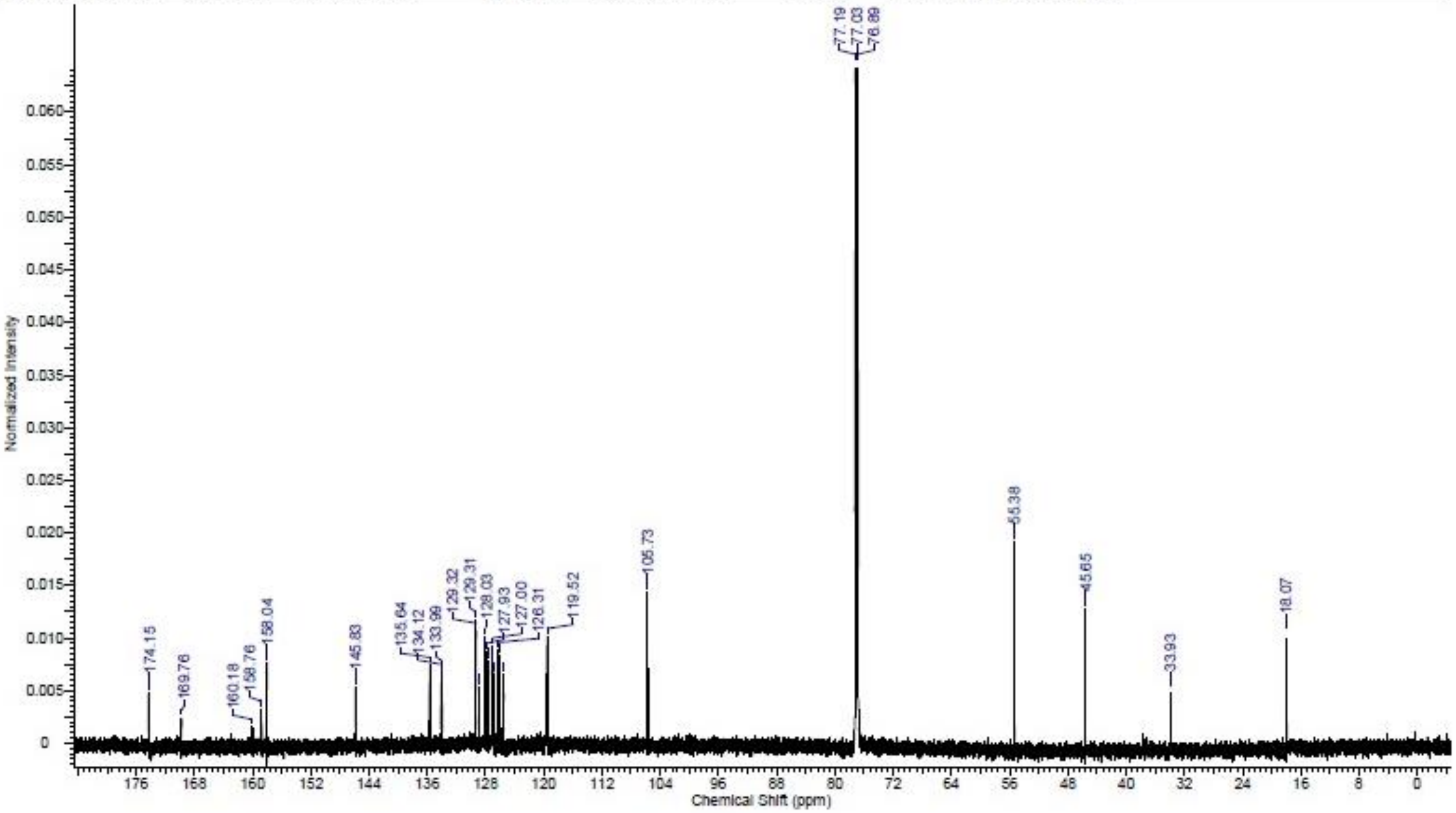

Compound 26

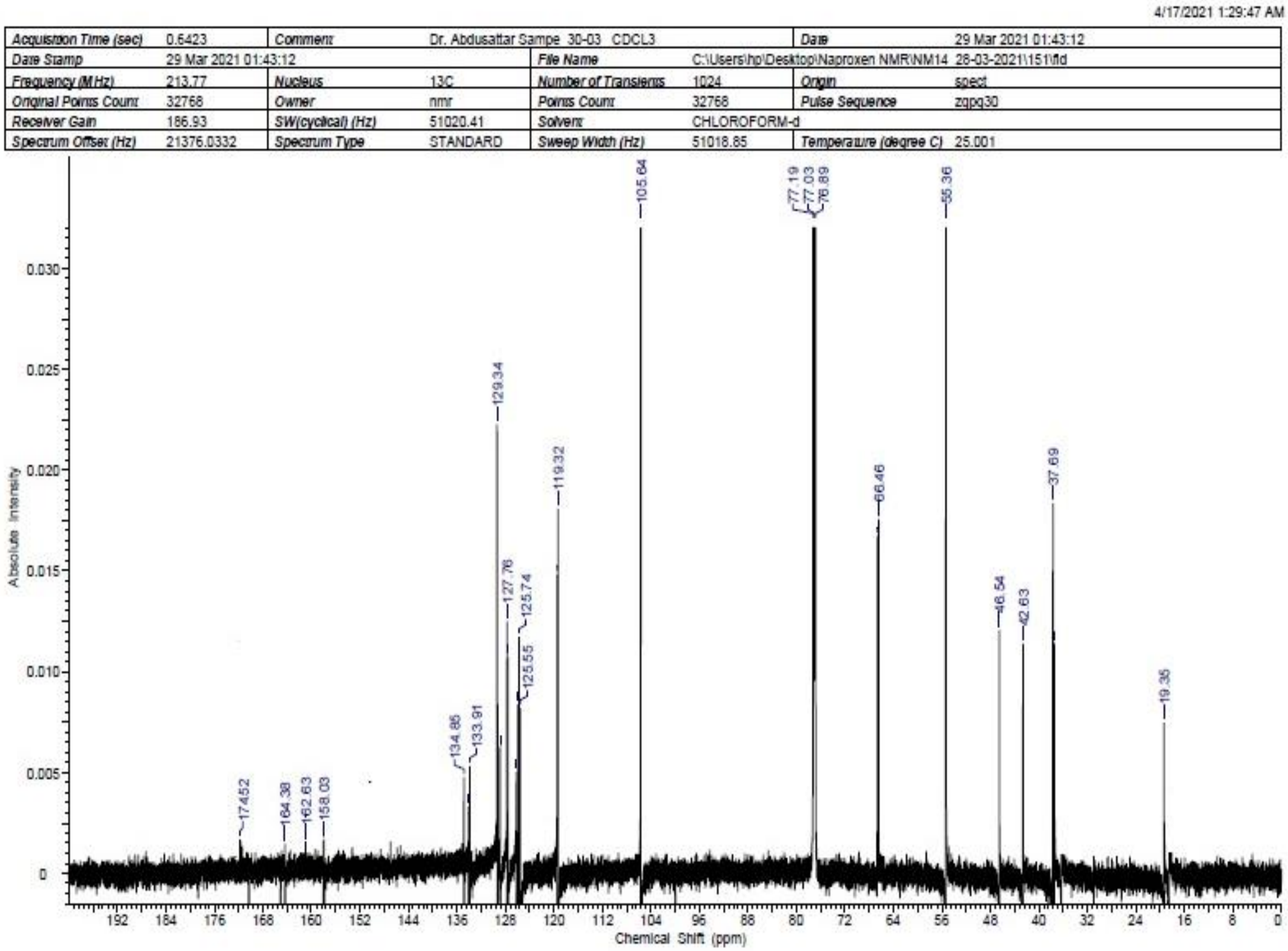
4/17/2021 1:29:47 AM
Acquisition Time (sec) 0.6423
Comment Dr. Abdusattar Sampe 30-03 CDCL3
Date 29 Mar 2021 01:43:12
Date Stamp 29 Mar 2021 01:43:12
File Name C:\Users\hp\Desktop\Naproxen NMR\NM14 28-03-2021\151\fid
Frequency (MHz) 213.77
Nucleus 13C
Number of Transients 1024
Origin spect
Original Points Count 32768
Owner nmr
Points Count 32768
Pulse Sequence zgpg30
Receiver Gain 186.93
SW(cyclical) (Hz) 51020.41
Solvent CHLOROFORM-d
Spectrum Offset (Hz) 21376.0332
Spectrum Type STANDARD
Sweep Width (Hz) 51018.85
Temperature (degree C) 25.001
105.64
77.19
77.03
76.89
55.36
129.34
119.32
66.46
37.69
127.76
125.74
125.55
46.54
42.63
19.35
134.86
133.91
174.52
164.38
162.63
158.03
Absolute Intensity
Chemical Shift (ppm)

Figure S35-S50-: Mass spectra of compounds

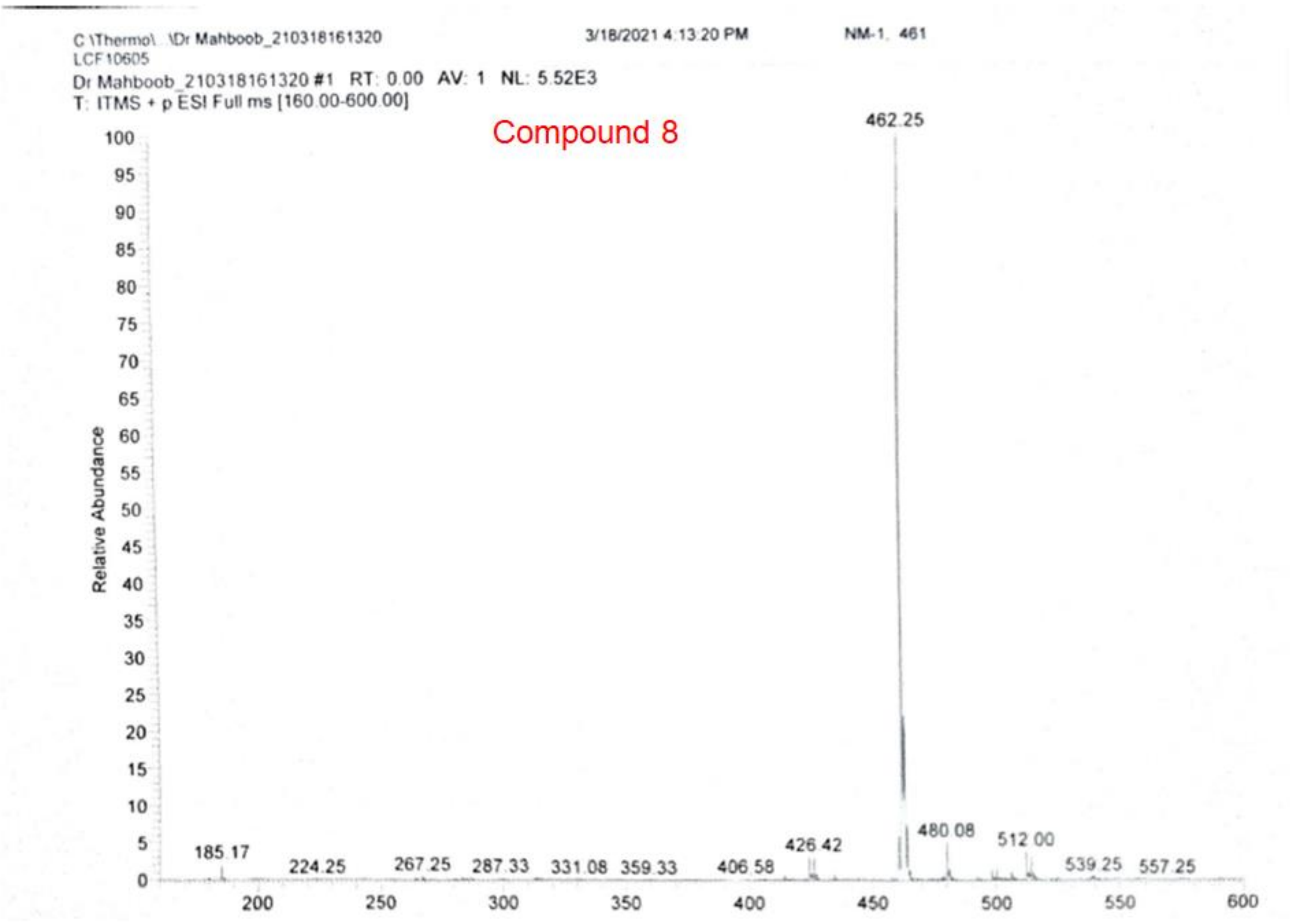

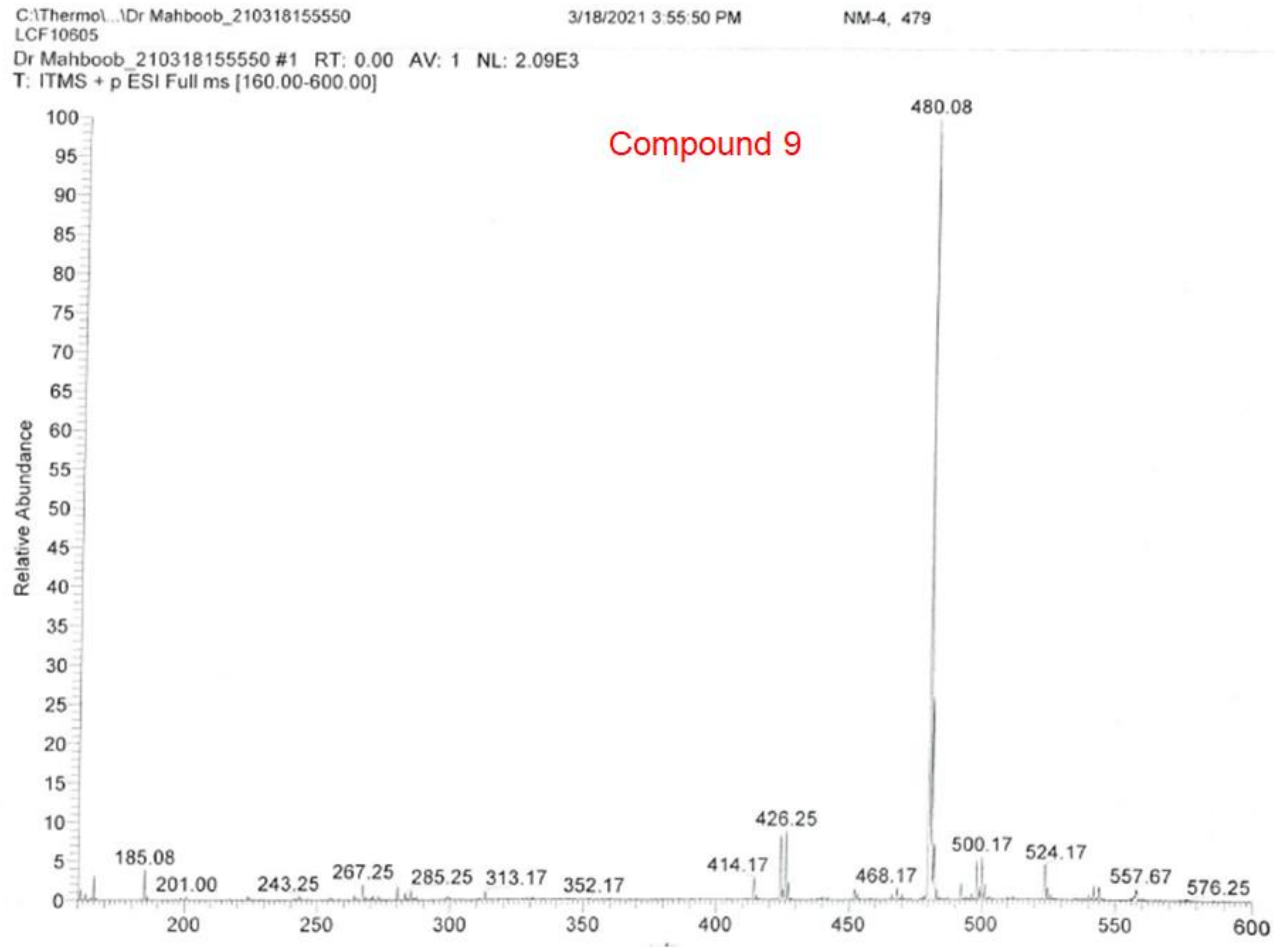
C:\Thermo\...\Dr Mahboob_210318155550
LCF10605
3/18/2021 3:55:50 PM
NM-4, 479
Dr Mahboob_210318155550 #1 RT: 0.00 AV: 1 NL: 2.09E3
T: ITMS + p ESI Full ms [160.00-600.00]
Compound 9
480.08
426.25
414.17
468.17
500.17
524.17
557.67
576.25
185.08
201.00
243.25
267.25
285.25
313.17
352.17
Relative Abundance
100
95
90
85
80
75
70
65
60
55
50
45
40
35
30
25
20
15
10
5
0
200
250
300
350
400
450
500
550
600

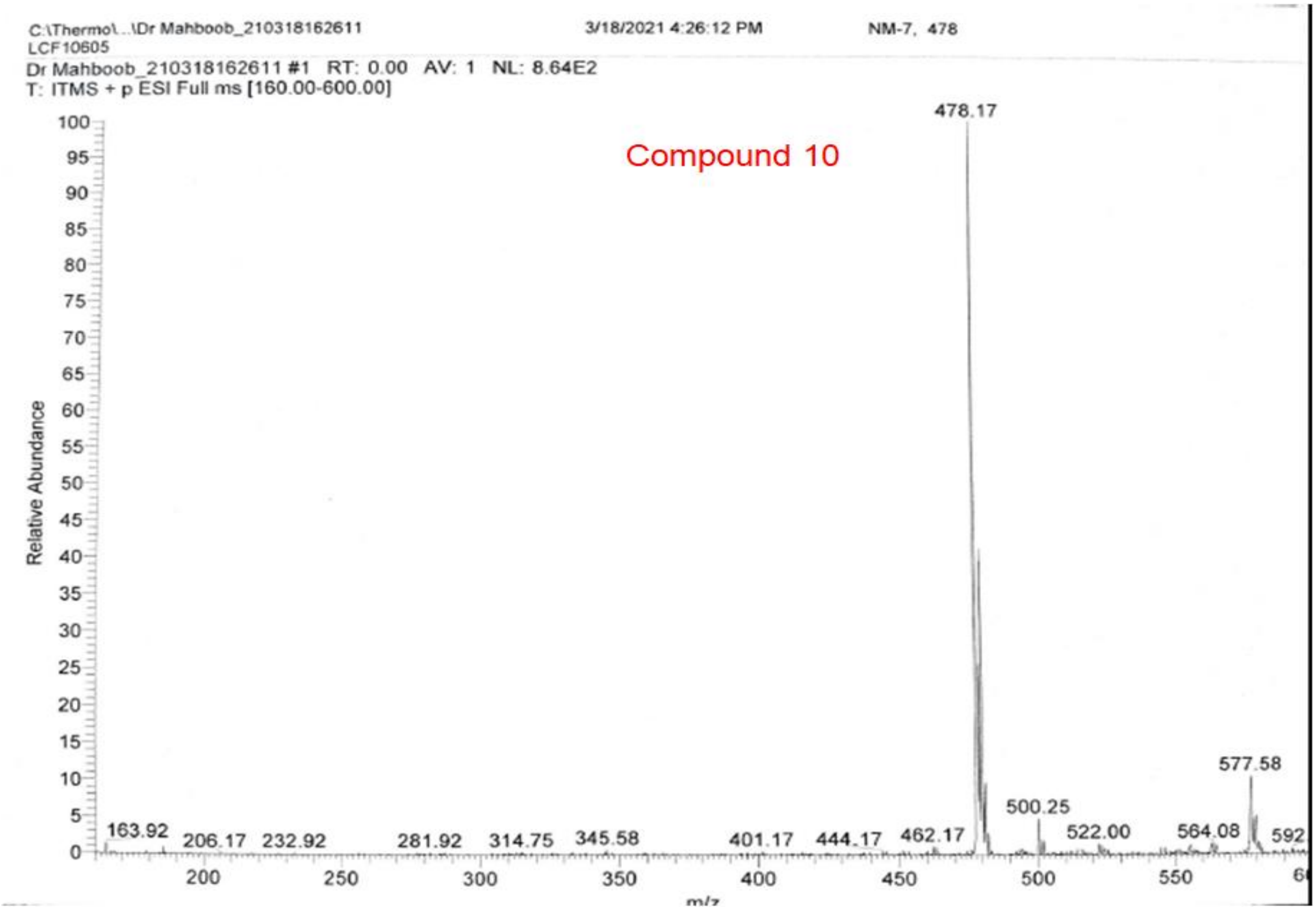
C:\Thermo\...\Dr Mahboob_210318162611
LCF10605
3/18/2021 4:26:12 PM
NM-7, 478
Dr Mahboob_210318162611 #1 RT: 0.00 AV: 1 NL: 8.64E2
T: ITMS + p ESI Full ms [160.00-600.00]
Compound 10
478.17
577.58
500.25
522.00
564.08
163.92
206.17
232.92
281.92
314.75
345.58
401.17
444.17
462.17
Relative Abundance
m/z

Compound 11

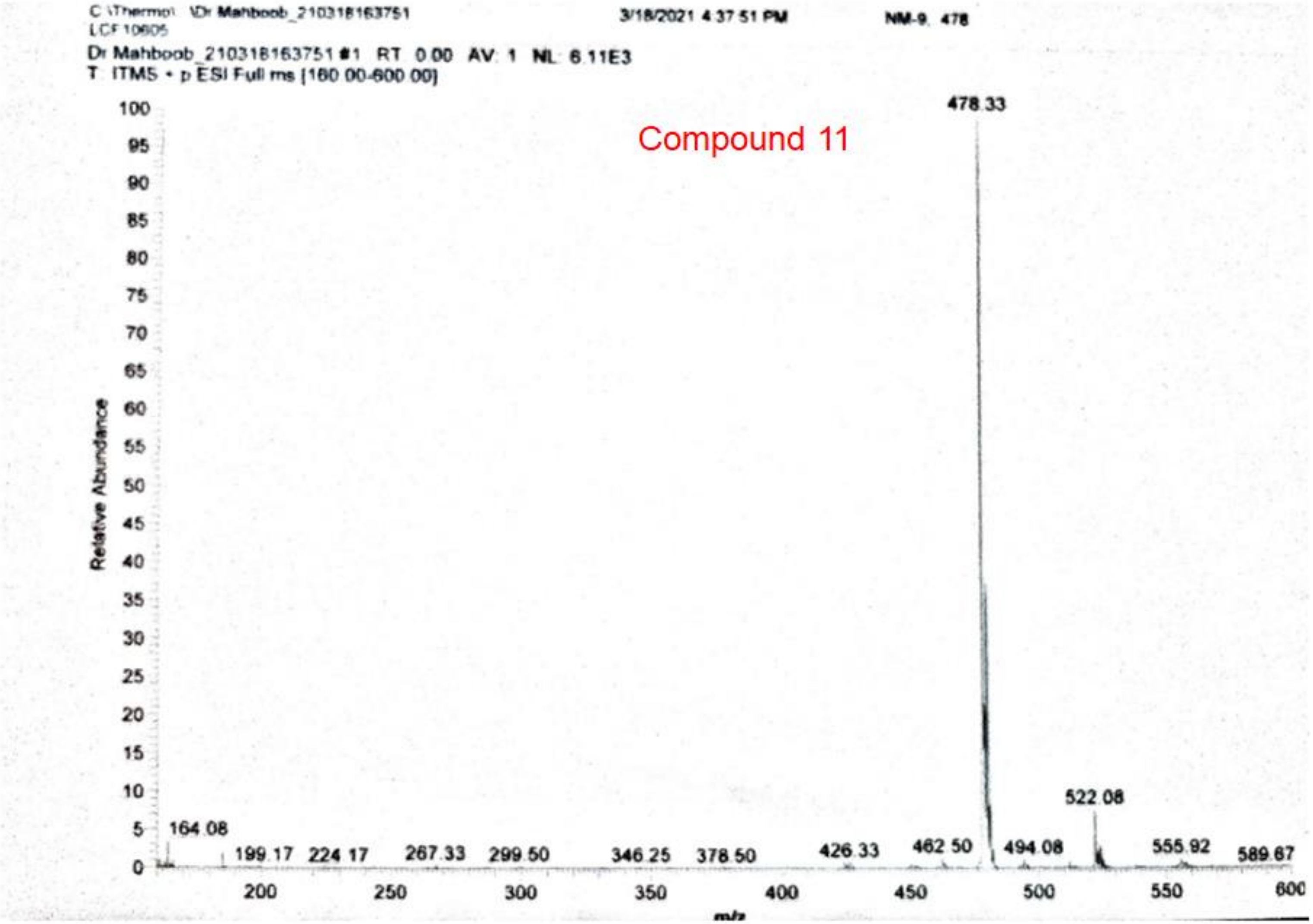
C:\Thermo\ \Dr Mahboob_210318163751
LCF10605
3/18/2021 4:37:51 PM
NM-9, 478
Dr Mahboob_210318163751 #1 RT: 0.00 AV: 1 NL: 6.11E3
T: ITMS + p ESI Full ms [160.00-600.00]
Relative Abundance
m/z
478.33
522.08
164.08
199.17
224.17
267.33
299.50
346.25
378.50
426.33
462.50
494.08
555.92
589.67

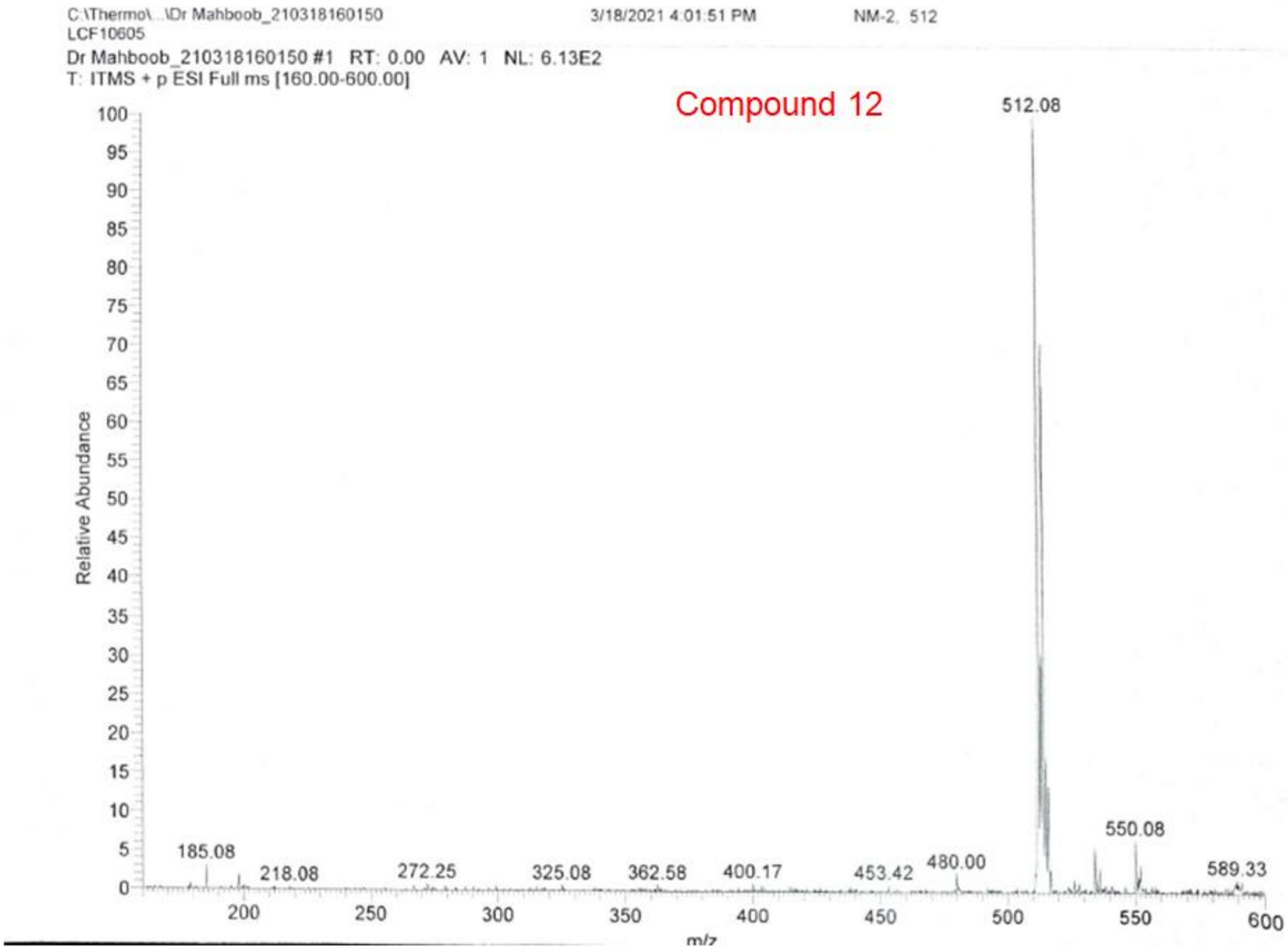
C:\Thermo\...\Dr Mahboob_210318160150
LCF10605
3/18/2021 4:01:51 PM
NM-2, 512
Dr Mahboob_210318160150 #1 RT: 0.00 AV: 1 NL: 6.13E2
T: ITMS + p ESI Full ms [160.00-600.00]
Compound 12
512.08
550.08
589.33
480.00
453.42
400.17
362.58
325.08
272.25
218.08
185.08
Relative Abundance
m/z

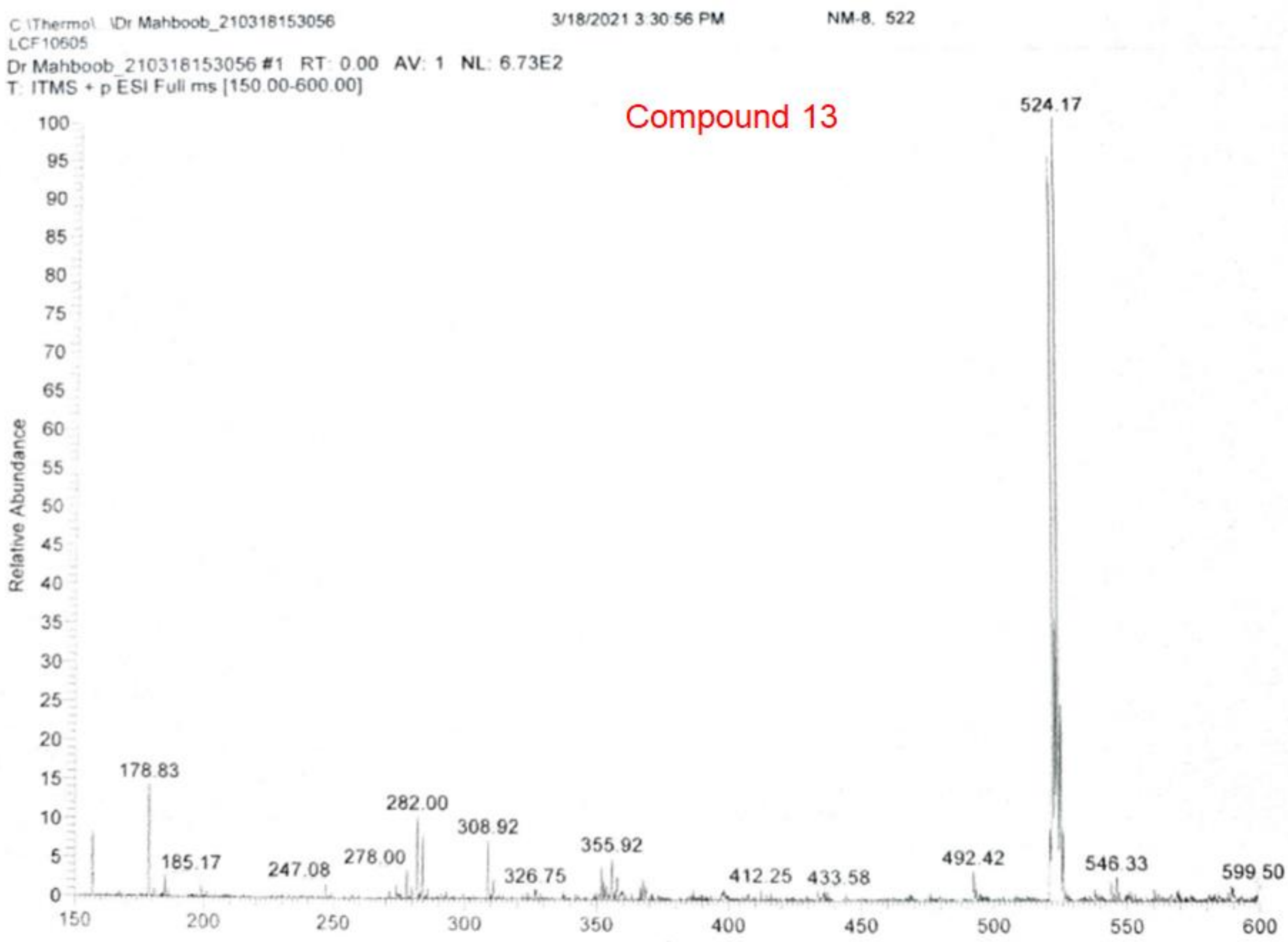

C:\Thermo\...\Dr Mahboob_210318153056
3/18/2021 3:30:56 PM
NM-8, 522
LCF10605
Dr Mahboob_210318153056 #1 RT: 0.00 AV: 1 NL: 6.73E2
T: ITMS + p ESI Full ms [150.00-600.00]
Compound 13
524.17
178.83
185.17
247.08
278.00
282.00
308.92
326.75
355.92
412.25
433.58
492.42
546.33
599.50
Relative Abundance

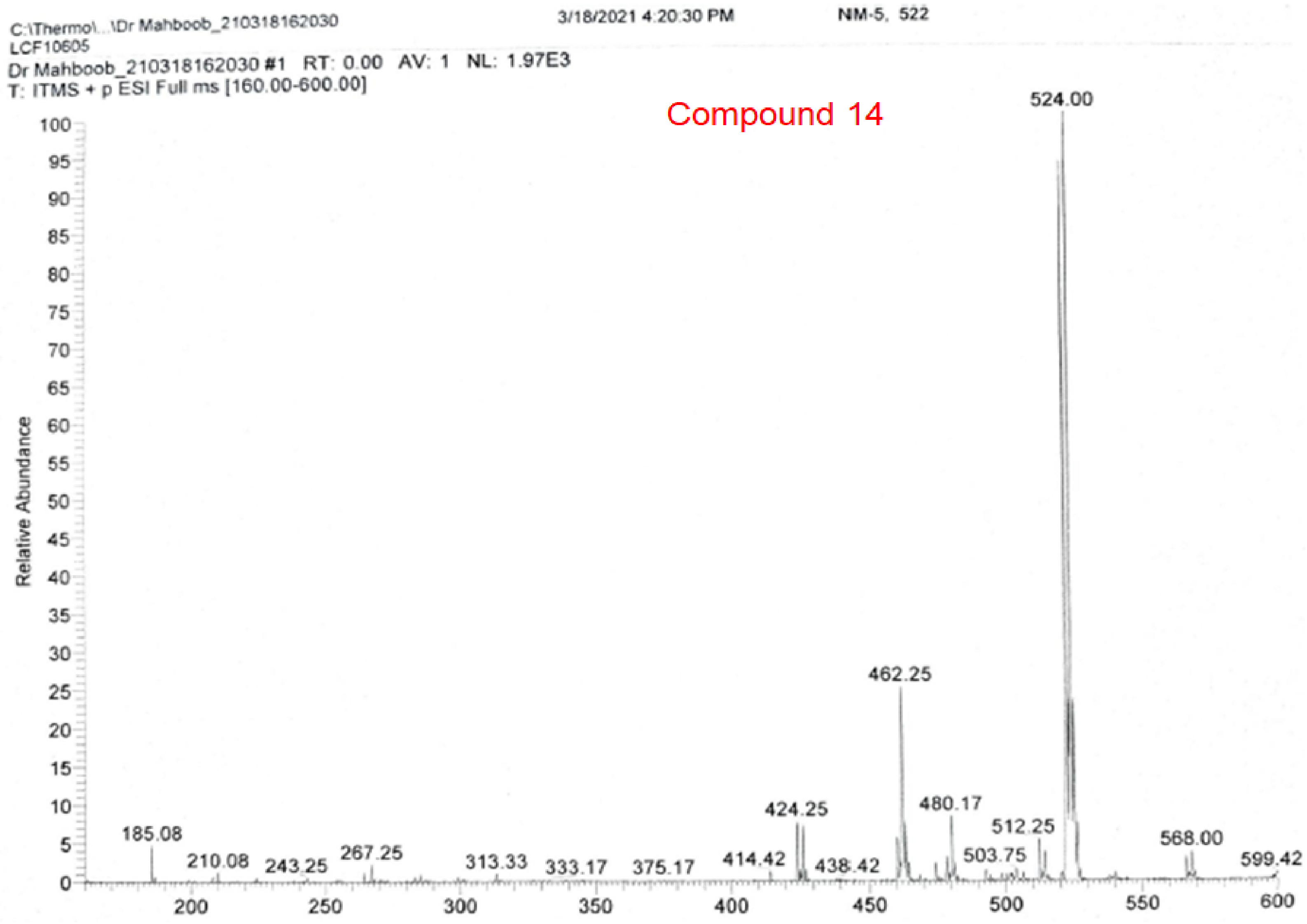
C:\Thermo\...\Dr Mahboob_210318162030
3/18/2021 4:20:30 PM
NM-5, 522
LCF10605
Dr Mahboob_210318162030 #1 RT: 0.00 AV: 1 NL: 1.97E3
T: ITMS + p ESI Full ms [160.00-600.00]
Compound 14
524.00
462.25
480.17
424.25
512.25
185.08
210.08
243.25
267.25
313.33
333.17
375.17
414.42
438.42
503.75
568.00
599.42
Relative Abundance

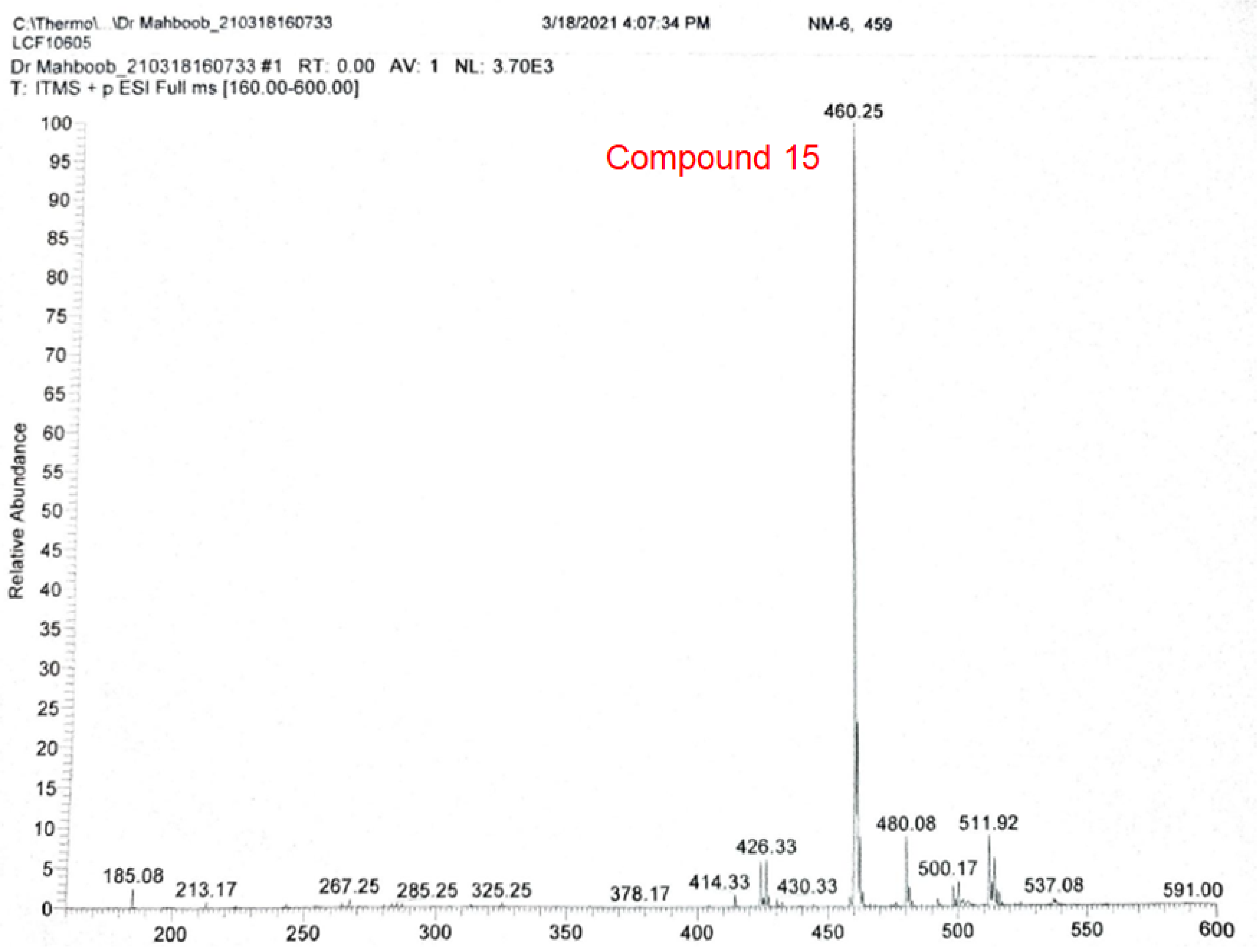
C:\Thermo\...\Dr Mahboob_210318160733
LCF10605
3/18/2021 4:07:34 PM
NM-6, 459
Dr Mahboob_210318160733 #1 RT: 0.00 AV: 1 NL: 3.70E3
T: ITMS + p ESI Full ms [160.00-600.00]
Compound 15
460.25
480.08
511.92
426.33
500.17
185.08
213.17
267.25
285.25
325.25
378.17
414.33
430.33
537.08
591.00
Relative Abundance
100
95
90
85
80
75
70
65
60
55
50
45
40
35
30
25
20
15
10
5
0
200
250
300
350
400
450
500
550
600

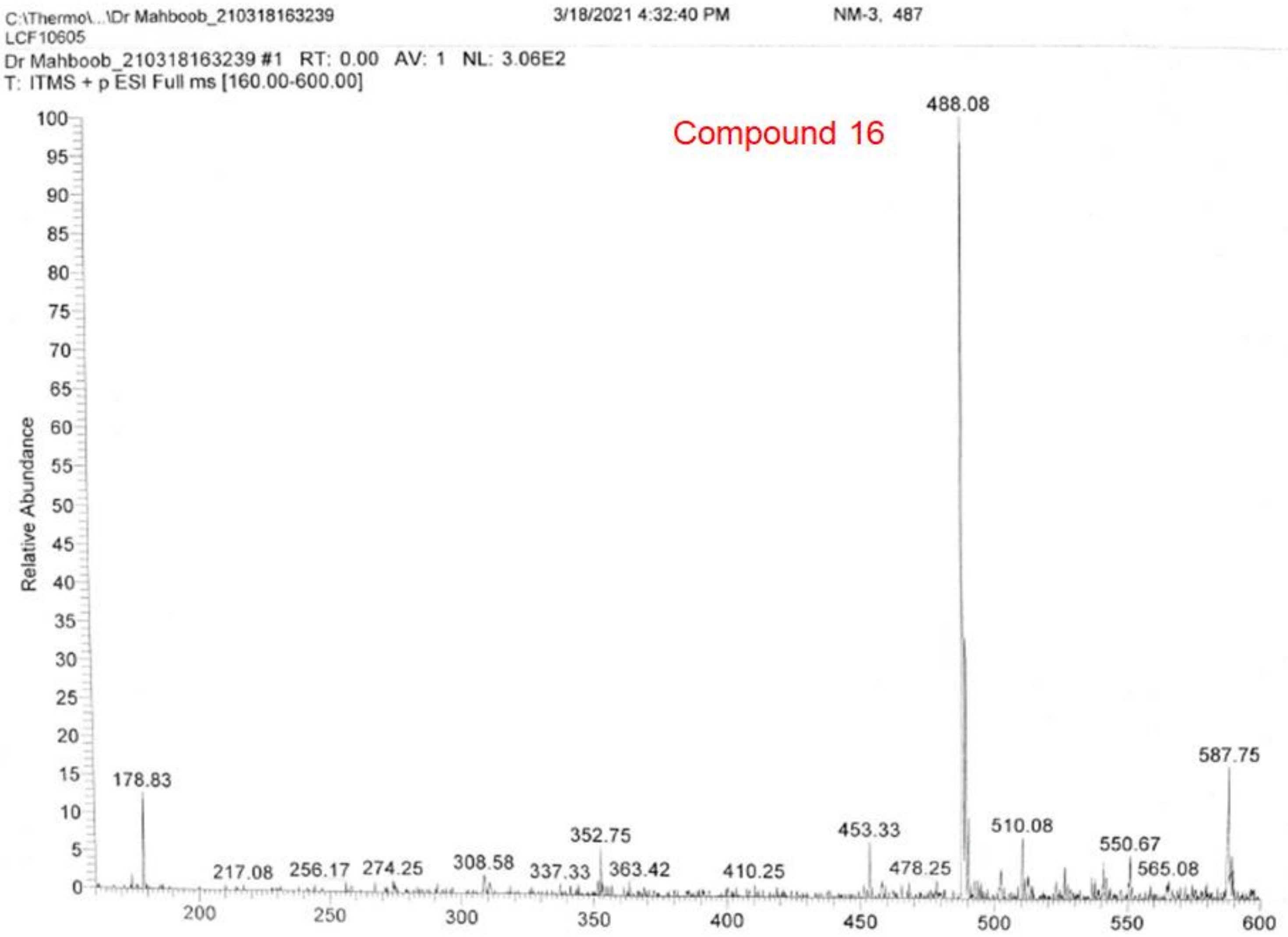

C:\Thermo\...\Dr Mahboob_210318163239
3/18/2021 4:32:40 PM
NM-3, 487
LCF10605
Dr Mahboob_210318163239 #1 RT: 0.00 AV: 1 NL: 3.06E2
T: ITMS + p ESI Full ms [160.00-600.00]
Compound 16
Relative Abundance
488.08
587.75
178.83
352.75
453.33
510.08
550.67
565.08
217.08
256.17
274.25
308.58
337.33
363.42
410.25
478.25

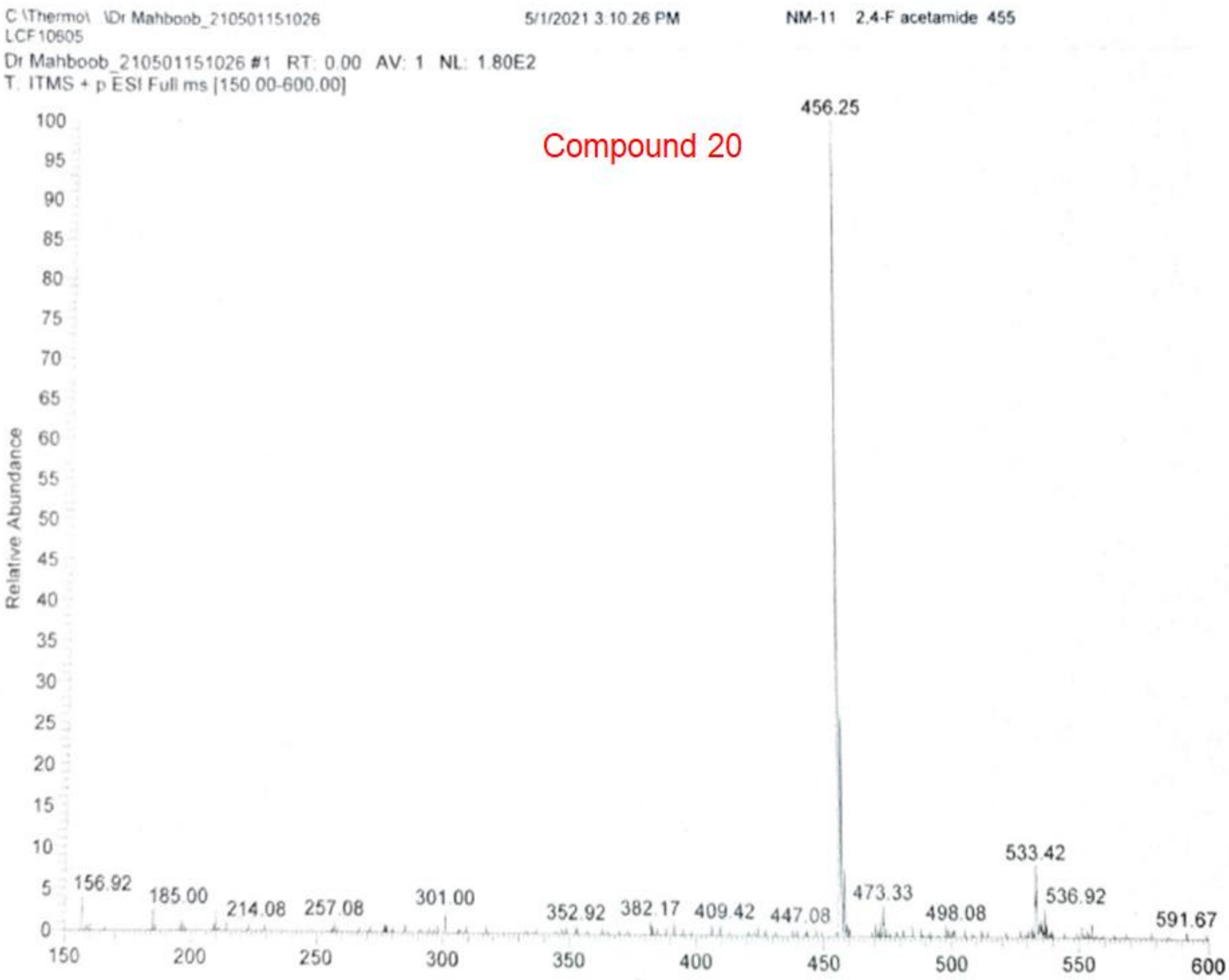
C:\Thermo\ \Dr Mahboob_210501151026
LCF10605
5/1/2021 3:10:26 PM
NM-11 2,4-F acetamide 455
Dr Mahboob_210501151026 #1 RT: 0.00 AV: 1 NL: 1.80E2
T: ITMS + p ESI Full ms [150.00-600.00]
Compound 20
456.25
533.42
536.92
473.33
498.08
591.67
156.92
185.00
214.08
257.08
301.00
352.92
382.17
409.42
447.08
Relative Abundance
100
95
90
85
80
75
70
65
60
55
50
45
40
35
30
25
20
15
10
5
0
150
200
250
300
350
400
450
500
550
600

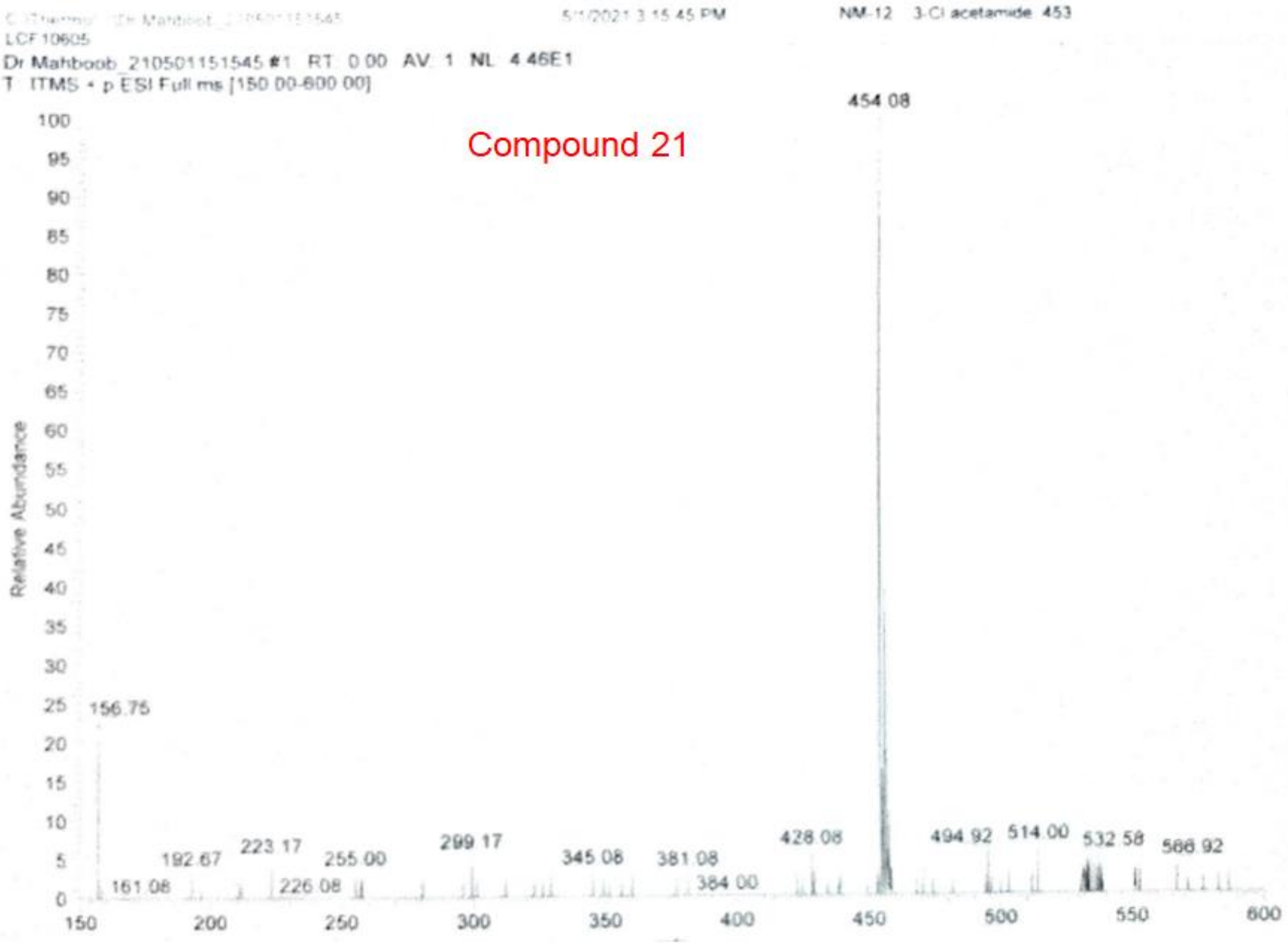
5/1/2021 3:15:45 PM
NM-12 3-Cl acetamide 453
LCF10605
Dr Mahboob_210501151545 #1 RT: 0.00 AV: 1 NL: 4.46E1
T: ITMS + p ESI Full ms [150.00-600.00]
Compound 21
Relative Abundance
454.08
156.75
161.08
192.67
223.17
226.08
255.00
299.17
345.08
381.08
384.00
428.08
494.92
514.00
532.58
566.92

C:\Thermo\...\Dr Mahboob_210501152204 5/1/2021 3:22:04 PM NM-13 3-Bracetamide 497
LCF10605
Dr Mahboob_210501152204 #1 RT: 0.00 AV: 1 NL: 5.41E1
T: ITMS + p ESI Full ms [150.00-600.00]

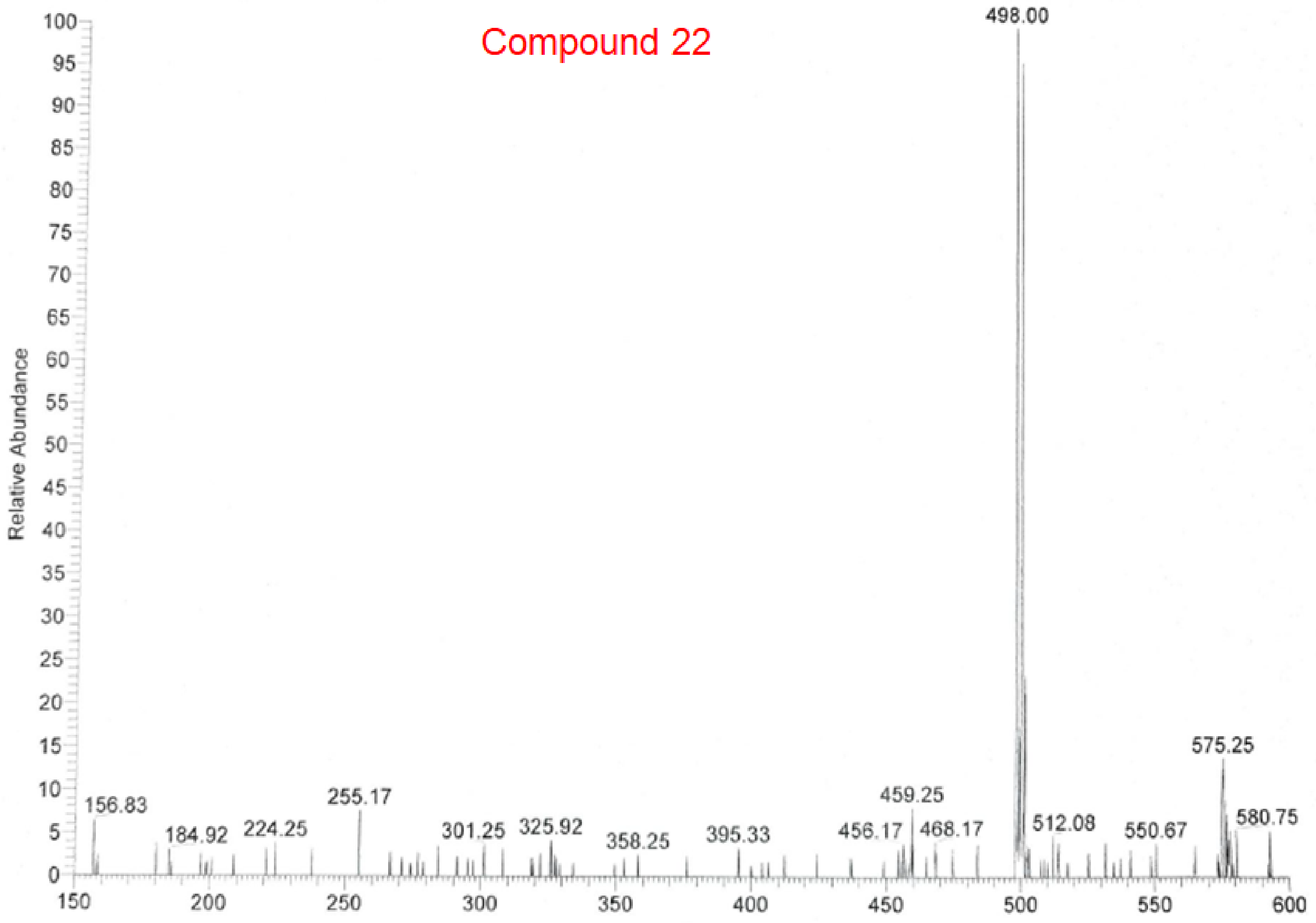

C:\Thermo\...\Dr Mahboob_210501153309
LCF10605

5/1/2021 3:33:09 PM

NM-17 4-Br acetamide 497

Dr Mahboob_210501153309 #1 RT: 0.00 AV: 1 NL: 7.48E1
T: ITMS + p ESI Full ms [150.00-600.00]

Compound 23

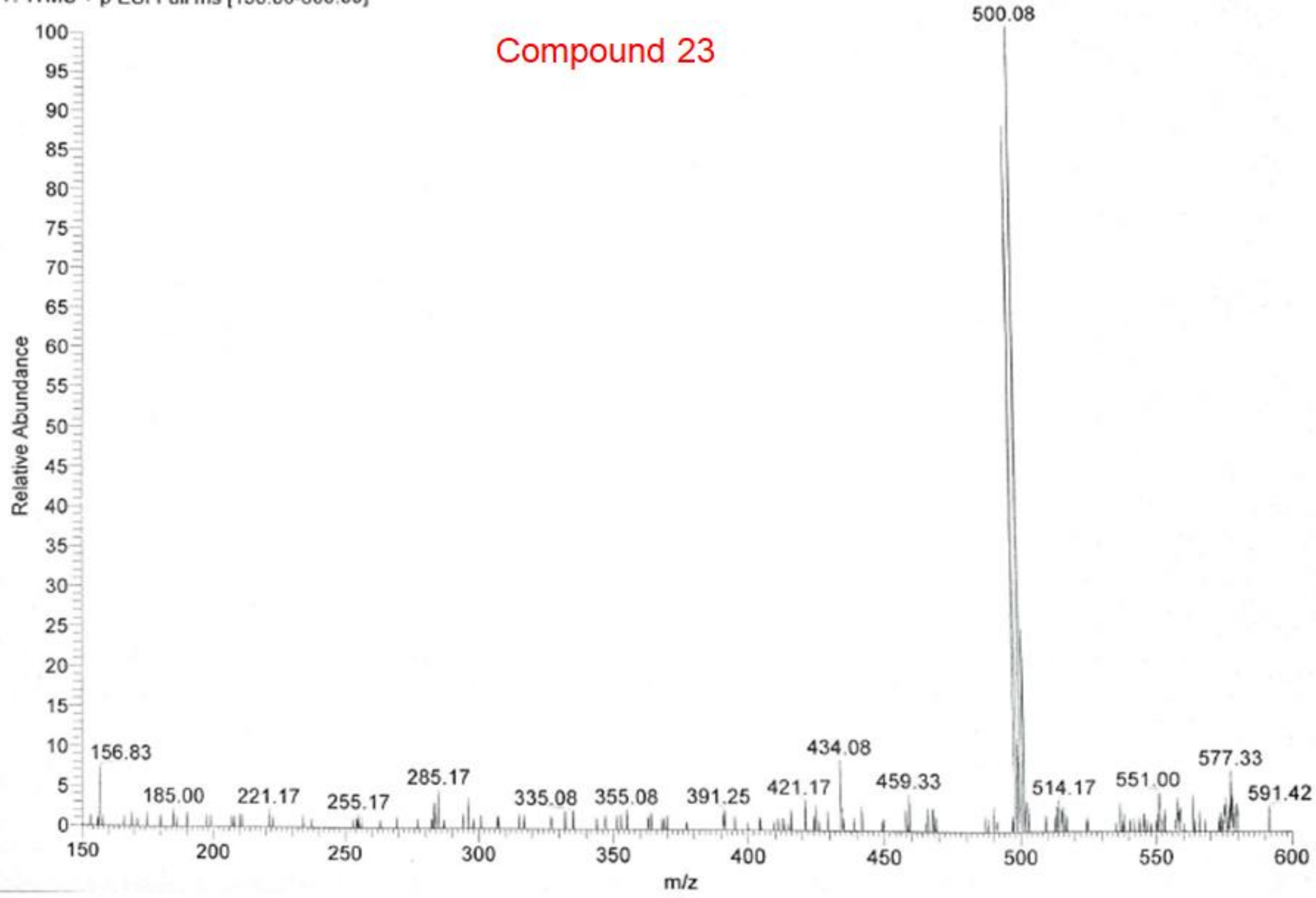

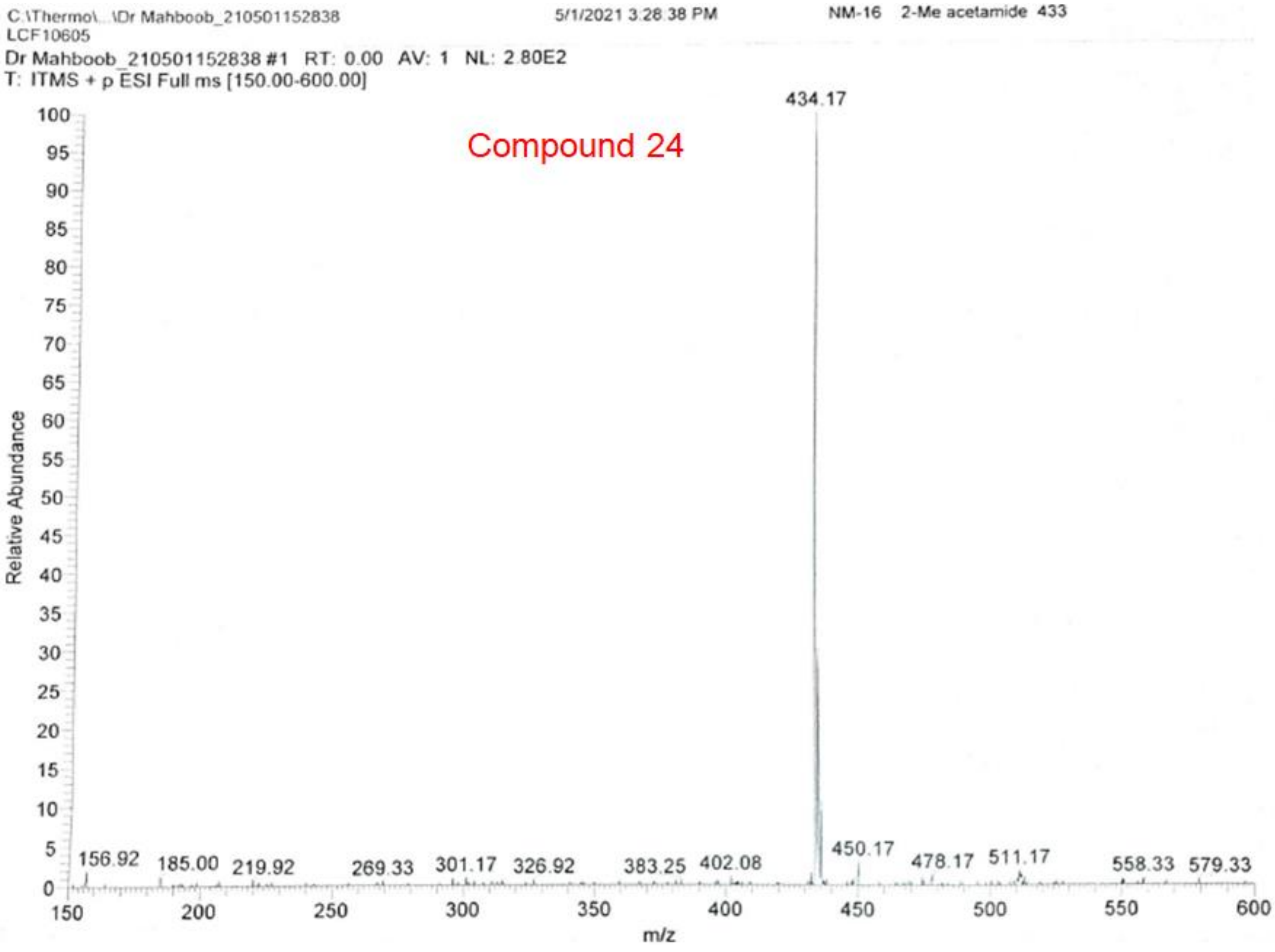
C:\Thermo\...\Dr Mahboob_210501152838
LCF10605
5/1/2021 3:28:38 PM
NM-16 2-Me acetamide 433
Dr Mahboob_210501152838 #1 RT: 0.00 AV: 1 NL: 2.80E2
T: ITMS + p ESI Full ms [150.00-600.00]
Compound 24
434.17
156.92 185.00 219.92 269.33 301.17 326.92 383.25 402.08 450.17 478.17 511.17 558.33 579.33
Relative Abundance
m/z

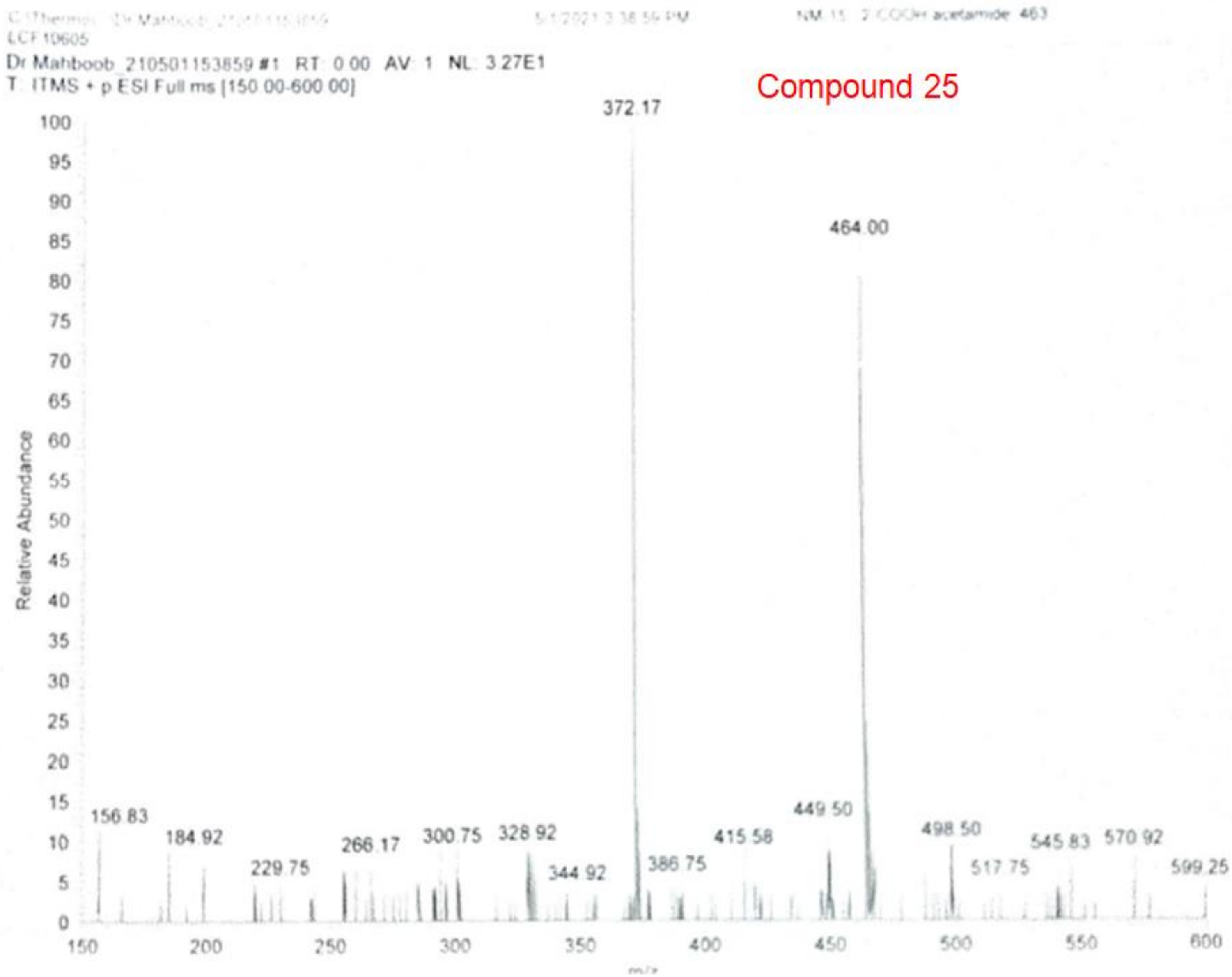
Dr Mahboob_210501153859 #1 RT: 0.00 AV: 1 NL: 3.27E1
T: ITMS + p ESI Full ms [150.00-600.00]
Compound 25
372.17
464.00
156.83
184.92
229.75
266.17
300.75
328.92
344.92
386.75
415.58
449.50
498.50
517.75
545.83
570.92
599.25
Relative Abundance
m/z

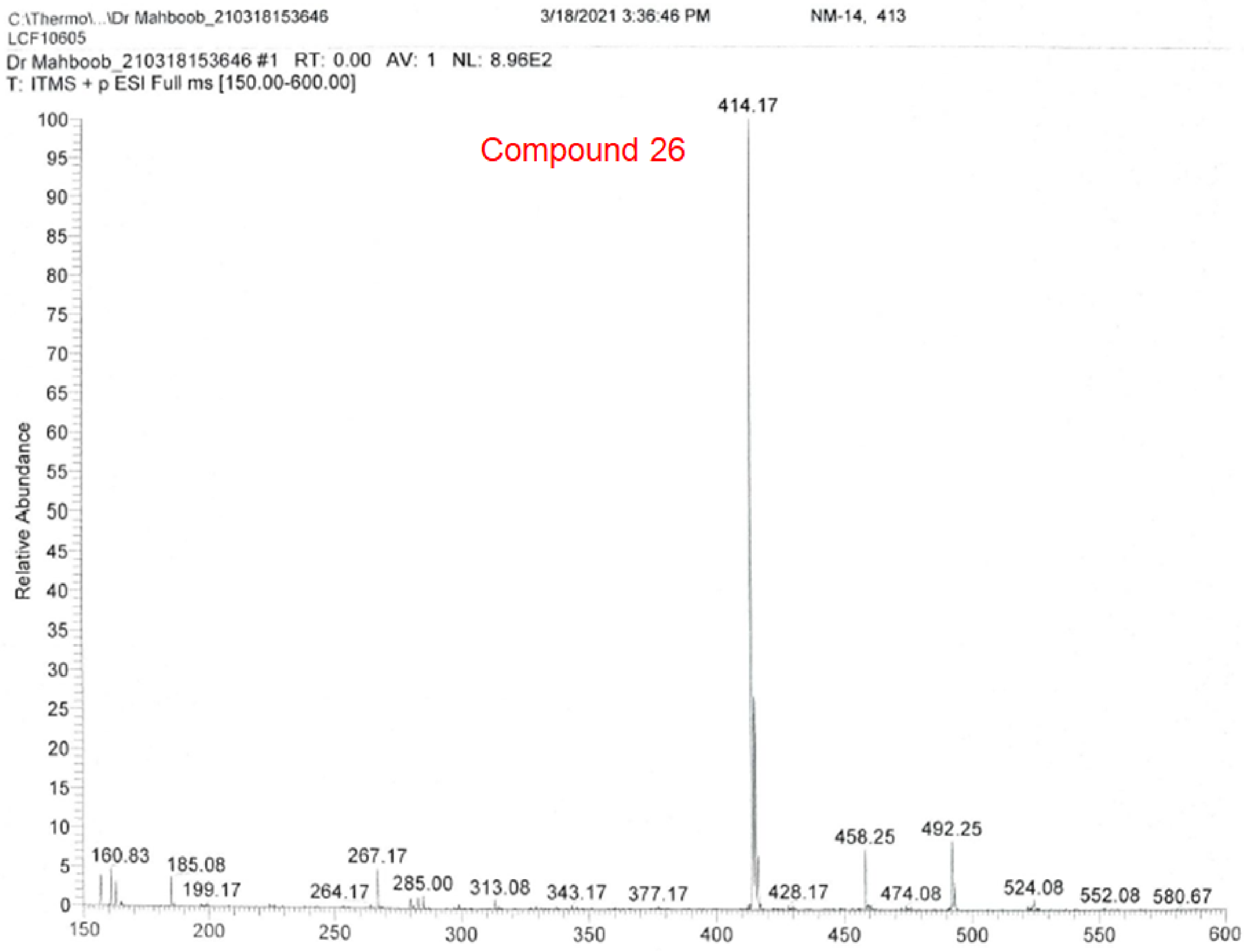
C:\Thermo\...\Dr Mahboob_210318153646
3/18/2021 3:36:46 PM
NM-14, 413
LCF10605
Dr Mahboob_210318153646 #1 RT: 0.00 AV: 1 NL: 8.96E2
T: ITMS + p ESI Full ms [150.00-600.00]
Compound 26
Relative Abundance
414.17
458.25
492.25
160.83
185.08
199.17
264.17
267.17
285.00
313.08
343.17
377.17
428.17
474.08
524.08
552.08
580.67

ALIE is represented as sum $E_{orbitals}$ weighted by the orbital densities (supplementary material) according equation:

$(I(r) = \sum_i \frac{pi(\vec{r})\,|\varepsilon i|}{p(\vec{r})})$. Where $I(r) = \sum_i \frac{pi(\vec{r})\,|\varepsilon i|}{p(\vec{r})}$ where $pi(\vec{r})$:is electronic cloud for the orbital (i) at the exact point $(\vec{r}), |\varepsilon i|$: is energy orbital and $p(\vec{r})$: reflecting the summation electronic density function.
